# Single-cell Sequencing of *Thiomargarita* Reveals Genomic Flexibility for Adaptation to Dynamic Redox Conditions

**DOI:** 10.3389/fmicb.2016.00964

**Published:** 2016-06-21

**Authors:** Matthias Winkel, Verena Salman-Carvalho, Tanja Woyke, Michael Richter, Heide N. Schulz-Vogt, Beverly E. Flood, Jake V. Bailey, Marc Mußmann

**Affiliations:** ^1^Molecular Ecology Group, Department of Molecular Ecology, Max Planck Institute for Marine MicrobiologyBremen, Germany; ^2^Section Geomicrobiology, GFZ German Research Centre for Geoscience, Helmholtz Centre PotsdamPotsdam, Germany; ^3^HGF MPG Joint Research Group for Deep-sea Ecology and Technology, Max Planck Institute for Marine MicrobiologyBremen, Germany; ^4^Department of Energy Joint Genome Institute, Walnut CreekCA, USA; ^5^Microbial Genomics and Bioinformatics Group, Department of Molecular Ecology, Max Planck Institute for Marine MicrobiologyBremen, Germany; ^6^Leibniz-Institut für Ostseeforschung WarnemündeRostock, Germany; ^7^Department of Earth Sciences, University of Minnesota, MinneapolisMN, USA

**Keywords:** “*Candidatus* Thiomargarita nelsonii”, single-cell genome, sulfur-oxidizing bacteria, cyanobacteria, multiple-displacement amplification, C2-cycle

## Abstract

Large, colorless sulfur-oxidizing bacteria (LSB) of the family *Beggiatoaceae* form thick mats at sulfidic sediment surfaces, where they efficiently detoxify sulfide before it enters the water column. The genus *Thiomargarita* harbors the largest known free-living bacteria with cell sizes of up to 750 μm in diameter. In addition to their ability to oxidize reduced sulfur compounds, some *Thiomargarita* spp. are known to store large amounts of nitrate, phosphate and elemental sulfur internally. To date little is known about their energy yielding metabolic pathways, and how these pathways compare to other *Beggiatoaceae*. Here, we present a draft single-cell genome of a chain-forming “*Candidatus* Thiomargarita nelsonii Thio36”, and conduct a comparative analysis to five draft and one full genome of other members of the *Beggiatoaceae*. “*Ca*. T. nelsonii Thio36” is able to respire nitrate to both ammonium and dinitrogen, which allows them to flexibly respond to environmental changes. Genes for sulfur oxidation and inorganic carbon fixation confirmed that “*Ca*. T. nelsonii Thio36” can function as a chemolithoautotroph. Carbon can be fixed via the Calvin–Benson–Bassham cycle, which is common among the *Beggiatoaceae*. In addition we found key genes of the reductive tricarboxylic acid cycle that point toward an alternative CO_2_ fixation pathway. Surprisingly, “*Ca*. T. nelsonii Thio36” also encodes key genes of the C2-cycle that convert 2-phosphoglycolate to 3-phosphoglycerate during photorespiration in higher plants and cyanobacteria. Moreover, we identified a novel trait of a flavin-based energy bifurcation pathway coupled to a Na^+^-translocating membrane complex (Rnf). The coupling of these pathways may be key to surviving long periods of anoxia. As other *Beggiatoaceae* “*Ca*. T. nelsonii Thio36” encodes many genes similar to those of (filamentous) cyanobacteria. In summary, the genome of “*Ca*. T. nelsonii Thio36” provides additional insight into the ecology of giant sulfur-oxidizing bacteria, and reveals unique genomic features for the *Thiomargarita* lineage within the *Beggiatoaceae*.

## Introduction

Large colorless sulfur-oxidizing bacteria (LSB) are globally distributed and typically occur at the surface of sulfidic sediments. Here, opposed gradients of oxygen and sulfide (Jørgensen and Revsbech, 1983) favor the formation of dense populations of, e.g., *Beggiatoa*, *Thioploca*, and *Thiomargarita* (Jannasch et al., 1989; Fossing et al., 1995; Schulz et al., 1999). Both, marine and freshwater LSB oxidize various forms of reduced sulfur compounds (sulfide, thiosulfate, elemental sulfur), while some also use smaller organic compounds, such as acetate, lactate, and ethanol as energy source (Teske and Salman, 2014).

LSB commonly store elemental sulfur and typically occur as filaments or single cells within the top centimeters of the sediment. By their gliding or rolling motility, some LSB migrate between the deeper and surficial layers of the sediment, thereby bridging the gap between available electron donors and acceptors in the different sediment layers. In this manner, these LSB may gain a competitive advantage over other sulfur-oxidizing bacteria (SOB), which require a simultaneous access to both, electron donor and acceptor (Jørgensen and Gallardo, 1999). Sometimes, *Thiomargarita* and *Beggiatoa* co-exist in mats, which was attributed to a niche separation in these metabolically similar microorganisms (Grünke et al., 2011). While the ecological niches of LSB have been frequently studied (Teske and Salman, 2014 and reference therein), still little is known about how these distinct niches are reflected in the pan-genome of the *Beggiatoaceae* and even less is known about the genomes of the non-filamentous, mainly non-motile genus *Thiomargarita*.

*Thiomargarita* spp. were originally discovered in the upper most centimeters of an organic-rich diatomaceous ooze deposited beneath the Benguela upwelling system off the coast of Namibia (Schulz et al., 1999). The underlying sediments contain extremely high sulfide concentrations of up to 22 mM (Brüchert et al., 2003). The enormous oxygen demand in this eutrophic and sulfidic system causes oxygen depletion in the sediment itself as well as in the overlying waters, and forces *Thiomargarita* to use electron acceptors other than oxygen. Nitrate is stored at 0.1–0.8 M concentrations in a large central vacuole that occupies 98% of the cell volume (Schulz et al., 1999). Also other members of the family *Beggiatoaceae* store nitrate in a central vacuole, and use it as alternative electron acceptor under anoxic conditions (Fossing et al., 1995; McHatton et al., 1996). Experimental evidence suggest that some freshwater and marine *Beggiatoa* spp. and *Thioploca* spp. perform dissimilatory reduction of nitrate to ammonium (Vargas and Strohl, 1985; McHatton et al., 1996; Otte et al., 1999), while other marine LSB perform denitrification (Sweerts et al., 1990; Mußmann et al., 2007; Beutler et al., 2012). A cryptic nitrogen cycle was even observed between denitrifiying marine *Thioploca* spp. filaments and their sheath-associated anaerobic ammonia-oxidizing (anammox) bacteria (Prokopenko et al., 2013). Another study showed the close association of marine *Beggiatoa* and aerobic ammonia-oxidizer to recycle nitrogen in highly fluctuating hydrothermal systems (Winkel et al., 2014). Besides genomic evidence for the different nitrate respiration pathways in LSB (Mußmann et al., 2007; MacGregor et al., 2013a), recent proteomic and transcriptomic analyses revealed the actual expression of denitrification genes in freshwater *Thioploca ingrica* (Kojima et al., 2015), and marine “*Ca*. Thiopilula sp.” (Jones et al., 2015).

In addition to storing nitrate, many LSB also store polyphosphates and polymeric carbon. For example, *Thiomargarita* stores glycogen (Schulz and Schulz, 2005), while filamentous *Beggiatoa* species store polyhydroxyalcanoate as carbon and energy reserve (Brock and Schulz-Vogt, 2011).

In recent years, additional *Thiomargarita* ecotypes have been detected in various marine sediments worldwide, including methane seeps and hydrothermal systems (Kalanetra et al., 2005; Bailey et al., 2011; Grünke et al., 2011; Salman et al., 2011). Cells of this genus represent the largest free-living bacteria that are typically non-motile, spherical, and sometimes form chains surrounded by a thick mucus sheath (Schulz et al., 1999). Due to their apparent immobility, *Thiomargarita* are thought to depend on temporal variations in the chemical environment or on physical transport by traction currents, gas bubbles, or, in some cases, animal hosts.

**FIGURE 1:**
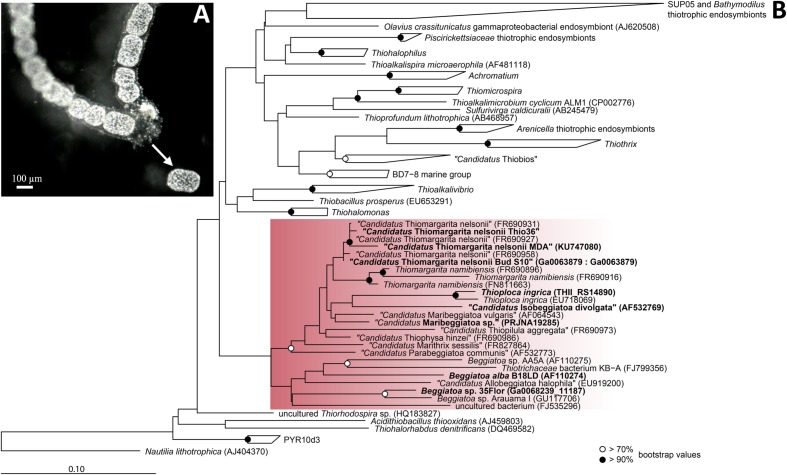
**Morpholgy and phylogeny of “*Candidatus* Thiomargarita nelsonii Thio36”.** **(A)** Chain-forming morphotype with cylindrical cells that served as template for single-cell genome amplification. Arrow indicates a cell that was manually separated from the surrounding mucus sheath. **(B)** Phylogenetic tree of the 16S rRNA gene of sulfur-oxidizing *Gammaproteobacteria*. Red rectangle indicates *Beggiatoaceae* with investigated sequences of LSB are shown in bold. Nucleotide sequences were imported into ARB and aligned with the SINA aligner (Pruesse et al., 2012). The tree was calculated with RAxML implemented in ARB (1,533 position used), a 50% position variability filter and the GTR substitution matrix. Bootstraps were calculated using 100 resampling’s and bootstrap values are as shown in the legend. Scale bar represents 10% sequence divergence.

Experimental approaches with the genus *Thiomargarita* are still sparse due to the lack of marine LSB cultivars and no genomic data is currently available. While LSB ecological habitats are governed by similar conditions, *Thiomargarita* physiological differentiates itself from its relatives by the lack of motility (Salman et al., 2013). In this study, we analyzed the genome of a single cell of the candidate species “*Thiomargarita nelsonii*” collected from the sediments off the coast of Namibia. Members of “*Ca.* T. nelsonii” were recently described to display the most diverse range of morphologies within the LSB, i.e., single free-living spherical cells, unicells entrapped in empty diatom shells, or surrounded by an envelope structure, aggregates of reductively dividing, square-shaped cells, and attached living, elongated cells (Salman et al., 2013). The “*Thiomargarita nelsonii*” in this study was distinct from other morphotypes in that it forms distinctive, cylindrical shape cells often found in chains (**Figure 1A**).

We performed multiple displacement amplification (MDA) and genome sequencing of a single cell, “*Ca.* T. nelsonii Thio36”, and annotated the draft genome for comparison to six genomes of freshwater and marine *Beggiatoaceae*, including the unpublished genome *Beggiatoa* sp. strain ‘35Flor’ (Winkel et al., in preparation) that was isolated from black-band diseased corals (Brock and Schulz-Vogt, 2011). We analyzed major pathways involved in energy, sulfur, carbon, phosphorus, and nitrogen metabolism in “*Ca*. T. nelsonii Thio36”. As it has yet to be resolved whether *Thiomargarita* performs DNRA or denitrification, a special focus of this analysis was the nitrogen cycle.

## Materials and Methods

### Samples

Sediment containing *Thiomargarita* cells were taken within the mud belt of the Benguela Upwelling System off the coast of Namibia across the coordinate block 21°1.01 – 25°30.00 S and 12°13.75 –14°23.36 E during the cruise M76 onboard the R/V Meteor (April 12th – May 13th, 2008). Sediment was retrieved with a multicorer from water depths between 100 and 200 m across the coordinate block. The upper 3 cm of the sediments, which contained most of the *Thiomargarita* cells, were stored in closed plastic containers overlaid with bottom sea-water and kept at 4°C.

### Separation of Single Cells

Single cells with cylindrical shapes (**Figure 1A**) were separated from their external sheath under a stereo-microscope at a magnification of 20x. The sheath was opened with two sterile needles and single cells were manually peeled out. The cells were clearly visible by their sulfur inclusions. Cells were carefully washed in autoclaved, sterile-filtered, 0.4% low-melting agarose (Lonza NuSieve, Basel, Switzerland) that was dissolved in seawater. We used wide-bored pipet tips to avoid cell damage. This procedure was repeated several times to remove potentially contaminating microorganisms and/or exogenous DNA. Cells were transferred onto Ampligrid slides (Advalytix, Olympus, Hamburg, Germany) that contained a hydrophilic central area surrounded by a hydrophobic ring, which allows amplification reactions in very small volumes to minimize contamination. Slides with single *Thiomargarita* cells were air-dried and immediately processed or stored aseptically at -20°C.

### Single-cell MDA Reaction

Multiple displacement amplification reactions of single cells were prepared under a PCR hood irradiated with UV-light to avoid contaminations with free DNA, plasmids, or DNA from human skin or breath. All used materials and chemicals were UV-light irradiated with the exception of the polymerase/primer mix. MDA reactions were performed with the Illustra GenomePhi V2 DNA amplification kit (GE Healthcare, Buckinghamshire, UK) as described previously (Jogler et al., 2011). MDA products were further diluted 1:100 and 1:1000 and assessed for potential contaminants by amplifying the 16S rRNA gene with different primer combinations targeting most bacteria (Supplementary Table S1). Furthermore, to verify the presence of *Thiomargarita*-specific DNA, the partial intergenic transcribed spacer (ITS) region up/downstream between the 16S and 23S rRNA gene was amplified. For detailed PCR conditions see SI, and all used oligonucleotides are listed in Supplementary Table S1.

### Whole Genome Sequencing and Assembly

The amplified genomic DNA from the single “*Ca.* T. nelsonii Thio36” cell was sequenced at the Joint Genome Institute (Walnut Creek, CA, USA) using 2x 150 bp pair-end library on an Illumina HiSeq platform (Illumina Inc., San Diego, CA, USA) according to the manufacturer’s protocol (Bennett, 2004). For quality control we used a sequence matching tool DUK with a kmer hashing method against the JGI in house artifact database, the human genome and typical MDA contaminations such as *Delftia, Pseudomaonas*, and *Escherichia*. After quality control and removal of redundant reads, the remaining reads were assembled with a combination of the Velvet (Zerbino and Birney, 2008) and Allpaths (Gnerre et al., 2010) assemblers. We predicted genes on the assembled contigs using Prodigal (Hyatt et al., 2010) and ran a BlastP and Megan analysis for taxonomic assignments. Created GC histograms were analyzed due to their different taxonomic levels down to subspecies. Based on a 7 Mbp genome the estimated coverage was >200x. The assembled sequences have been deposited in the DDBJ/NCBI/EMBL databases (BioSample: SAMN04479841, accession no. LUTY00000000). The 16S rRNA gene sequence of the MDA product is deposited under the accession number KU747080.

### Gene Prediction, Annotation and Pathway Reconstruction of “*Ca*. T. nelsonii Thio36”

Gene calling was performed by Glimmer3 for complete genes (Delcher et al., 2007), and MetaGene for partial genes (Noguchi et al., 2006). Ribosomal RNA gene sequences were predicted with the RNAmmer 1.2 software (Lagesen et al., 2007), and transfer RNAs were identified with tRNAscan-SE (Lowe and Eddy, 1997). The annotation was performed by a refined version of the GenDB v2.2 system (Meyer et al., 2003) supplemented by the java-based comparative analysis and search tool JCoast version 1.7 (Richter et al., 2008). Predicted ORFs were further verified via similarity searches against sequence databases NCBI-nr, Swiss-Prot, KEGG, COG, genomesDB (releases May 2013), and the protein family databases Pfam (release 27), and Inter-Pro (release 42). Signal peptide predictions were verified by SignalP (Dyrløv Bendtsen et al., 2004), and transmembrane helix-analysis by TMHMM (Krogh et al., 2001). In parallel, predicted protein coding sequences were automatically annotated with MicHanThi (Quast, 2006). The MicHanThi software predicts gene functions based on similarity searches using the NCBI-nr (including Swiss-Prot) and InterPro database. Pathways were manually reconstructed by comparison to published pathways in the Kyoto Encyclopedia of Genes and Genomes (KEGG) (Kanehisa and Goto, 2000). Predicted genes and pathways were further compared to automated annotations of the IMG/ER (Markowitz et al., 2012) platform. The IMG Genome ID is 2236661048.

### Genome Completeness and Contamination Control

The completeness of genomes was estimated based on tRNA counts compared to complete or nearly complete genomes of *Beggiatoaceae*. The number of tRNAs were 44, 46, and 47 for *Beggiatoa alba* (3 contigs), *Thioploca ingrica* (1 contig) and *Beggiatoa leptomitiformis* D-402 (1 contig) (Fomenkov et al., 2015), respectively. A second estimation was based on 137 annotated single-copy genes that include all ribosomal proteins genes (Campbell et al., 2013). Testing for potential contaminants, we manually searched the dataset for additional tRNAs and 31 single-copy genes (Ciccarelli et al., 2006) using BLAST.

### Phylogenetic Analysis

The nearly full length 16S rRNA gene sequence (1,392 bp) was used for tree calculation with the ARB software package (Ludwig et al., 2004), where trees were calculated based on the database of the ARB-Silva release 111 (Quast et al., 2012) using a maximum likelihood algorithm (RaxML) and a 50% base frequency filter. Subsequently, partial sequences were added to the reconstructed tree by the maximum parsimony algorithm without allowing changes in the overall tree topology. A multiple protein alignment of the ribulose-1,5-bisphosphate carboxylase/oxygenase large subunit form 1Aq (*rbcL*) was constructed with the integrated aligner of the ARB software tool, which was then manually refined. Phylogenetic tree reconstructions were performed with a maximum likelihood algorithm using the Dayhoff amino acid substitution matrix (Dayhoff et al., 1978) for evolutionary distance. A 75% base frequency and termini filter were applied considering 256 amino acid positions.

## Results and Discussion

### General Single-Cell Genome Features

We successfully amplified and sequenced the draft genome of an individual *Thiomargarita* cell, allowing us to study the genomic potential of this uncultured bacterium. Mapping of raw reads against an artifact database, the human genome and typical MDA contaminants removed 1.7% of the reads. The reads assembled to 3,613 contigs and 5.3 Mb of unique sequence information. The contig length ranged between 14.8 kbp and 504 bp with a N50 value of 1,835 bp. BlastP and Megan analysis showed highest similarity with *Beggiatoa* spp.. Based on the recovery of 25 tRNAs we estimated the genome completeness to be around 50–53% when compared to tRNAs numbers in genomes of *Beggiatoa alba* (46 tRNAs, 3 contigs), *Beggiatoa leptomitiformis* D-402 (47 tRNAs, 1 contig) and *Thioploca ingrica* (44 tRNAs, 1 contig). We identified 96 out of annotated 137 single-copy genes (Campbell et al., 2013) suggesting an even higher genome completeness of 70%. The details of the “*Ca*. T. nelsonii Thio36” draft genome as well as those for other genomic datasets in our comparative analysis are listed in **Table 1.**

**Table 1:** General genome feature of the investigated LSB.

genome feature	“*Ca.* Thiomargarita nelsonii Thio36”	“*Ca.* Thiomargarita nelsonii Bud S10”	“*Ca.* Isobeggiatoa divolgata”	“*Ca.* Maribeggiatoa sp.”	*Beggiatoa* sp. 35Flor	*Beggiatoa alba* B18LD	*Thioploca ingrica*
nucleotides	5.3 Mb	6.2 Mb	7.6 Mb	4.8 Mb	4 Mb	4.3 Mb	4.8 Mb
contigs	3,613	439	6,769	822	291	3	1
ORF	7,596^∗^	7,525	6,686	5,258	3,552	3,665	3,964
coding percentage	72	82	57	85	87	86	86
max. contig length	14 kb	190 kb	19 kb	71 kb	138 kb	500 kb	4.8 Mb
tRNAs	23	46	45	46	38	46	44
GC content [%]	42	41.3	38.5	38.2	38.5	40	41.2
proteins of known function	3,486	4,310	3,414	2,962	2,746	2,867	2,788
conserved hypothetical proteins	967	n.d.	1046	619	380	377	n.d.
hypothetical proteins	3143	n.d.	2226	1677	426	421	n.d.
genome completeness (%) based on 137 SCG^†^	70	89.8	98.5	98.5	97.8	100	100
reference	this study	Flood et al., 2016	Mußmann et al., 2007	MacGregor et al., 2013b	IMG Genome ID 2606217769	BioProject PRJNA224116	Kojima et al., 2015


*^∗^Likely overestimation due to high number of frame shifts. n.d., not determined. ^†^Based on 137 annotated single-copy genes (SCG) (Campbell et al., 2013).*

### Screening for Contaminating DNA

We confirmed the purity of the genome by PCR screening of 16S rRNA genes, as well as *in silico* examining genes only found in single copies using blastn and blastp analyses. *Thiomargarita* spp. have up to four self-splicing introns in their 16S rRNA gene (Salman et al., 2012), which facilitated the discrimination of the “*Ca*. T. nelsonii Thio36” 16S rRNA gene amplicons from potentially amplified contaminating DNA, as the elongated 16S rRNA gene would be discriminated against during universal PCR amplification (Salman et al., 2012). Thus, prior to genome sequencing of the single-cell MDA product, we performed a PCR using universal bacterial 16S rRNA primers (Supplementary Table S1), and retrieved a single product of the expected size of ∼2,300 bp (Salman et al., 2012). The 16S rRNA gene of the single-cell genome (1,392 bp, without the intron of 128 bp) clustered phylogenetically with other 16S rRNA sequences of “*Ca*. T. nelsonii”, sharing 99.1–100% sequence identity with this species cluster (**Figure 1B**). The recovery of 31 single-copy genes including ribosomal proteins and others, plus *SecG, HrcA, RimM*, and PNPase showed highest similarity to “*Ca.* T. nelsonii Bud S10” (Supplementary Table S2) and other *Beggiatoaceae.* Consistent with this, 4,062 ORFs (of a total of 7,596) displayed best BLAST hits to *Gammaproteobacteria*. All our results point at a contamination-free amplified genome of “*Ca.* T. nelsonii Thio36”.

### Phylogenetic Affiliation

The genus *Thiomargarita* belongs to the *Beggiatoaceae*, a family within the *Gammaproteobacteria*, and encompasses three species, of which two have only recently been proposed as *Candidatus* species (Salman et al., 2011). In the genome of “*Ca*. T. nelsonii Thio36”, we detected a nearly complete 16S rRNA gene that contained an intron (S1369) at the expected position for “*Ca.* T. nelsonii” (Salman et al., 2012). The 16S rRNA gene without intron sequence was used for phylogenetic tree reconstructions, and affiliated with 99.1–100% nucleotide identity to the cluster of other “*Ca.* T. nelsonii” sequences including other chain-forming morphotypes (Salman et al., 2011; **Figure 1B**).

To further analyze the relatedness to other LSB we compared the “*Ca.* T. nelsonii Thio36” dataset with the draft genome of “*Ca.* I. divolgata” (Mußmann et al., 2007) and tested for reciprocal best match (RBM) hits. The two genomes shared 471 open reading frames (ORFs) (cut off e^-05^, 65% alignment coverage). Usually, the expected number of RBM hits among genomes of different genera of a family is significantly higher, however, here the considerably lower numbers might be caused by the fact that a large amount of genes are only partially presented in the draft genome datasets.

### Genes of Potentially Cyanobacterial Origin

A total of 626 genes had best blastp hits to cyanobacterial genes, which is consistent with previous studies of LSB genomes (Mußmann et al., 2007; MacGregor et al., 2013c; Flood et al., 2014). For example, the ‘*Ca.* T. nelsonii Thio36’ genome encodes *xisH* and *xisI* genes (Supplementary Table S3), which are required in cyanobacteria for the heterocyst-specific rearrangement of the FdxN element as part of the nitrogenase operon (Ramaswamy et al., 1997). The FdxN element is also encoded in the genomes of ‘*Ca.* T. nelsonii Thio36’, ‘*Ca.* Maribeggiatoa sp.’, and ‘*Ca.* I. divolgata’ (data not shown). Almost all genes with sequence identities higher than 70% were flanked by *Beggiatoaceae* genes, while the GC content varies between 30.1 and 55.4% (Supplementary Table S3), all of which further indicates a substantial horizontal gene transfer between ancestors of LSB and cyanobacteria (Mußmann et al., 2007; MacGregor et al., 2013c).

### Nitrogen Metabolism

**FIGURE 2:**
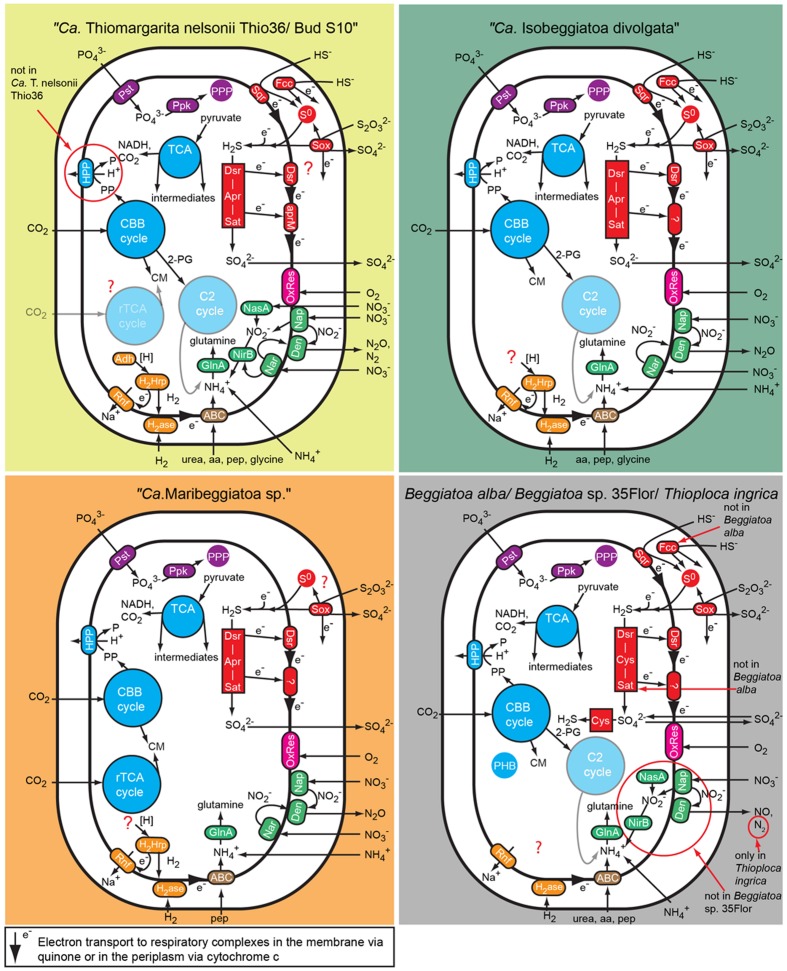
**Comparison of C, N, S, P, and energy pathways in the investigated large, colorless LSB based on the genomic information.** Note that not all genes essential for some of the predicted pathways have been found in the genomes because of the fragmented nature of most genomes. Aa, amino acid; ABC, ABC transporter; Adh, alcohol dehydrogenase; Apr, APS reductase; CBB cycle, Calvin-Benson-Basshman cycle; C2 cycle, glyoxylate cycle; CM, cell material; Cys, 3″ phosphoadenylylsulfate reductase; Den, denitrification proteins; Dsr, dissimilatory sulfite reductase and related proteins; Fcc, flavocyctochrome c; GlnA, Glutamine synthetase; H_2_ase, uptake hydrogenase; H_2_Hrp, methyl viologen-reducing hydrogenase:heterodisulfide reductase complex; HPP, proton translocating pyrophosphatase; Nar, membrane-bound respiratory nitrate reductase; Nap, periplasmatic respiratory nitrate reductase; NasA, assimilatory nitrate reductase; NirB, assimilatory and dissimilatory nitrite reductase; OxRes, oxygen respiration; pep, peptides; PHB, polyhydroxybutyrate granule; PPP, polyphosphate granule; Ppk, polyphosphate kinase; Pst, phosphate transport system; Rnf, membrane-bound electron transport complex; rTCA, reductive tricarboxylic acid cycle; S^0^, sulfur globules; Sat, ATP sulfurylase; Sox, SOX enzyme complex; Sqr, sulfide quinone reductase; TCA, tricarboxylic acid cycle.

The “*Ca.* T. nelsonii Thio36” genome encodes genes for the reduction of nitrate via DNRA and denitrification pathway. We identified two dissimilatory nitrate reductases (*nar* and *nap*), and an assimilatory nitrate reductase (*nasA*) (**Figure 2** and Supplementary Table S4). The membrane-bound (*nar*) catalyzes the first step of nitrate-reduction in the denitrification pathway, while the periplasmic (*nap*) dissimilatory nitrate reductase catalyzes the same reaction for the denitrification and DNRA pathway (Moreno-Vivián et al., 1999; Morozkina and Zvyagilskaya, 2007). Both dissimilatory nitrate reductase genes are also present in “*Ca.* I. divolgata” (Mußmann et al., 2007), in “*Ca*. T. nelsonii Bud S10” (Flood et al., 2016) as well as in “*Ca.* Maribeggiatoa sp.” (MacGregor et al., 2013b), whereas the genome of *Beggiatoa alba* B18LD only encodes the periplasmic enzyme (**Figure 2** and Supplementary Table S4). The cytoplasmic assimilatory nitrate reductase (*nasA*) is also found in *Beggiatoa alba* B18LD, *Thioploca ingrica*, and “*Ca*. T. nelsonii Bud S10” together with a nitrite reductase (*nirBD*) involved in both the assimilatory and dissimilatory pathway. In combination with the assimilatory nitrate reductase (*nasA*), these two enzymes assimilate ammonium (**Figure 2**; **Table 2**), as was shown for endosymbiontic SOB of the vesicomyid clams and *Bathymodiolus* sp. mussels (Kleiner et al., 2012a). In “*Ca.* T. nelsonii Thio36”, “*Ca.* T. nelsonii Bud S10”, *T. ingrica* and *B. alba* B18LD, the nitrite reductase could be used to respire nitrite via DNRA (**Figure 2**; **Table 2** and Supplementary Table S4), which has been experimentally demonstrated for *B. alba* (Vargas and Strohl, 1985). In contrast, the genomes of marine *Beggiatoa* lack both *nasA* and *nirBD*. Instead, they encode a multiheme cytochrome with nitrite reductase function (MacGregor et al., 2013b), which may be involved in DNRA (Klotz et al., 2008; Hawley et al., 2014; Jones et al., 2015). For a more detailed description see SI.

**Table 2:** Energy conservation and metabolic pathways in the investigated LSB.

Large colorless sulfur-oxidizing bacteria (SOB)							
Pathways	“*Ca.* Thiomargarita nelsonii Thio36”	“*Ca.* Thiomargarita nelsonii Bud S10”	“*Ca*. Isobeggiatoa divolgata”	“*Ca*. Maribeggiatoa sp.”	*Beggiatoa* sp. 35Flor	*Beggiatoa alba* B18LD	*Thioploca ingrica*
sulfide oxidation	+	+	+	+	+	+	+
elemental sulfur oxidation via reverse DSR pathway	+	?	+	+	+	?	+
sulfite oxidation	+	+	+	+	+	-	+
assimilatory sulfate reduction	?	?	-	-	+	+	+
thiosulfate oxidation via SOX pathway	+	+	+	+	+	+	+
dissimilatory nitrate reduction to ammonium	+	+	- (potential via multiheme protein?)	- (potential via multiheme protein?)	-	+	-
assimilatory nitrate reduction to ammonium	+	+	-	-	-	+	+
denitrification	+	+	+	+	-	+	+
glycolysis	+	+	+	+	+	+	+
tricarboxylic acid cycle	+	+	+	+	+	+	+
glyoxylate bypass	-	-	-	-	+	+	+
polyhydroxybutyrate syntheses	-	-	-	-	+	+	+
carbon fixation via CBB-cycle	+	+	+	+	+	+	+
carbon fixation via reductive tricarboxylic acid cycle/acetly CoA reduction	+/+	?/-	?/-	+/-	-/-	-/-	-/-
C2-cycle (glycolate cycle)	+	+	+	-	+	+	+
oxidative phosphorylation	+	+	+	+	+	+	+
flavin-based energy bifurcation	+	n.a.	+	+	-	-	n.a.
potential hydrogen oxidation	+	?	+	+	+	+	?
Na^+^ -translocating membrane complex	+	+	+	+	+	+	+


*+ complete set of genes for metabolic pathway encoded. ? no complete set of genes for pathway or pathway with alternative genes. - genes of pathway not found or not encoded. n.a., not analyzed.*

Genes encoding the nitrite reductase (*nirSCF*) for denitrification are also present in “*Ca*. T. nelsonii Thio36”, “*Ca*. T. nelsonii Bud S10”, *T. ingrica* and “*Ca*. *I. divolgata*”. Furthermore, “*Ca.* T. nelsonii Thio36”, “*Ca*. T. nelsonii Bud S10”, *T. ingrica*, “*Ca.* I. divolgata”, and “*Ca.* Maribeggiatoa sp.” encode a membrane-bound nitric oxide reductase (*nor*). Together with the earlier described periplasmic nitrate reductase and the multiheme cytochrome with nitrite reductase-function, all five organisms have the genetic potential to denitrify nitrate to nitrous oxide (**Figure 2** and Supplementary Table S4). Interestingly, we also found genes for the nitrous oxide reductase (*nosZD*) (Supplementary Table S4) in “*Ca*. T. nelsonii Thio36”, “*Ca*. T. nelsonii Bud S10”, and *T. ingrica*. Accordingly, a completely functioning denitrification pathway to dinitrogen in all these organisms is possible (**Figure 2** and Supplementary Table S4). Thus, “*Ca.* T. nelsonii Thio36” may gain energy via both nitrate reduction pathways, DNRA and denitrification. We speculate that the alternate use of these two pathways is determined by the fluctuating redox conditions in the highly dynamic habitat of *Thiomargarita*. Thus, a differential expression pattern may be controlled by external environmental conditions as shown before (Kraft et al., 2014), realized here within one cell instead of within a whole community. For a detailed description of the genes involved in nitrogen metabolism see supplementary information.

The draft genome of strain *Beggiatoa* sp. 35Flor lacks all necessary genes for DNRA and denitrification, which is in line with experimental data. This strain rather uses internally stored sulfur for anaerobic respiration (Schwedt et al., 2012).

### Carbon Metabolism

#### Glycolysis

The “*Ca*. T. nelsonii Thio36” genome encodes a nearly complete glycolysis pathway, while it lacks a glucose-6-phosphate isomerase, a trait shared with the “*Ca*. T. nelsonii Bud S10” genome (Supplementary Table S4). This gene was found in all other LSB, therefore it might either be encoded in the missing section of the two “*Ca*. T. nelsonii” genomes, or represent a true deletion. “*Ca*. T. nelsonii Thio36” encodes a polyphosphate glucokinase instead of the ATP glucokinase, which is typical of microorganisms that accumulate polyphosphate (Tanaka et al., 2003). Thus, there is genetic evidence for the observation that *T. namibiensis* accumulates polyphosphate granules (Schulz and Schulz, 2005). All other LSB contain a complete glycolysis pathway with “*Ca.* I. divolgata”, *T. ingrica* and “*Ca.* Maribeggiatoa sp.” also processing polyphosphate-dependent glucokinase. So far, cultured freshwater LSB have not been shown to grow on sugars and other complex organic substrates, but they can use acetate as a carbon and energy source (Teske and Salman, 2014).

#### Tricarboxylic Acid Cycle (TCA) and Glyoxylate Bypass

“*Ca*. T. nelsonii Thio36” encodes a complete tricarboxylic acid cycle (TCA) cycle, consistent with most other LSB (**Table 2** and Supplementary Table S4). A complete TCA cycle is not common among lithotrophic organisms, such as sulfur oxidizers, because they are often obligate autotrophs (Teske and Salman, 2014). In particular, the presence of a complete alpha ketoglutarate dehydrogenase-complex is unusual, as the lack of this complex has been used as an indicator for obligate autotrophy (Wood et al., 2004).

The glyoxylate-cycle uses five enzymes of the TCA cycle (citrate synthase, aconitase, succinate dehydrogenase, fumarase, malate dehydrogenase) to form succinate and glyoxylate from isocitrate for gluconeogenesis in organisms growing on 1- or 2-C compounds such as acetate. The essential genes encoding malate synthase and isocitrate lyase (Walsh and Koshland, 1984) were so far only detected in the genomes of the marine *Beggiatoa* sp. strain 35Flor and in the freshwater LSB *T. ingrica* and *B. alba* B18LD (**Table 2** and Supplementary Table S4). This confirmed earlier experimental studies suggestive of a functional glyoxylate bypass with the freshwater *Beggiatoa* sp. strains OH-75-B (Nelson and Castenholz, 1981) and D-402 (Stepanova et al., 2002). Moreover, all LSB with the exception of “*Ca.* Maribeggiatoa sp.” encode an acetyl-CoA synthethase to activate acetate, which may serve as a potential energy and carbon source. This is in line with the proposed mixotrophic lifestyle of *Thiomargarita namibiensis* (Schulz and de Beer, 2002; Beutler et al., 2012) based on the observation that it may use organic carbon such as acetate as carbon source, but not as energy source. Accordingly, the reverse TCA (rTCA) could be utilized by *Thiomargarita* spp. to transform inorganic carbon into biomass, as is discussed below.

### Carbon Fixation Pathways

#### Calvin–Benson–Bassham (CBB) Cycle

**FIGURE 3:**
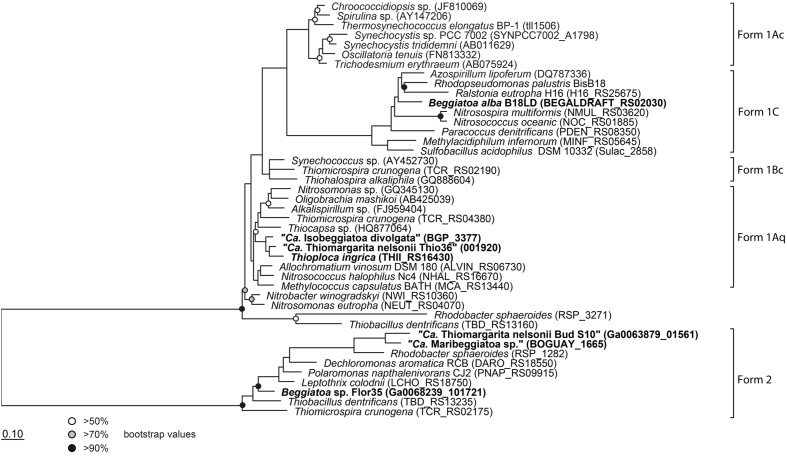
**Phylogenetic tree of the large subunit of the ribulose-1,5-bisphosphate carboxylase/oxygenase (RubisCO).** All investigated LSB contain the gene and are shown in bold. Protein sequences were imported into ARB and aligned with the integrated aligner. The tree was calculated with RAxML implemented in ARB (256 positions used) and bootstraps were calculated using 100 resampling’s. Scale bar represents 10% sequence divergence.

The key enzyme of carbon fixation via the CBB-cycle is the ribulose-1,6-bisphosphate carboxylase/oxygenase (RuBiscCO), which was initially detected in a marine *Beggiatoa* sp. (Nelson and Jannasch, 1983). The genomes of “*Ca*. T. nelsonii Thio36”, “*Ca.* I. divolgata”, *T. ingrica* and *B. alba* B18LD encode RuBisCO form I, while “*Ca.* Maribeggiatoa sp.”, “*Ca.* T. nelsonii Bud S10” and *Beggiatoa* sp. strain 35Flor encode RuBisCO form II (**Figure 3** and Supplementary Table S4). It is known that other SOB express different forms of this enzyme (Kleiner et al., 2012a) with distinct affinities for CO_2_ and O_2_ (Badger and Bek, 2008). We also identified most genes of the CBB-cycle in “*Ca*. T. nelsonii Thio36” with the exception of the sedoheptulase-1,7-bisphophatase and the fructose-1,6-bisphosphatase. Instead, a 6-phosphofructokinase was present (Supplementary Table S4), which contains pyrophosphate-binding sites similar to a pyrophosphate-dependent 6-phosphofructokinase (Reshetnikov et al., 2008). This enzyme is proposed to have a tri-function replacing the missing enzymes sedoheptulase-1,7-bisphophatase, the fructose-1,6-bisphosphatase as well as a phosphoribulose kinase (Kleiner et al., 2012b). A possible involvement in additional carbon fixation was proposed (see Supplementary Information). The genome of “*Ca*. T. nelsonii Thio36” also lacks a ribose-5-phosphate isomerase, which is likely due to the incompleteness of the assembled genome. This enzyme is encoded in all other LSB (Supplementary Table S4).

It is interesting that although “*Ca*. T. nelsonii Thio36” and “Bud S10” belong to the same *Thiomargarita* species they contain two different types of RubisCO enzymes. The two strains thrive in extremely different habitats, which could be a determining factor for this different adaptation. “*Ca*. T. nelsonii Thio36” lives in coastal sediments influenced by the decay of diatom-rich phytodetritus from surface waters that are dependent on the upwelling of nutrient-rich bottom waters. Here, upwelling intensity, and thus phytodetrial flux to the sediments, is seasonal and dissolved CO_2_ may be less bioavailable under periods of low nutrient input promoting a need for a more discriminatory RuBisCO type I. On the contrary, “*Ca*. T. nelsonii Bud S10” lives in dense microbial mats in deep-sea sediments above a cold seep system. These sediments are strongly influenced by constantly advecting methane-rich fluids that stimulate high rates of anaerobic methane oxidation and respiration of sulfate (Treude et al., 2003). Under these conditions, CO_2_ generated via respiration is likely to be high while oxygen levels consistently lower, thus favoring a form II RuBisCO.

#### Oxygenase-Activity of RuBisCO and a Potential Glycolate Cycle

**FIGURE 4:**
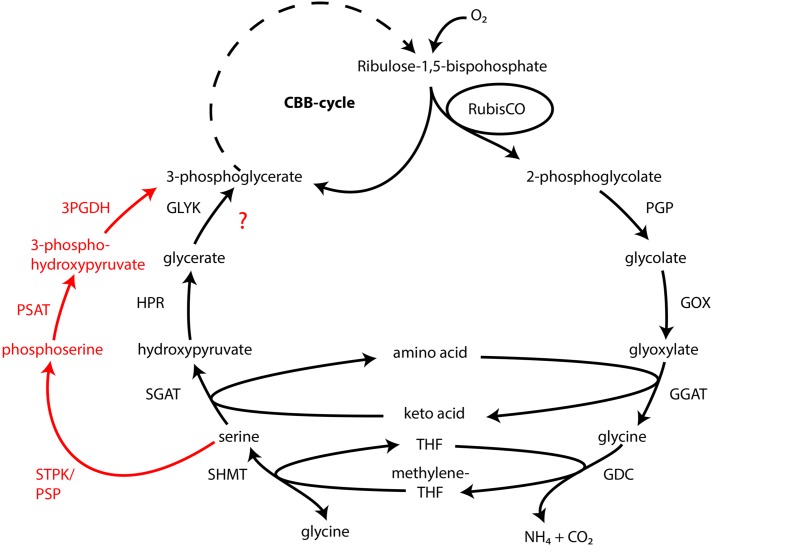
**Glycolate cycle of the investigated LSB with alternative phosphoserine shuttle of “*Candidatus* Thiomargarita nelsonii” and “*Candidatus* Isobeggiatoa divolgata” (red).** PGP, phospoglycolate phosphatase; GOX, glycolate oxidase; GGAT, glutamate:glyoxylate aminotransferase; GDC, glycine decarboxylase; SHMT, serine hydroxymethyltransferase; SGAT, serine:glyoxlyate aminotransferase; HDR, hydroxypyruvate reductase; GLYK, glycerate kinase type III; STPK, serine threonine protein kinase; PSAT, phosphoserine aminotransferase; 3PGDH, 3-phosphoglycerate dehydrogenase.

Atmospheric oxygen concentrations stimulates growth in *Thiomargarita* spp. (Schulz, 2006), and the production of the 2-phosphoglycolate (2-PG) by the oxygenase activity of RuBisCO can be expected. Since 2-PG is an inhibitor of the CBB-cycle it needs to be removed for a proper functioning of the CBB cycle. In higher plants this is carried out by the photorespiratory metabolism (Bauwe et al., 2012), which converts the 2-PG into 3-phosphoglycerate (3-PG) and shuttles it back into the CBB cycle. An active 2-PG metabolism (C2-cycle) that converts 2-PG and shuffles it back into the Calvin-cycle has been demonstrated for the unicellular cyanobacterium *Synechocystis* sp. strain PCC 6803 (Eisenhut et al., 2008). Other earlier observations in the serine biosynthesis pathway of the methanotrophic *Methylococcus capsulatus* (Bath) proposed a similar pathway to the C2-cycle of higher plants (Taylor et al., 1981; Ward et al., 2004). The role for such a C2-cyle in microorganisms using RuBisCO for carbon fixation is not clear. Nevertheless, the genome of “*Ca*. T. nelsonii Thio36” encodes an almost complete set of genes that are involved in the C2-cycle (Supplementary Table S4). While the only missing gene is the essential class-III glycerate kinase (GLYK) producing 3-PG, however, we found genes that can convert the photorespiratory intermediate serine via a phosphorylation to phosphoserine and produce 3-PG in an alternative way (Supplementary Table S4; **Figure 4**). Homologs of the C2 cycle genes were also detected in other LSB, with the exception of “*Ca.* Maribeggiatoa sp.”, which lacks a glycolate oxidase (**Figure 4** and Supplementary Table S4). For a detailed description see Supplementary Information.

#### Reductive Tricarboxylic Acid (rTCA)

Until now, only the sulfur-oxidizing endosymbiont “*Ca.* Endoriftia persephone” of the hydrothermal tubeworm *Riftia pachyptila* has been shown to encode both the CBB- and the rTCA-cycle for CO_2_ fixation, and expresses them under different conditions (Markert et al., 2007). Besides the CBB-cycle we also found genes encoding a potentially complete rTCA in the genome of “*Ca*. T. nelsonii Thio36” (Supplementary Table S4). We found a gene that showed >70% sequence identity to the ATP-citrate lyase of “*Ca.* E. persephone” alpha subunit, which is a key enzyme for the rTCA. This gene is also present in the genome of “*Ca.* Maribeggiatoa sp.” (MacGregor et al., 2013b), but the beta subunit of the ATP-citrate lyase cannot be found, so it is not clear if the enzyme is functional. Nevertheless, we found other genes indicative for the rTCA, such as 2-oxoglutarate:ferredoxin oxidoreductase (*korAB*), and the fumarate reductase large subunit (*frdA*). These genes are present in the genomes of “*Ca.* T. nelsonii Thio36”, “*Ca*. T. nelsonii Bud S10”, “*Ca.* I. divolgata”, and “*Ca.* Maribeggiatoa sp.”. “*Ca.* T. nelsonii Thio36” may further convert the produced acetyl-CoA through the ATP citrate lyase to fix two more molecules of CO_2_ (Evans et al., 1966) (Supplementary Table S4). The encoded enzymes for this pathway are pyruvate:ferredoxin oxidoreductase (*porABGD*), phosphoenolpyruvate synthase (*ppsA*), and ATP-dependent phosphenolpyruvate carboxykinase (*pckA*). Accordingly, “*Ca*. T. nelsonii Thio36” and “*Ca.* Maribeggiatoa sp.” might be additional examples of SOB that are capable of fixing carbon by two distinct CO_2_ fixation pathways, but this interpretation should be confirmed through additional experiments.

### Energy Metabolism

#### Sulfur and Hydrogen Oxidation

We found only a subset of genes known to be encoded by LSB genomes necessary for the metabolism of reduced sulfur compounds (**Table 2**). Likely, this is the result of the incomplete nature of the genome. The sulfide:quinone oxidoreductase (*sqr*) is present in all genomes, while an alternative enzyme, the flavocytochrome c sulfide dehydrogenase (*fccAB*), is also encoded with both subunits in all genomes with the exception of *B. alba* B18LD (**Figure 2**; Supplementary Table S4). The occurrence of two different enzymes for the same substrate, sulfide, points toward tightly regulated sulfide oxidation under different environmental conditions in these organisms. LSB oxidize sulfide to sulfur that is stored in the periplasm by a yet unknown process (Dahl et al., 2008), and is visible as bright white globules in all analyzed LSB (**Figure 1A**) (Mezzino et al., 1984; Mußmann et al., 2007; Salman et al., 2011; Schwedt et al., 2012; MacGregor et al., 2013b).

We also found an incomplete set of genes for the reverse dissimilatory sulfite reductase (rDSR) pathway, and the thiosulfate-oxidizing Sox-pathway. The set of genes for these pathways are nearly complete in the genomes of “*Ca.* I. divolgata”, *T. ingrica, Beggiatoa* sp. 35Flor, “*Ca.* Maribeggiatoa sp.”, and “*Ca.* T. nelsonii Bud S10” (**Figure 2** and Supplementary Table S4). For a more detailed description of the localization of genes involved in the sulfur cycle see Supplementary Information.

Recently, an aerobic-type, uptake [Ni,Fe] hydrogenase was found actively oxidizing hydrogen in the endosymbiontic SOB of the hydrothermal vent mussel *Bathymodiolus* (Petersen et al., 2011). The same type of hydrogenase is encoded by “*Ca.* T. nelsonii Thio36” and almost all other LSB genomes. For detailed analysis of hydrogenase genes in LSB see (Kreutzmann, 2013). Accordingly, the potential for hydrogen oxidation seems to be more widespread among SOB than previously expected. While hydrogen has been showed to serve as an alternative energy source in the *Beggiatoa* sp. strain 35Flor (Kreutzmann and Schulz-Vogt, 2016), it has yet to be tested in other LSB.

#### Oxidative Phosphorylation

Non-photosynthetic SOB usually couple the oxidation of inorganic sulfur compounds or organic carbon (e.g., acetate) to the transport of electrons during oxidative phosphorylation to produce a proton motive force for energy production in the form of ATP. Oxidative phosphorylation has been demonstrated experimentally for freshwater and marine *Beggiatoa* strains (Strohl et al., 1986; Prince et al., 1988).

Genes for all five complexes of the respiratory electron transport chain are present in the genome of “*Ca.* T. nelsonii Thio36” and all other LSB (**Table 2**; Supplementary Table S4). All genomes encoded the NADH dehydrogenase I (*nouABCDEFGHIJKLMN*), but the genes for the subunits were typically scattered over several operons due to the fragmented nature of most datasets. “*Ca*. T. nelsonii Thio36”, lacks the genes *nouABC*, and *Beggiatoa* sp. strain 35Flor lacks the gene *nouE* (Supplementary Table S4). The membrane-associated succinate dehydrogenase complex II (*sdhABCD*) is present in all genomes, while homologs for the membrane anchor (*sdhD*) cannot be detected in “*Ca.* T. nelsonii Thio36”, *T. ingrica* and “*Ca.* Maribeggiatoa sp.”. The third complex is encoded by genes for the ubiquinol-cytochrome c reductase, and a similar operon (*petABC*) is present in all genomes. Only in “*Ca. I. divolgata*”, the *petC* gene is located on a different contig than the *petAB* genes (Supplementary Table S4). Several different complex IV-associated genes were found in the genomes. While the cytochrome c oxidase cbb3-type (*ccoNOQP*, high oxygen affinity) is present in all genomes (Supplementary Table S4), we only detected the cytochrome c oxidase aa3-type (*coxCBA*, low oxygen affinity) in the genomes of “*Ca.* T. nelsonii Thio36” and “*Ca.* I. divolgata” (Supplementary Table S4). This was unexpected, because the activity of a cytochrome c oxidase aa3-type has been reported for the freshwater strain *Beggiatoa leptomitiformis* D-402 (Muntyan et al., 2005). In the genomes of “*Ca*. T. nelsonii Bud S10 and Thio36”, *B. alba* B18LD and “*Ca.* Maribeggiatoa sp.” we found a third complex, a cytochrome d ubiquinol oxidase (*cydAB*) (Supplementary Table S4). All genomes encode the F-type ATPase (*atpCDGAHFEB*), while we find homologs of vacuolar (V)-type ATPase (*ntpABCDEFGHIK*) only in the marine LSB (Supplementary Table S4). The F-type ATPase is involved in ATP synthesis at the cytoplasmic membrane, while the V-type ATPase is used to hydrolyse ATP at the vacuolar membrane to facilitate ion-translocations (Mußmann et al., 2007; Beutler et al., 2012). The latter may be a specific feature for the large vacuolated marine SOB of the family *Beggiatoaceae*.

#### Heterodisulfide and Flavin-based Electron Bifurcation

Homologous genes for a heterodisulfide reductase (*hdrABC*) involved in energy metabolism have been intensely studied in methanogens (Thauer et al., 2008) and sulfate-reducing *Deltaproteobacteria* (Haveman et al., 2003; Strittmatter et al., 2009), and were found in “*Ca.* T. nelsonii Thio36”. Methanogens use Hdr in a complex with a coupled methyl viologen-reducing hydrogenase (Mvh) to reduce a coenzyme during methanogenesis (Thauer et al., 2008). We identified a homologous Mvh in “*Ca*. T. nelsonii Thio36”, but it is organized in a different operon, suggesting a decoupling of the gene functions. The role of such a decoupled complex in SOB is unclear. But there is evidence that the heterodisulfide reductase is involved in sulfur oxidation, since it was up-regulated in the proteome of the sulfur-oxidizing endosymbiont “*Ca.* Endoriftia persephone” under sulfur-rich conditions (Markert et al., 2007). Interestingly, both genes had best blast hits to *Geobacter* spp. with identities between 34 to 48%, and occur in a similar operon (Supplementary Figures S1 and S2). The heterodisulfide reductase function in *Geobacter* spp. is so far not known (Coppi, 2005).

“*Ca.* T. nelsonii Thio36” encodes other heterodisulfide reductase genes (*hdrDEF*). *HdrDE* genes occur in methanogens with cytochromes, where these membrane-bound enzymes, together with a hydrogenase (*vhoACG*), reduce the CoM-S-S-CoB complex, similar to methanogens without cytochromes (Thauer et al., 2008). It was unusual that neither of the two enzymes contained transmembrane domains, but both had two typical iron-sulfur binding sites (CX_2_CX_2_CX_3_C) and the *hdrE* heme binding site (CX_2_CH). The function of the *hdrF* gene, which encodes a heterodisulfide oxidoreductase with a NAD(P)H subunit is unclear. The same sequence and operon structure of the *hdrDEF* genes was also found in the genome of “*Ca.* Maribeggiatoa sp.” and “*Ca*. T. nelsonii Bud S10”, while upstream of the complex we found a methyl viologen-reducing hydrogenase delta subunit (*mvhD*) together with two genes that code for the heterodisulfide reductase alpha subunit (*hdrA*) (Supplementary Figure S1 and Supplementary Table S4). Interestingly, upstream of the *hdrA* genes is a gene encoding a fumarate reductase/succinate dehydrogenase (*fdrA*/*sdhA*), which might be a coupling to membrane-bound electron transport. The latter combination is also present in the genome of “*Ca.* T. nelsonii Thio36” (Supplementary Figure S1).

**FIGURE 5:**
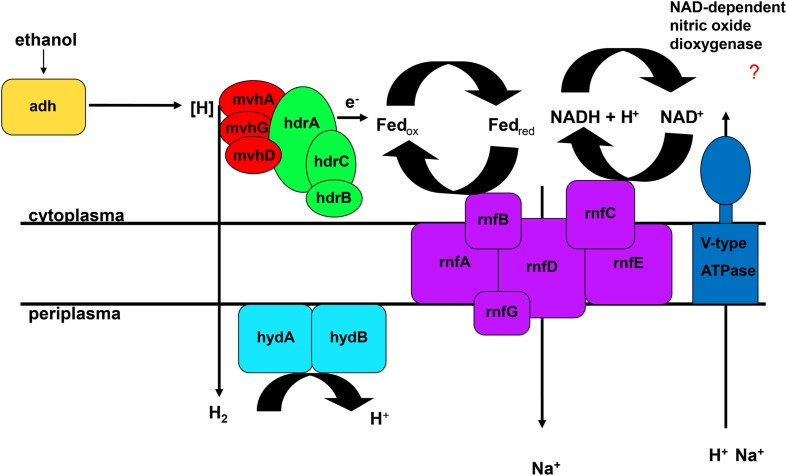
**Flavin-based electron bifurcation coupled to ethanol oxidation.** Adh, alcohol dehydrogenase; hdr, heterodisulfide reductase; hyd, periplasmatic hydrogenase; mvh, methyl viologen-reducing hydrogenase; rnf, membrane-bound electron transport complex.

The FAD-containing iron-sulfur protein *hdrA* is of special interest. It uses ferredoxin as electron acceptor and can be coupled to a ferredoxin-oxidizing Na^+^-translocating membrane complex in a so called flavin-based electron bifurcation (FBEB) pathway (Buckel and Thauer, 2013). Strikingly, all genomes of the large, colorless SOB have genes for a full Na^+^-translocating RnfABCDGE membrane complex that oxidizes ferredoxin and reduces NAD^+^ (Biegel et al., 2011) (**Figure 5**; **Table 2** and Supplementary Table S4). Therefore, a FBEB process could be used by them and “*Ca.* T. nelsonii Thio36” under long-term anoxic conditions in sulfidic waters or during migration of motile LSB such as “*Ca.* I. divolgata” and “*Ca.* Maribeggiatoa sp.” into deeper anoxic sediment layers at times when their internal electron acceptor nitrate is used up.

The genome of “*Ca.* T. nelsonii Thio36” also contains other periplasmic hydrogenases involved in energy metabolism. The periplasmic-located nickel-dependent hydrogenase (*hydAB*) was found in SRB and is up-regulated during ethanol oxidation in *Desulfovibrio vulgaris* (Haveman et al., 2003). In SRB they are proposed to be involved in hydrogen cycling between the cytoplasm and periplasm, whereby the alcohol dehydrogenase (*adh*) transfers hydrogen to the Hdr:Mvh-complex which produces molecular hydrogen. This is further transferred directly to the periplasmic hydrogenase *hydAB* by a yet unidentified membrane complex, converting it back to protons and producing a proton motive force (Haveman et al., 2003). Thus, the coupling of FBEB and hydrogen cycling would result in the higher proton/Na^+^ ion motive force (**Figure 5**). Interestingly, we also found a gene encoding an *adh* in the genome of “*Ca*. T. nelsonii Thio36”, thus alcohol oxidation coupled to FBEB might be possible. Homologs for both *hydAB* and *adh* genes are not found in any other genome of the LSB (Supplementary Table S4). So far, it is not clear whether a coupling of these pathways is possible under long-term anoxic conditions, or, which substrates are involved directly or indirectly, such as ethanol, hydrogen etc. Further physiological experiments are needed to clarify, whether enzymatic reactions are similar to those of methanogens, SRB or acetogens (Buckel and Thauer, 2013) are possible in LSB.

In line with the dual nitrate reduction and possiblly dual carbon fixation pathways identified here, the identification of multiple alternate pathways for energy conservation in *Thiomargarita* may reflect their adaptation to a non-motile lifestyle in a highly fluctuating environment with reoccurring, but, dynamic changes of redox potentials and nutrient supplies. The adaptation with a broad genetic repertoire appears to be a survival strategy in *Thiomargarita*, and, to a certain extent, also in the other LSB.

### Intracellular Storage

It is well documented that at least some LSB can store several organic and inorganic compounds as granules in the periplasm, in the cytoplasm, or dissolved in the central vacuole (Schulz and Schulz, 2005; Brock and Schulz-Vogt, 2011; Teske and Salman, 2014). In all analyzed LSB we found a complete set of genes for the uptake of inorganic phosphate and the production of polyphosphate. The production of organic storage compounds in the form of polyhydroxybutyrate (PHB) was only found in *T. ingrica*, *B. alba* B18LD, and *Beggiatoa* sp. strain 35Flor (**Figure 2**), which all also encode the glyoxylate bypass to use acetate as an additional carbon source. Further details are described in the Supplementary Information.

## Conclusion

*Thiomargarita* species occur globally and are involved in biogeochemical cycles that are important for ecosystem functioning. Despite their conspicuous size and general sulfur-oxidizing characteristics little is known about their genetic potential. Here, we present the genomic features of a single-cell of “*Ca.* Thiomargarita nelsonii Thio36” and its potential for chemolithotrophy, carbon assimilation, energy metabolism and storage of nutrients.

The genomic comparison of “*Ca*. T. nelsonii Thio36” to the other large colorless SOB showed that they share a large number of metabolic capabilities such as the complete TCA cycle and glycolysis, carbon fixation via the CBB-cycle, energy conservation via the oxidative phosphorylation, sulfide oxidation, polyphosphate syntheses, potential hydrogen oxidation, and Na^+^-translocating membrane complexes (**Table 2**). Separating the genus *Thiomargarita* from other members of the family, however, was the trait that the two “*Ca*. T. nelsonii” genomes often encode dual mechanisms to convert substrates. This might be a special adaptation to a non-motile lifestyle, yet, thriving in an environment with rapidly changing redox conditions. They possess two nitrate reduction pathways, the CBB and potentially the rTCA carbon fixation pathways, and versatile oxidative metabolisms involving different reduced sulfur compounds, hydrogen and even organic molecules. Moreover, the “*Ca*. T. nelsonii Thio36” genome contains a new, only recently proposed energy conserving pathway known as FBEB for surviving anaerobic conditions under the absence of suitable electron acceptors for respiration of H_2_. This metabolic feature could be relevant in facultative anaerobic strains exposed to long-term sulfidic conditions in the environment, as it can provide enough energy for ATP generation. Interestingly, phylogenetically closely-related LSB, such as the two sequenced “*Ca*. T. nelsonii” cells (**Figure 1B**), show partially different sets of functional genes, e.g., types of RuBisCO (**Figure 3**), that point toward divergent evolutionary developments during the adaptation to different environmental niches.

A high degree of sequence similarity between genes from LSB to those in filamentous cyanobacteria has been demonstrated (Mußmann et al., 2007; MacGregor et al., 2013c; Flood et al., 2014), and includes the genomes of “*Ca*. T. nelsonii” as shown in our study (Supplementary Table S3). This further supports early and extensive horizontal gene transfer between ancestors of these groups. However, a modern coexistence of cyanobacteria and *Thiomargarita* spp. has so far never been reported, unlike the often encountered co-inhabitation of microbial mats by filamentous cyanobacteria and other LSB (Teske and Salman, 2014).

The here presented data on the metabolic potential of “*Ca.* T. nelsonii” based on the first insights into the genomes of the genus *Thiomargarita* opens a large window of opportunity for hypothesis-driven ecophysiological experiments. These days, a suite of culture-independent methods is available that can be used to study LSB. Metatranscriptomic and -proteomics approaches may shed light on LSB communities reacting to changes in their environment as exemplified in (Jones et al., 2015, 2016), and single-cell approaches such as mRNA FISH or nano-SIMS could be used with these easily identifiable cells to monitor the differential expression of alternative pathways and the differential incorporation of external resources.

## Author Contributions

MW, VS-C, and MM conceived of this study. HNS collected samples. MW and VS-C extracted the single cell from the sheath and removed contaminants. MW performed the DNA extraction and PCR screening of the single-cell *Thiomargarita* samples. TW performed the Illumina DNA sequencing and read assembly, TW, and MR performed the genome annotation. MW analyzed the sequence data with the assistance of MR and MM. MW performed phylogenic analyses, BEF and JVB performed the assembly of the Bud S10 genome. MW wrote the manuscript with input from all authors. All authors read and approved the final version of the manuscript.

## Conflict of Interest Statement

The authors declare that the research was conducted in the absence of any commercial or financial relationships that could be construed as a potential conflict of interest.
