# Supplementary material for: Single-cell Sequencing of Thiomargarita Reveals Genomic Flexibility for Adaptation to Dynamic Redox Conditions
Source: Front Microbiol. 2016 Jun 21;7:964. doi: 10.3389/fmicb.2016.00964 (PMC4914600; doi:10.3389/fmicb.2016.00964)
Supplement: Supplementary file 1 [file Data_Sheet_1.DOCX]

Supplementary Material

Single-cell sequencing of *Thiomargarita* reveals genomic flexibility for adaptation to dynamic redox conditions

**Matthias Winkel^*^, Verena Salman, Tanja Woyke, Michael Richter, Heide Schulz-Vogt, Beverly Flood, Jake Bailey, and Marc Mußmann^*^**

*** Correspondence:**

Dr. Matthias Winkel

Department of Geoarchives, Section 5.3 Geomicrobiology

Helmholtz Centre Potsdam, GFZ German Research Centre for Geosciences

Telegrafenberg, Building C, room 427,

14473 Potsdam, Germany

mwinkel@gfz-potsdam.de

Dr. Marc Mussmann

Department of Microbial Ecology, University of Vienna

Althanstrasse 14, A-1090 Vienna

mussmann@microbial-ecology.net

Supplementary Data

# Material methods

## Amplification of different genes for post-MDA testing

To test for purity we amplified the 16S rRNA gene of the Thio36-MDA product with different primer combinations (Table S1). Different dilutions of the MDA-product were used as template in a 20 µl PCR mix, containing 1 x PCR buffer (500 mM KCl, 100 mM Tris-HCl pH 8.3 at 25°C, 15 mM Mg^2+^) (5 Prime, Hamburg, Germany), 0.2 mM of each deoxynucleoside (Roche, Basel, Schweiz), 0.5 µM of each primer (Biomers, Ulm, Deutschland), 0.3 mg ml^-1^ BSA (Fluka, Buchs, Schweiz) and 0.01 U µl^-1^ *Taq*-polymerase (5 Prime, Hamburg, Germany). Primers were synthesized by Biomers (Ulm, Germany). The thermocycler conditions were as follows: 94°C for 5 minutes (denaturation), followed by 30 cycles of 94°C for 1 minute (denaturation), 1.5 minutes annealing (for temperatures see table S1) and 1 minute elongation at 72°C.

# Results and Discussion

## Phylogenetic gene amplification control of target DNA

Whole genome amplification by MDA is prone to amplification of non-target DNA from contaminating organisms. To detect potentially contaminating bacterial DNA during the MDA of "*Ca.* T. nelsonii" single cells all MDA product were tested by PCR for the full-length 16S rRNA gene. The 16S rRNA gene sequence in "*Ca.* T. nelsonii" is approximately 2,350 nt in length due to an intron of 855 nt (Salman *et al.*, 2012). This unique feature allowed us to discriminate against other bacterial 16S rRNA genes with length of approximately 1500 nt, which would have been preferentially amplified if present (Salman *et al.*, 2012). We detected no contamination during the genome amplification with this test.

## Nitrogen metabolism

The membrane-bound dissimilatory nitrate reductase (*nar*) of "*Ca*. T. nelsonii Thio36" is arranged in a *narGHJI* operon, similar to those of other *Gammaproteobacteria* (Table S2) (Moreno-Vivián *et al.*, 1999). A high degree of *nar* gene synteny is found in "*Ca.* Maribeggiatoa sp. ", *T. ingrica*, and "*Ca.* T. nelsonii Bud S10", while they are distributed over different contigs in "*Ca*. I. divolgata" (Table S2) (Mußmann *et al.*, 2007). The marine *Beggiatoa* sp. strain 35Flor and the freshwater strain *B. alba* B18LD lack genes for the membrane-bound nitrate reductase (Table S2). The two "*Ca.* T. nelsonii" genomes also encode a second set of the *narGH* nitrate reductase, similar as in "*Ca.* I. divolgata" and "*Ca.* Maribeggiatoa sp." (Table S2). "*Ca.* T nelsonii Bud S10" also encode a second *narI* downstream of the second *narGH* genes. Furthermore, *T. ingrica*, "*Ca.* T. nelsonii" and "*Ca.* I. divolgata" contained nitrate/nitrite antiporter (*narK*) potentially involved in the accumulation of nitrate in the central vacuole (Beutler *et al.*, 2012). The detected genes for the periplasmic dissimilatory nitrate reductase (*napABCGH*) (Morozkina and Zvyagilskaya, 2007) of the "*Ca.* T. nelsonii Thio36" genome are located on different contigs, while only *napA* and *napG* are on the same contig. Both genes are present twice in the genome, but at different locations. All other LSB with the exceptions of *Tp. ingrica* and *Beggiatoa* sp. strain 35Flor contain genes for the same periplasmatic nitrate reductase, although the ferredoxin-type protein *napG* only occured in *B. alba* B18LD and "*Ca.* T. nelsonii Bud S10", and the ferredoxin-type protein *napH* are missing with the exception of the "Ca. T. nelsonii Bud S10" genome (Table S2). The assimilatory nitrate reductase (*nasA*) can be identified in the genome of "*Ca.* T. nelsonii Thio36", "*Ca*. T. nelsonii Bud S10", *T. ingrica*, and *B. alba* B18LD (Fig. 2 and Table S2). In the almost closed *B. alba* B18LD genome, the gene is located next to the NAD(P)H-dependent nitrite reductase *nirBD*. This nitrite reductase is known to be involved in the assimilatory (Malm *et al.*, 2009) and dissimilatory nitrite reduction (Zumft, 1997). The "*Ca.* T. nelsonii Thio36" genome also encodes the nitrite reductase large subunit (*nirB*), but lacks the small subunit (*nirD*), which might be located on the not yet sequenced part of the genome. This assumption is further supported by a full set of *nirBD* on the "*Ca.* T. nelsonii Bus S10" genome. If "*Ca.* T. nelsonii Thio36" encodes the full set of the *nirBD* gene besides the *narGHIJ* and *napABCGH* operon, a dissimilatory nitrate reduction to ammonia would be possible. DNRA is likely to occur in *B. alba* B18LD via *nirBD* and *napABCGH,* as DNRA occurrence was experimentally shown in earlier studies with *B. alba* (Vargas and Strohl, 1985). The marine *Beggiatoa* genomes do not encode nitrite reductases involved in DNRA, but an isolated and purified multiheme cytochrome of "*Ca.* Maribeggiatoa sp." showed nitrite reductase-function (MacGregor *et al.*, 2013), and may be involved in DNRA. The same cytochrome was found in "*Ca.* I. divolgata" (MacGregor *et al.*, 2013), while it has not been detected in *Beggiatoa* sp. strain 35Flor (Fig. 2 and Table S2).

For a potential denitrification pathway, the genomes of "*Ca.* T. nelsonii Thio36", "*Ca*. T nelsonii Bud S10", *T. Ingrica*, and "*Ca.* I. divolgata" contain an almost complete *nirSCFDLGHJEN* operon of the nitrite reductase that only lacks some enzymes involved in heme biosynthesis ("*Ca.* T. nelsonii Thio36" 🡪 *nirHEL*, "*Ca*. T. nelsonii Bud S10" 🡪 *nirLGH* and "*Ca.* I. divolgata" and *T. ingrica* 🡪 *nirEN*; Table S2). This operon is known to occur in the denitrifying bacterium *Pseudomonas* *aeroginosa* (Bedzyk *et al.*, 1999) and together with the periplasmatic nitrate reductase *napABCGH* indicates the potential for denitrification in "*Ca.* T. nelsonii Thio36", "*Ca*. T. nelsonii Bud S10", *T. ingrica* and "*Ca.* I. divolgata". Furthermore, all four genomes and "*Ca.* Maribeggiatoa sp." encoded the membrane-bound nitric oxide reductase complex *norBC*, and *norQDEF* (Table S2). *NorE* is only present in "*Ca.* T. nelsonii Thio36", while *norF* is not encoded by any genome. In "*Ca.* T. nelsonii Thio36", one contig is organized in the order *norQEB*. *NorQ* is flanked by two hypothetical membrane proteins that are potentially anchor proteins. Another contig contains a second *norQB* gene. The *norQDEF*-complex analyzed in denitrifiying microorganisms was shown to have regulatory effects, while nitrate and nitrite reduction was reduced by knockout of *norQ*. However, *norE* has similarities to cytochrome oxidase subunit III and knockouts effectively reduce nitric oxide reductase activity (Baker *et al.*, 1998). Besides the Nor-complex "*Ca.* T. nelsonii Thio36", *B. alba* B18LD, and *Beggiatoa* sp. strain 35Flor also contain genes encoding a cyanide-sensitive nitric oxide dioxygenase (Table S2), which produces nitrate and has similar structures to flavohemoglobin from *Escherichia coli*. This enzyme appears to be ancient in the superfamily of hemoglobins (Gardner *et al.*, 1998) and a potential function in large colorless SOB is unclear.

## Sulfur oxidation pathways

Besides sulfide oxidation genes, we further found genes for the oxidation of the internally stored zerovalent sulfur encoded by the reverse dissimilatory sulfite reductase (rDSR) pathway. In the genome of "*Ca.* T. nelsonii Thio36" only some of these genes are present. We found a partial *dsrA*, *dsrB*, *dsrE*, *dsrC*, *dsrR*, *dsrS*, and contigs with *dsrMK* and *dsrKL* genes, so functionality in "*Ca.* T. nelsonii Thio36" is unclear (Fig.2 and Table S4). Nevertheless, a complete operon was found in the genomes of *T. ingrica, Beggiatoa* sp. strain 35Flor, and "*Ca.* Maribeggiatoa sp.", as well as larger contigs with parts of the operon in "*Ca.* I. divolgata" (Fig. 2 and Table S3).

The produced sulfite of the rDSR pathway is oxidized indirectly by the AMP-dependent oxidation, which is catalyzed by the cytoplasmic adenosine-5´-phosphosulfate (APS) reductase and the ATP sulfurylase via substrate level phosphorylation to produce sulfate (Hagen and Nelson, 1997; Dahl *et al.*, 2008). A gene encoding a partial alpha subunit of the APS reductase (*aprA*), and a gene encoding the ATP sulfurylase (*sat*) are present in the genome of „*Ca*. T. nelsonii Thio36“. A full set of *aprAB* and *sat* genes can be found in "*Ca.* I. divolgata", "*Ca*. T nelsonii Bud S10", *T. ingrica* and *Beggiatoa* sp. strain 35Flor, so a complete set of genes in "*Ca.* T. nelsonii Thio36" can be assumed. Furthermore, genes for the assimilatory sulfate reduction (*cysCDNHIJ*) (Neumann *et al.*, 2000) together with a sulfate permease ABC transporter (*cysAWT*) for sulfur assimilation are encoded on the *B. alba* B18LD genome (Fig. 2 and Table S4). It lacks genes for the dissimilatory sulfite oxidation (*aprAB* and *sat*) and only a few genes of the rDSR pathway (Table S4). The latter genes might be relicts of an obligate chemolithotrophic lifestyle. *Beggiatoa* sp. strain 35Flor also has genes for the assimilatory sulfate reduction via a high affinity sulfate permease transporter (SuIP), a sulfate adenylytransferase (*sat*), an adenylylsulfate (APS) kinase (*cysC*), a phosphor-adenylylsulfate (PAPS) reductase (*cysH*), and a ferredoxin sulfite reductase (*sir*) (Table S4) that produces hydrogen sulfide. This sulfide may be incorporated into cysteine for sulfur assimilation. We also found genes involved in thiosulfate oxidation by the SOX pathway (Dahl *et al.*, 2008). The genes *soxBY*, are encoded on the genomes of "*Ca.* T. nelsonii Thio36", "*Ca*. T. nelsnoii Bud S10", "*Ca.* I. divolgata", *T*. i*ngrica*, *Beggiatoa* sp. strain 35Flor, and "*Ca.* Maribeggiatoa sp.". Another gene involved in thiosulfate utilization present on the genome of "*Ca*. T. nelsonii Thio36" is a thiosulfate sulfurtransferase (rhodanese) known from the thiosulfate disproportionation pathway (Table S4). Rhodaneses are assumed to be responsible for cyanide detoxification (Cipollone *et al.*, 2006), but can be involved in sulfur oxidation of chemolithautotrophic microorganisms as they convert thiosulfate to sulfite (Anantharaman *et al.*, 2013; Sheik *et al.*, 2013). A detailed description of the sulfur oxidation in *Beggiatoaceae* is published elsewhere (Kreutzmann, 2013).

## Carbon metabolism

### Glycolysis

Besides the mentioned polyphosphate glucokinase (*ppgk*), "*Ca.* I. divolgata" and *T. ingrica* also encode an ATP-glucokinase. This enzyme was detected in *B. alba* B18LD and *Beggiatoa* sp. 35Flor that lack the polyphosphate-dependent form. The polyphosphate-dependent type of this enzyme has been speculated to be an ancient form out of which the modern ATP-dependent type evolved (Tanaka *et al.*, 2003). The occurrence of polyphosphate-dependent enzymes supports the observation that *Thiomargarita* species and *Beggiatoa* sp. strain 35Flor store polyphosphate internally as granula (Schulz and Schulz, 2005; Brock *et al.*, 2012). In contrast to all other investigated LSB "*Ca.* T. nelsonii Thio36" and "*Ca*. T. nelsnoii Bud S10" encode for a class I aldolase (Table S4), which is a typically found in animals and plants and has only been rarely detected in microorganisms.

All analyzed LSB genomes encode enzymes converting the produced pyruvate to acetyl-CoA and shuttling it into the tricarboxylic acid cycle, with the exception of the "*Ca.* Maribeggiatoa sp.", which lacked one component (*aceF/pdhC*) of the multienzyme pyruvate dehydrogenase complex (Table S4). Further support for carbohydrate utilization was the detection of multiple gene copies for saccharide ABC transporter systems (data not shown).

### Pyrophosphatase-coupled carbon fixation

In endosymbiontic sulfur-oxidizing and methanotrophic bacteria a coupling between the pyrophosphate-forming 6-phosphofructokinase, and a membrane-bound proton-tanslocating pyrophosphatase for energy conservation has been proposed (Reshetnikov *et al.*, 2008; Kleiner *et al.*, 2012b). Unlike these microorganisms, LSB do not encode a proton-translocating pyrophosphatase in close proximity to the genome region of the pyrophosphate-dependent 6-phosphofructokinase (Kleiner *et al.*, 2012b). Nevertheless, we found at least one proton-translocating pyrophosphatase on the genomes of all LSB with the exception of "*Ca.* T. nelsonii Thio36", which might function as an energy-saving alternative track of the CBB-cycle (Kleiner *et al.*, 2012a).

### C2-cycle and an alternative phosphoserine shuttle

Accumulation of glycolate under high O_2_ concentrations could be a side reaction resulting from chemorespiration in chemoautotrophic bacteria similar to photorespiration in plants, algae and cyanobacteria (Bowien and Schlegel, 1981). Hence, an active oxygenase activity of the RubisCO from chemoautotrophic bacteria can be expected.

The genome of "*Ca.* T. nelsonii Thio36" encodes an almost complete set of genes involved in the C2-cycle (Fig. 4), with key enzymes such as 2-phosphoglycolate phosphatase (*gph*), glycolate oxidase (*glcDEF*), and hydroxypyruvate reductase (*ttuD*) (Table S4). It is most likely that the RubisCO of "*Ca*. T. nelsonii Thio36" produces 2-PG under oxic conditions. However, a glycerate kinase of the class III (GLYK) (Table S4), which is essential to produce D-3-phosphogylcerate (3-PG) to be shuffled back into the CBB-cycle, has not been found in the genome. The produced organic carbon is not lost for the cells and an inhibition of the CBB-cycle by accumulation of 2-PG is prevented. Homolog genes were found in all other LSB, with the exception of "*Ca.* Maribeggiatoa sp.", which lacks a glycolate oxidase (Table S4). Besides this, *B. alba* B18LD, *Beggiatoa* sp. strain Flor36, and "*Ca.* Maribeggiatoa sp." do not encode a serine-pyruvate aminotransferase, which converts serine and glyoxylate to hydroxypyruvate and glycine. Nevertheless, they encode aminotransferases class V that belong to the same subfamily as serine-pyruvate aminotransferases. This subfamily has the same mechanistic features and high sequence identities.

Strikingly, all genomes also contain multiple copies of serine/ threonine kinase (STPK), which are involved in the phosphorylation of serine and play an important role in the signal transduction (Pereira *et al.*, 2011). If the produced serine in the C2-cycle can be phosphorylated by STPK, phosphoserine can be further converted by the gene phosphoserine aminotransferases (*serC*) producing 3-phospho-hydroxypyruvate. Another possible enzyme for the phosphorylation of serine is a phosphoserine phosphatase (*serB*). The produced 3-phospho-hydroxypyruvate by *serC* can be converted directly to 3-phospho-D-glycerate by the D-3-phosphoglycerate dehydrogenase (*serA*). Indeed, the genomes of "*Ca.* T. nelsonii Thio36", "*Ca*. T. nelsonii Bud S10", *T. ingrica*, "*Ca.* I. divolgata", and *B. alba* B18LD contain homologous genes for these enzymes, while "*Ca*. T. nelsonii Bud S10", *T. ingrica*, and "*Ca.* I. divolgata" lack *serB*, and *B. alba* B18LD lacks *serA*. So it is possible that they use an alternative pathway to shuffle the produced serine back into the C2-cycle, and therefore do not need a glycerate kinase (Fig. 4 and Table S4). While they still have the hydroxypyruvate reductase it is not clear whether the incompleteness of the genomes explains the lack of the essential glycerate kinase type III. Only recently, it was proven that a mutant of the cyanobacterium *Synchocystis* sp. strain PCC 6803, which has an inactivation in the same serine/threonine kinase (STPK) that occurs in LSB, is impaired in growing under low inorganic carbon conditions (Laurent *et al.*, 2008). This behavior could be induced by a higher oxygenase activity of RubisCO and lead to a higher production of 2-PG inhibiting the CBB-cycle. While *Synchocystis* sp. strain PCC 6803 is known to express all genes involved in the C2-cycle (Eisenhut *et al.*, 2008) the genome also has a full set of the proposed alternative pathway via STPK, *serC,* and *serA* (Fig. 4) (Kaneko *et al.*, 1996). Perhaps cyanobacteria and large, colorless SOB use the alternative pathways to convert 2-PG under specific environmental conditions. Physiological experiments are necessary to elucidate the occurrence and usage of the alternative pathway (phosphoserine shuttle).

## Intracellular storage compounds

LSB are known to feature different storage capacities for organic and inorganic compounds such as sulfur, phosphorous, nitrate, and carbon. If cells are large enough and have a central vacuole, they accumulate nitrate (Fossing *et al.*, 1995; McHatton *et al.*, 1996; Schulz *et al.*, 1999). In the genomes of "*Ca.* T. nelsonii Thio36", "*Ca*. T nelsonii Bud S10" and "*Ca.* I. divolgata" we find nitrate/nitrite transporters, cytochrome c oxidases, v-type ATPases, and cytochrome c that could be involved in energy conservation via a proton motive force over the vacuole membrane like in "*Ca.* Allobeggiatoa" (Beutler *et al.*, 2012).

In all analyzed genomes we find subunits of phosphate-specific transporters (*pst*) located on the *pstSCABphoU* operon, which together with the polyphosphate synthesizing enzyme polyphosphate kinase (*ppk*) are part of the well described Pho-regulon (Vershinina and Znamenskaya, 2002) (Fig. 2 and Table S4). The inorganic phosphate uptake by the ATP-dependent Pst in the cytoplasmic membrane is transcriptionally regulated by the two-component system *phoR-phoB*. The *phoB* that transcriptionally regulates the phosphate regulon is only present in „*Ca.* Maribeggiatoa sp.“, while the phosphor sensor regulon protein *phoR* is present in all other investigated genomes (Table S4). The genomes of "*Ca.* T. nelsonii Thio36", "*Ca.* I. divolgata", and "*Ca.* Maribeggiatoa sp." also contain homologous genes for the outer membrane porines O and P (*phoE*) that are specific for the uptake of orthophosphate and polyphosphate, respectively (Vershinina and Znamenskaya, 2002) (Table S4). All of these genes are highly up-regulated in microorganisms under phosphorous-limiting conditions, and are responsible for inorganic phosphate accumulation (Vershinina and Znamenskaya, 2002). The accumulation and storage of polyphosphate in *T. namibiensis* and *Beggiatoa* sp. strain 35Flor was visualized by internal granules (Schulz and Schulz, 2005; Brock and Schulz-Vogt, 2011).

Besides the storage and accumulation of inorganic compounds, *Beggiatoa* store polyhydroxyalkanotes (PHA) in granules in their cytoplasm, most often in the form of polyhydroxybutyrate (PHB) (Pringsheim, 1964; Strohl and Larkin, 1978). Genes involved in the synthesis of polyhydroxybutyrate (PHB) are only found in the genomes of *T. ingrica*, *B. alba* B18LD and *Beggiatoa* sp. strain 35Flor that also have the glyoxylate bypass to use acetate as a carbon source (Fig. 2 and Table S4).

# References

Anantharaman, K., Breier, J. A., Sheik, C. S., and Dick, G. J. (2013). Evidence for hydrogen oxidation and metabolic plasticity in widespread deep-sea sulfur-oxidizing bacteria. *Proc. Natl. Acad. Sci. U.S.A.* 110, 330–335. doi:10.1073/pnas.1215340110.

Baker, S. C., Ferguson, S. J., Ludwig, B., Page, M. D., Richter, O.-M. H., and Spanning, R. J. M. van (1998). Molecular genetics of the genus *Paracoccus*: Metabolically versatile bacteria with bioenergetic flexibility. *Microbiol. Mol. Biol. Rev.* 62, 1046–1078.

Bedzyk, L., Wang, T., and Ye, R. W. (1999). The periplasmic nitrate reductase in *Pseudomonas* sp. strain G-179 catalyzes the first step of denitrification. *J. Bacteriol.* 181, 2802–2806.

Beutler, M., Milucka, J., Hinck, S., Schreiber, F., Brock, J., Mußmann, M., *et al.* (2012). Vacuolar respiration of nitrate coupled to energy conservation in filamentous *Beggiatoaceae*. *Environ. Microbiol.* 14, 2911–2919. doi:10.1111/j.1462-2920.2012.02851.x.

Bowien, B., and Schlegel, H. G. (1981). Physiology and biochemistry of aerobic hydrogen-oxidizing bacteria. *Annu. Rev. Microbiol.* 35, 405–452. doi:10.1146/annurev.mi.35.100181.002201.

Brock, J., Rhiel, E., Beutler, M., Salman, V., and Schulz-Vogt, H. N. (2012). Unusual polyphosphate inclusions observed in a marine *Beggiatoa* strain. *Antonie Van Leeuwenhoek* 101, 347–357. doi:10.1007/s10482-011-9640-8.

Brock, J., and Schulz-Vogt, H. N. (2011). Sulfide induces phosphate release from polyphosphate in cultures of a marine *Beggiatoa* strain. *ISME J.* 5, 497–506. doi:10.1038/ismej.2010.135.

Brosius, J., Palmer, M. L., Kennedy, P. J., and Noller, H. F. (1978). Complete nucleotide sequence of a 16S ribosomal RNA gene from *Escherichia coli*. *Proc. Natl. Acad. Sci. U.S.A.* 75, 4801–4805.

Cipollone, R., Ascenzi, P., Frangipani, E., and Visca, P. (2006). Cyanide detoxification by recombinant bacterial rhodanese. *Chemosphere* 63, 942–949. doi:10.1016/j.chemosphere.2005.09.048.

Dahl, C., Friedrich, C., and Kletzin, A. (2008). “Sulfur Oxidation in Prokaryotes,” in *eLS*

Eisenhut, M., Ruth, W., Haimovich, M., Bauwe, H., Kaplan, A., and Hagemann, M. (2008). The photorespiratory glycolate metabolism is essential for cyanobacteria and might have been conveyed endosymbiontically to plants. *Proc. Natl. Acad. Sci. U.S.A.* 105, 17199–17204. doi:10.1073/pnas.0807043105.

Fossing, H., Gallardo, V. A., Jørgensen, B. B., Huttel, M., Nielsen, L. P., Schulz, H., *et al.* (1995). Concentration and transport of nitrate by the mat-forming sulphur bacterium *Thioploca*. *Nature* 374, 713–715. doi:10.1038/374713a0.

Gardner, P. R., Gardner, A. M., Martin, L. A., and Salzman, A. L. (1998). Nitric oxide dioxygenase: An enzymic function for flavohemoglobin. *Proc. Natl. Acad. Sci. U.S.A..* 95, 10378–10383.

Hagen, K. D., and Nelson, D. C. (1997). Use of reduced sulfur compounds by *Beggiatoa* spp.: Enzymology and physiology of marine and freshwater strains in homogeneous and gradient cultures. *Appl. Environ. Microbiol.* 63, 3957–3964.

Kaneko, T., Sato, S., Kotani, H., Tanaka, A., Asamizu, E., Nakamura, Y., *et al.* (1996). Sequence analysis of the genome of the unicellular cyanobacterium *Synechocystis* sp. strain PCC6803. II. Sequence determination of the entire genome and assignment of potential protein-coding regions. *DNA Res.* 3, 109–136. doi:10.1093/dnares/3.3.109.

Kleiner, M., Petersen, J. M., and Dubilier, N. (2012a). Convergent and divergent evolution of metabolism in sulfur-oxidizing symbionts and the role of horizontal gene transfer. *Curr. Opin. Microbiol.* 15, 621–631. doi:10.1016/j.mib.2012.09.003.

Kleiner, M., Wentrup, C., Lott, C., Teeling, H., Wetzel, S., Young, J., *et al.* (2012b). Metaproteomics of a gutless marine worm and its symbiotic microbial community reveal unusual pathways for carbon and energy use. *Proc. Natl. Acad. Sci. U.S.A.* 109, 7148–7149. doi:10.1073/pnas.1121198109.

Kreutzmann, A.-C. (2013). Electron donors and acceptors for members of the family Beggiatoaceae. [dissertation]. [Bremen]; Universität Bremen.

Laurent, S., Jang, J., Janicki, A., Zhang, C.-C., and Bédu, S. (2008). Inactivation of spkD, encoding a Ser/Thr kinase, affects the pool of the TCA cycle metabolites in Synechocystis sp. strain PCC 6803. *Microbiology* 154, 2161–2167. doi:10.1099/mic.0.2007/016196-0.

MacGregor, B. J., Biddle, J. F., Siebert, J. R., Staunton, E., Hegg, E. L., Matthysse, A. G., *et al.* (2013). Why orange Guaymas Basin *Beggiatoa* spp. are orange: Single-filament-genome-enabled identification of an abundant octaheme cytochrome with hydroxylamine oxidase, hydrazine oxidase, and nitrite reductase activities. *Appl. Environ. Microbiol.* 79, 1183–1190. doi:10.1128/AEM.02538-12.

Malm, S., Tiffert, Y., Micklinghoff, J., Schultze, S., Joost, I., Weber, I., *et al.* (2009). The roles of the nitrate reductase NarGHJI, the nitrite reductase NirBD and the response regulator GlnR in nitrate assimilation of *Mycobacterium tuberculosis*. *Microbiology* 155, 1332–1339. doi:10.1099/mic.0.023275-0.

Manz, W., Amann, R., Ludwig, W., Wagner, M., and Schleifer, K.-H. (1992). Phylogenetic oligodeoxynucleotide probes for the major subclasses of *Proteobacteria*: Problems and solutions. *Syst. Appl. Microbiol.* 15, 593–600. doi:10.1016/S0723-2020(11)80121-9.

McHatton, S. C., Barry, J. P., Jannasch, H. W., and Nelson, D. C. (1996). High nitrate concentrations in vacuolate, autotrophic marine *Beggiatoa* spp. *Appl. Environ. Microbiol.* 62, 954–958.

Moreno-Vivián, C., Cabello, P., Martínez-Luque, M., Blasco, R., and Castillo, F. (1999). Prokaryotic nitrate reduction: Molecular properties and functional distinction among bacterial nitrate reductases. *J. Bacteriol.* 181, 6573–6584.

Morozkina, E. V., and Zvyagilskaya, R. A. (2007). Nitrate reductases: Structure, functions, and effect of stress factors. *Biochem. (Mosc.)* 72, 1151–1160. doi:10.1134/S0006297907100124.

Mußmann, M., Hu, F. Z., Richter, M., de Beer, D., Preisler, A., Jørgensen, B. B., *et al.* (2007). Insights into the genome of large sulfur bacteria revealed by analysis of single filaments. *Plos Biol.* 5, e230. doi:10.1371/journal.pbio.0050230.

Muyzer, G., Teske, A., Wirsen, C., and Jannasch, H. (1995). Phylogenetic relationships of *Thiomicrospira* species and their identification in deep-sea hydrothermal vent samples by denaturing gradient gel electrophoresis of 16S rDNA fragments. *Arch. Microbiol.* 164, 165–172. doi:10.1007/BF02529967.

Neumann, S., Wynen, A., Trüper, H. G., and Dahl, C. (2000). Characterization of the *cys* gene locus from *Allochromatium vinosum* indicates an unusual sulfate assimilation pathway. *Mol. Biol. Rep.* 27, 27–33. doi:10.1023/A:1007058421714.

Pereira, S. F. F., Goss, L., and Dworkin, J. (2011). Eukaryote-like serine/threonine kinases and phosphatases in bacteria. *Microbiol. Mol. Biol. Rev.* 75, 192–212. doi:10.1128/MMBR.00042-10.

Pringsheim, E. G. (1964). Heterotrophism and species concepts in *Beggiatoa*. *Am. J. Bot.* 51, 898–913. doi:10.2307/2439898.

Reshetnikov, A. S., Rozova, O. N., Khmelenina, V. N., Mustakhimov, I. I., Beschastny, A. P., Murrell, J. C., *et al.* (2008). Characterization of the pyrophosphate-dependent 6-phosphofructokinase from *Methylococcus capsulatus* Bath. *FEMS Microbiol. Lett.* 288, 202–210. doi:10.1111/j.1574-6968.2008.01366.x.

Salman, V., Amann, R., Shub, D. A., and Schulz-Vogt, H. N. (2012). Multiple self-splicing introns in the 16S rRNA genes of giant sulfur bacteria. *Proc. Natl. Acad. Sci. U.S.A.* 109, 4203–4208. doi:10.1073/pnas.1120192109.

Schulz, H. N., Brinkhoff, T., Ferdelman, T. G., Mariné, M. H., Teske, A., and Jørgensen, B. B. (1999). Dense populations of a giant sulfur bacterium in Namibian shelf sediments. *Science* 284, 493–495. doi:10.1126/science.284.5413.493.

Schulz, H. N., and Schulz, H. D. (2005). Large sulfur bacteria and the formation of phosphorite. *Science* 307, 416–418. doi:10.1126/science.1103096.

Sheik, C. S., Jain, S., and Dick, G. J. (2013). Metabolic flexibility of enigmatic SAR324 revealed through metagenomics and metatranscriptomics. *Environ. Microbiol.*, n/a–n/a. doi:10.1111/1462-2920.12165.

Strohl, W. R., and Larkin, J. M. (1978). Enumeration, isolation, and characterization of *Beggiatoa* from freshwater sediments. *Appl. Environ. Microbiol.* 36, 755–770.

Tanaka, S., Lee, S.-O., Hamaoka, K., Kato, J., Takiguchi, N., Nakamura, K., *et al.* (2003). Strictly polyphosphate-dependent glucokinase in a polyphosphate-accumulating bacterium, *Microlunatus phosphovorus*. *J. Bacteriol.* 185, 5654–5656. doi:10.1128/JB.185.18.5654-5656.2003.

Vargas, A., and Strohl, W. R. (1985). Utilization of nitrate by *Beggiatoa alba*. *Arch. Microbiol.* 142, 279–284. doi:10.1007/BF00693404.

Vershinina, O. A., and Znamenskaya, L. V. (2002). The Pho regulons of bacteria. *Microbiology* 71, 497–511. doi:10.1023/A:1020547616096.

Wilmotte, A., Van der Auwera, G., and De Wachter, R. (1993). Structure of the 16 S ribosomal RNA of the thermophilic cyanobacterium chlorogloeopsis HTF (“*Mastigocladus laminosus* HTF”) strain PCC7518, and phylogenetic analysis. *FEBS Lett.* 317, 96–100. doi:10.1016/0014-5793(93)81499-P.

Zumft, W. G. (1997). Cell biology and molecular basis of denitrification. *Microbiol. Mol. Biol. Rev.* 61, 533–616.

# Supporting Tables:

**Table S1**: Oligonucleotides used in this study

| **name** | **sequence 3-5** | **target**  **gene** | **annealingT [°C]** | **Position^a^** | **combination** | **expected product size [bp]** | **reference** |
| --- | --- | --- | --- | --- | --- | --- | --- |
| GM3F | AGA GTT TGA TCM TGG C | 16S rRNA | 48 | 8 - 23 | GM4R | 2350 | (Muyzer *et al.*, 1995) |
| GM4R | TAC CTT GTT ACG ACT T | 16S rRNA | 48 | 1492 - 1507 | GM3F | 2350 | (Muyzer *et al.*, 1995) |
|  |  |  |  |  | 1099R | 1280 |  |
| GM5F | CCT ACG GGA GGC AGC AG | 16S rRNA | 55 | 341 - 357 | 907RM | 550 | (Muyzer *et al.*, 1995) |
| 1099F | GYA ACG AGC GCA ACC C | 16S rRNA | 50 | 1099 - 1114 | GM4R | 400 | (Wilmotte *et al.*, 1993) |
| 907RM | CCG TCA ATT CMT TTG AGT TT | 16S rRNA | 55 | 907 - 927 | GM5F | 550 | (Muyzer *et al.*, 1995) |
| ITS350F^b^ | AAT TAG GAA GCT GAT GTA AA | ITS |  | - | Gam42aR | 1200 | This study |
| Gam42aR | GCC TTC CCA CAT CGT TTC C | 23S rRNA |  | 1027 - 1043 | ITS350F | 1200 | (Manz *et al.*, 1992) |

^a^ corresponding nucleotide positions of the 16S rRNA of *Escherichia coli* according to (Brosius *et al.*, 1978)

^b^ ITS – intergenic spacer region between 16S rRNA and 23S rRNA

**Table S2:** Single copy genes found in "*Ca.* T. nelsonii Thio36"

|  | **COG domain** | **predicted protein** | **aa length** | | **e-value** | **percent identity** | | **closest homolog (BlastP)** | **product name** | **pfam domain** |
| --- | --- | --- | --- | --- | --- | --- | --- | --- | --- | --- |
| 1 | COG0012 | Predicted GTPase, probable translation factor | 361 | 0 | | 94.77 | "*Ca.*T. nelsonii Bud S10" Ga0063879 | | hypothetical protein | pfam01926 - 50S ribosome-binding GTPase pfam06071 - Protein of unknown function (DUF933) |
| 2 | COG0016 | Phenylalanine-tRNA synthethase alpha subunit | - | - | | - | - | | - | - |
| 3 | COG0048 | Ribosomal protein S12 | 124 | -81 | | 97.58 | "*Ca.*T. nelsonii Bud S10" Ga0063879 | | SSU ribosomal protein S12P | pfam00164 - Ribosom_S12_S23 |
| 4 | COG0049 | Ribosomal protein S7 | - | - | | - | - | | - | - |
| 5 | COG0052 | Ribosomal protein S2 |  |  | |  |  | |  |  |
| 6 | COG0080 | Ribosomal protein L11 | 143 | -93 | | 94.41 | "*Ca.*T. nelsonii Bud S10" Ga0063879 | | LSU ribosomal protein L11P | pfam00298 - Ribosomal_L11 pfam03946 - Ribosomal_L11_N |
| 7 | COG0081 | Ribosomal protein L1 | - | - | | - | - | | - | - |
| 8 | COG0087 | Ribosomal protein L3 | 212 | -128 | | 98.9 | "*Ca.*T. nelsonii Bud S10" Ga0063879 | | large subunit ribosomal protein L3 | pfam00297 - Ribosomal_L3 |
| 9 | COG0091 | Ribosomal protein L22 | - | - | | - | - | | - | - |
| 10 | COG0092 | Ribosomal protein S3 | - | - | | - | - | | - | - |
| 11 | COG0093 | Ribosomal protein L14 | 122 | -68 | | 96.36 | "*Ca.*T. nelsonii Bud S10" Ga0063879 | | LSU ribosomal protein L14P | pfam00238 - Ribosomal_L14 |
| 12 | COG0094 | Ribosomal protein L5 | 166 | -91 | | 96.4 | "*Ca.*T. nelsonii Bud S10" Ga0063879 | | LSU ribosomal protein L5P | pfam00281 - Ribosomal_L5 pfam00673 - Ribosomal_L5_C |
| 13 | COG0096 | Ribosomal protein S8 | 130 | -83 | | 96.92 | „*Ca.*T. nelsonii Bud S10“ Ga0063879 | | SSU ribosomal protein S8P | pfam00410 - Ribosomal_S8 |
| 14 | COG0097 | Ribosomal protein L6P/L9E | 181 | -116 | | 93.37 | "*Ca.*T. nelsonii Bud S10" Ga0063879 | | large subunit ribosomal protein L6 | pfam00347 - Ribosomal_L6 |
| 15 | COG0098 | Ribosomal protein S5 | 173 | -177 | | 98.27 | "*Ca.*T. nelsonii Bud S10" Ga0063879 | | small subunit ribosomal protein S5 | pfam00333 - Ribosomal_S5 pfam03719 - Ribosomal_S5_C |
| 16 | COG0099 | Ribosomal protein S13 | - | - | | - | - | | - | - |
| 17 | COG0100 | Ribosomal protein S11 | - | - | | - | - | | - | - |
| 18 | COG0102 | Ribosomal protein L13 | 144 | -94 | | 93.75 | "*Ca.*T. nelsonii Bud S10" Ga0063879 | | LSU ribosomal protein L13P | pfam00572 - Ribosomal_L13 |
| 19 | COG0103 | Ribosomal protein S9 |  |  | |  |  | |  |  |
| 20 | COG0172 | Seryl-tRNA synthetase | 424 | 0 | | 96.39 | "*Ca.*T. nelsonii Bud S10" Ga0063879 | | seryl-tRNA synthetase | pfam00587 - tRNA-synt_2b pfam02403 - Seryl_tRNA_N |
| 21 | COG0184 | Ribosomal protein S15P/S13E | 89 | -50 | | 92.13 | "*Ca.*T. nelsonii Bud S10" Ga0063879 | | SSU ribosomal protein S15P | pfam00312 - Ribosomal_S15 |
| 22 | COG0186 | Ribosomal protein S17 | 89 | -54 | | 93.26 | "*Ca.*T. nelsonii Bud S10" Ga0063879 | | SSU ribosomal protein S17P | pfam00366 - Ribosomal_S17 |
| 23 | COG0197 | Ribosomal protein L16/L10E | - | - | | - | - | | - | - |
| 24 | COG0200 | Ribosomal protein L15 | - | - | | - | - | | - | - |
| 25 | COG0201 | Preprotein translocase subunit SecY | - | - | | - | - | | - | - |
| 26 | COG0202 | DNA-directed RNA polymerase, alpha subunit | 331 | 0 | | 90.85 | "*Ca.*T. nelsonii Bud S10" Ga0063879 | | DNA-directed RNA polymerase, alpha subunit/40 kD subunit | pfam01000 - RNA_pol_A_bac pfam01193 - RNA_pol_L pfam03118 - RNA_pol_A_CTD |
| 27 | COG0256 | Ribosomal protein L18 | 117 | -64 | | 94.87 | "*Ca.*T. nelsonii Bud S10" Ga0063879 | | Ribosomal protein L18 | pfam00861 - Ribosomal_L18p |
| 28 | COG0495 | Leucyl-tRNA synthetase | - | - | | - | - | | - | - |
| 29 | COG0522 | Ribosomal protein S4 and related proteins | - | - | | - | - | | - | - |
| 30 | COG0525 | Valyl-tRNA synthetase | - | - | | - | - | | - | - |
| 31 | COG0533 | Metal-dependent proteases with  chaperone activity | - | - | | - | - | | - | - |
| 32 | COG1314 | Preprotein translocase SecG subunit | 125 | -53 | | 86.92 | "*Ca*. T. nelsonii" (unscreened) Ga0097846_11318 | | protein translocase subunit secG | pfam03840 - SecG |
| 33 | - | heat-inducible transcription repressor HrcA | 260 | -151 | | 92.7 | "*Ca.*T. nelsonii Bud S10" Ga0063879 | | heat-inducible transcription repressor HrcA | pfam01628 - HrcA |
| 34 | COG0806 | 16S rRNA processing protein RimM | 166 | -108 | | 94.58 | "*Ca.*T. nelsonii Bud S10" Ga0063879 | | 16S rRNA processing protein RimM | pfam01782 - RimM  pfam05239 - PRC |
| 35 | COG1185 | Polyribonucleotide nucleotidyltransferase (polynucleotide phosphorylase), PNPase | 187 | -106 | | 89.94 | "*Ca.*T. nelsonii Bud S10" Ga0063879 | | polyribonucleotide nucleotidyl-transferase | pfam01138 - RNase_PH  pfam03726 - PNPase |

**Table S3:** Annotated ORFs with similarity >70% to Cyanobacteria

| **product name^!^** | **sequence identity AA** | **COG-ID** | **pfam domain** | **flanking genes^¥^** | **GC content [%]** |
| --- | --- | --- | --- | --- | --- |
| 3-hydroxyacyl-CoA dehydrogenase | 87,6 | COG1250 | pfam00725 | B/C | 42.4 |
| Predicted RNA binding protein YcfA, dsRBD-like fold, HicA-like mRNA interferase family | 85,5 | COG1724 | pfam07927 | C | 39.7 |
| Uncharacterized conserved protein | 85,2 | COG1690 | pfam01139 | B/C | 48.9 |
| hypothetical protein | 83,7 | - | - | B | 32.5 |
| 4-aminobutyrate aminotransferase and related aminotransferases | 83,6 | COG0160 | pfam00202 | B/C | 48 |
| Dihydrofolate reductase | 83,1 | COG0262 | pfam01872 | B/C | 47.9 |
| CDP-paratose 2-epimerase | 81,6 | COG0451 | pfam01370 | B/C | 46.6 |
| Transglutaminase-like superfamily protein | 81,3 | - | pfam13471 | C | 50.4 |
| hypothetical protein | 80,3 | - | - | O | 41.9 |
| Uncharacterized protein conserved in bacteria | 80 | COG2929 | pfam04365 | -* | 36.1 |
| Predicted ATPase | 79,6 | COG4637 | pfam13304 | B | 44 |
| Predicted carbamoyl transferase, NodU family | 79,2 | COG2192 | pfam02543 | B/O | 41.6 |
| YcfA-like protein. | 78,9 | - | pfam07927 | B | 47.4 |
| hypothetical protein | 78,6 | - | - | B | 32.3 |
| hypothetical protein | 78,3 | - | - | B | 43.3 |
| luciferase-type oxidoreductase, BA3436 family | 78,2 | - | pfam00296 | B | 55.2 |
| glycine hydroxymethyltransferase | 77,9 | COG0112 | pfam00464 | B/O | 46.3 |
| Adenosylmethionine-8-amino-7-oxononanoate aminotransferase | 77,8 | - | pfam00202 | C/O | 47.4 |
| Uncharacterized conserved protein | 77,3 | COG1598 | - | B | 39.4 |
| asparagine synthase (glutamine-hydrolyzing) | 77,2 | - | pfam00733 pfam13537 | O | 44.4 |
| Mg-chelatase subunit ChlI | 77,1 | COG1239 | pfam07728 | B | 44.4 |
| hypothetical protein | 77,1 | - | - | B | 44.7 |
| hypothetical protein | 76,6 | - | - | B | 39.9 |
| Acyl-CoA synthetases (AMP-forming)/AMP-acid ligases II | 76,6 |  |  | -* | 50.9 |
| Predicted nuclease of restriction endonuclease-like (RecB) superfamily, DUF1016 family | 76,2 | COG4804 | pfam06250 | B | 40.8 |
| RecA-superfamily ATPases implicated in signal transduction | 76 | COG0467 | - | B | 44.1 |
| hypothetical protein | 75,9 | - | - | -^#^ | 45.3 |
| asparagine synthase (glutamine-hydrolysing) | 75,8 | - | pfam00733 pfam13537 | B | 42.8 |
| hypothetical protein | 75,7 | - | - | C | 47.5 |
| Predicted ATPase | 75,5 | COG4637 | pfam13304 pfam13812 | B | 30.1 |
| Growth inhibitor | 75 | COG2337 | pfam02452 | B | 45.5 |
| hypothetical protein | 75 | - | - | C | 47.3 |
| ABC-type bacteriocin/lantibiotic exporters, contain an N-terminal double-glycine peptidase domain | 74,8 | - | pfam00005 | B | 45.6 |
| Recombinational DNA repair ATPase (RecF pathway) | 74,5 | COG1195 | pfam13476 | - | 36.8 |
| XisH protein | 74,4 | - | pfam08841 | B | 35.5 |
| hypothetical protein | 74,3 | - | - | O | 41.7 |
| NAD(P)-dependent dehydrogenase, short-chain alcohol dehydrogenase family | 74 | COG1028 | pfam00106 | B | 54.2 |
| Adenylate and Guanylate cyclase catalytic domain-containing protein | 73,7 | - | pfam00211 | B | 39.3 |
| HicA toxin of toxin-antitoxin, | 73,7 | - | pfam07927 | B | 34.5 |
| Reverse transcriptase (RNA-dependent DNA polymerase) | 73,4 | - | pfam00078 | B | 41.1 |
| clan AA aspartic protease, AF_0612 family | 73,4 | - | pfam13975 | B | 38.1 |
| Uncharacterized protein conserved in bacteria | 73,3 | COG3041 | - | B | 38.3 |
| hypothetical protein | 73,2 | - | - | B | 46.6 |
| hypothetical protein | 73 | - | pfam05114 | B | 41.5 |
| sarcosine/dimethylglycine  N-methyltransferase | 72,9 | - | pfam08241 | B | 44.5 |
| hypothetical protein | 72,9 | - | - | C | 55.4 |
| Polyketide synthase modules and related proteins | 72,3 | COG3321 | pfam00698 | B | 43.2 |
| tRNA threonylcarbamoyl adenosine modification protein, Sua5/YciO/YrdC/YwlC family | 72,3 | COG0009 | pfam01300 | B/C | 43.6 |
| Putative restriction endonuclease | 72,2 | - | pfam05685 | B | 44.6 |
| ABC-type bacteriocin/lantibiotic exporters, contain an N-terminal double-glycine peptidase domain | 72,2 | COG2274 | pfam00005 | B | 44.7 |
| Acyl-CoA dehydrogenases | 72,2 | COG1960 | pfam00441 pfam02770 | C | 43.4 |
| ATPase involved in DNA repair | 72 | COG0419 | pfam13476 | B | 36.7 |
| XisI protein | 71,9 |  | pfam08869 | B | 43 |
| hypothetical protein | 71,9 | COG2929 | pfam04365 | B | 33.3 |
| Predicted ATPase | 71,7 | COG4637 | pfam13304 | B/O | 32.3 |
| Acyl-CoA synthetases (AMP-forming)/AMP-acid ligases II | 71,5 | COG0318 | pfam00501 | -^#^ | 51.7 |
| hypothetical protein | 71,4 | - | - | C | 37.5 |
| MoxR-like ATPases | 71,3 | COG0714 | pfam07728 | B | 48 |
| hypothetical protein | 71,3 | - | - | B | 39.4 |
| Rubrerythrin | 71,2 | COG1592 | - | -^#^ | 45.2 |
| sulfotransferase | 71,1 | - | pfam13469 | B | 45.6 |
| 3-hydroxyisobutyrate dehydrogenase | 71 | COG2084 | pfam03466 pfam14833 | C | 42 |
| Sugar-transfer associated ATP-grasp | 70,9 | - | pfam14397 | B | 32.2 |
| Signal transduction histidine kinase | 70,7 | COG0642 | pfam02518 | B | 45.9 |
| hypothetical protein | 70,7 | - | - | B | 33.8 |
| Glycosyltransferases involved in cell wall biogenesis | 70,6 | COG0463 | pfam00535 | B | 44.5 |
| Taurine dioxygenase, alpha-ketoglutarate-dependent | 70,6 | COG2175 | pfam02668 | C/O | 44.6 |
| HAD-superfamily phosphatase, subfamily IIIC/FkbH-like domain-containing protein | 70,5 | - | - | B | 38.7 |
| ABC-type multidrug transport system, ATPase and permease components | 70,5 | COG1132 | pfam00005 | B | 41.1 |
| hypothetical protein | 70,4 | - | - | B | 53.1 |
| tRNA-splicing ligase RtcB | 70,3 | COG1690 | pfam01139 | -^#^ | 48.2 |

! = gene analyse based on IMG entries

¥ = B: *Beggiatoaceae*, C: *Cyanobacteria*, O: other *Bacteria*

* = no flanking genes, target gene show highest similarity to *Beggiatoaceae*

# = no flanking genes, target gene show highest similarity to Cyanobacteria

**Table S4:** Predicted genes and gene products in "*Ca*. T. nelsonii Thio36" and other LSB genomes

| **product** | | | | **gene** | **locus** | | | | | | | | **E.C.** | **AA** | | | | **truncated** | | | | **full** | | | | **other filamentous sulfur oxidizer** | | | | | **comment** | | | |  |  |  |  |
| --- | --- | --- | --- | --- | --- | --- | --- | --- | --- | --- | --- | --- | --- | --- | --- | --- | --- | --- | --- | --- | --- | --- | --- | --- | --- | --- | --- | --- | --- | --- | --- | --- | --- | --- | --- | --- | --- | --- |
| **glycolysis** | | | | | | | | | | | | | | | | | | | | | | | | | | | | | | | | | | |  |  |  |  |
| glucokinase | | | | glk |  | | | | | | | | 2.7.1.2 |  | | | |  | | | |  | | | | BEGALDRAFT_RS17080 BGP_0972 **FLOR_02979** THII_0184 | | | | |  | | | |  |  |  |  |
| polyphosphate glucokinase | | | | ppgk | **THIOM _000321** | | | | | | | | 2.7.1.63 | 120 | | | | **+** | | | |  | | | | BEGALDRAFT_RS00195 BGP_0205 BOGUAY_0012 *Ga0063879_00821*  THII_0714 | | | | |  | | | |  |  |  |  |
| glucose-6-phosphate isomerase | | | | pgi |  | | | | | | | | 5.3.1.9 |  | | | |  | | | |  | | | | BEGALDRAFT_RS07135 BOGUAY_0259 BGP_3455 **FLOR_03085 FLOR_03544**  *Ga0097846_118065**  THII_1441 | | | | | * Illumina metagenome | | | |  |  |  |  |
| 6-phosphofructokinase, pyrophosphate dependent | | | | pfk | **THIOM _005101** | | | | | | | | 2.7.1.11 | 259 | | | | + | | | |  | | | | BEGALDRAFT_RS12370 BOGUAY_3135 BOGUAY_1318 BGP_4425  **FLOR_02042**  *Ga0063879_01082* *Ga0063879_04320*  THII_0379 | | | | |  | | | |  |  |  |  |
| bisphosphate aldolase A | | | | fbaA |  | | | | | | | | 4.1.2.13 |  | | | |  | | | |  | | | | BEGALDRAFT_RS05825 BOGUAY_1000 BGP_5969  **FLOR_00835**  *Ga0063879_04698* THII_3338 | | | | |  | | | |  |  |  |  |
| bisphosphate aldolase B | | | | fbaB | **THIOM _003342** | | | | | | | | 4.1.2.13 | 200 | | | | + | | | |  | | | | *Ga0063879_02057* | | | | |  | | | |  |  |  |  |
| glyceraldehyde-3-phosphate dehydrogenase | | | | gap | **THIOM _000208** | | | | | | | | 1.2.1.12 | 225 | | | | + | | | |  | | | | BEGALDRAFT_RS05930 BOGUAY_2720 BGP_2190 BGP_4586 **FLOR_03217** *Ga0063879_03105* Ga0063879_03594 Ga0063879_07486* THII_0784 | | | | | *below cut off 10^e-5^ | | | |  |  |  |  |
| phosphoglycerate kinase | | | | pgk | **THIOM _004143 THIOM _004791** | | | | | | | | 2.7.2.3 | 138 221 | | | | + | | | |  | | | | BEGALDRAFT_RS02990 BOGUAY_0998 BOGUAY_0999 BGP_4102  **FLOR_00204**  *Ga0063879_04696* THII_3340 | | | | |  | | | |  |  |  |  |
| phosphoglycerate mutase | | | | gpmA | **THIOM_003345** | | | | | | | | 5.4.2.1 | 248 | | | |  | | | | + | | | | BEGALDRAFT_RS08705 BEGALDRAFT_RS15270 BOGUAY_0714 BOGUAY_1652 BGP_0770 BGP_0964 BGP_2533 **FLOR_00744 FLOR_01466 FLOR_02612**  *Ga0063879_00093 Ga0063879_02285* THII_3736 THII_3779 | | | | |  | | | |  |  |  |  |
| enolase | | | | eno | **THIOM_001447 THIOM_003915** | | | | | | | | 4.2.1.11 | 64 171 | | | | + | | | |  | | | | BA09_212 BOGUAY_3393  **FLOR_02427**   *Ga0063879_03742 Ga0063879_07514* THII_3630 | | | | |  | | | |  |  |  |  |
| pyruvate kinase | | | | pyk | **THIOM_004144** | | | | | | | | 2.7.1.40 | 478 | | | |  | | | | + | | | | BEGALDRAFT_RS05830 BGP_5856 **FLOR_00836**   *Ga0063879_04697* THII_3339 | | | | |  | | | |  |  |  |  |
| pyruvate dehydrogenase E1 component | | | | aceE | **THIOM_004182** | | | | | | | | 1.2.4.1 | 863 | | | | + | | | |  | | | | BEGALDRAFT_RS04870 BGP2645 **FLOR_02028** *Ga0063879_00116*  THII_3499 | | | | |  | | | |  |  |  |  |
| dihydrolipoamide acetyltransferase E2 component | | | | aceF | **THIOM_004843** | | | | | | | | 2.3.1.12 | 357 | | | | + | | | |  | | | | BEGALDRAFT_RS04865 BGP_2646  **FLOR_02029**   *Ga0063879_00115* THII_3498 | | | | |  | | | |  |  |  |  |
| pyruvate dehydrogenase E1 component, alpha subunit | | | | pdhA | **THIOM_003598** | | | | | | | | 1.2.4.1 | 158 | | | | + | | | |  | | | | BEGALDRAFT_RS04865 BOGUAY_4507 BOGUAY_4468 BOGUAY_4738 BGP_1305  *Ga0063879_07436* | | | | |  | | | |  |  |  |  |
| pyruvate dehydrogenase E1 component, beta subunit | | | | pdhB | **THIOM_003549** | | | | | | | | 1.2.4.1 | 190 | | | | + | | | |  | | | | BOGUAY_4506 BOGUAY_4469 BGP_1304  *Ga0063879_07435* | | | | |  | | | |  |  |  |  |
| dihydrolipoamide acetyltransferase E2 component | | | | pdhC | **THIOM_004415** | | | | | | | | 2.3.1.12 | 266 | | | | + | | | |  | | | |  | | | | |  | | | |  |  |  |  |
| dihydrolipoamide dehydrogenase E3 | | | | pdhD/lpd | **THIOM_002712 THIOM_002797 THIOM_004445** | | | | | | | | 1.8.1.4 | 234 216 471 | | | | + | | | |  | | | | BEGALDRAFT_RS15655 BEGALDRAFT_RS00680 BOGUAY_0563 BOGUAY_0328 BGP_0643 BGP_0644 BGP_4455  **FLOR_01022 FLOR_03223** *Ga0063879_02623 Ga0063879_04344 Ga0063879_07439* | | | | |  | | | |  |  |  |  |
| **tricarboxylic acid cycle** | | | | | | | | | | | | | | | | | | | | | | | | | | | | | | | | | | |  |  |  |  |
| citrate synthase (type I and II) | | | | gltA | **THIOM_002591** | | | | | | | | 2.3.3.1 | 255 | | | | + | | | |  | | | | BOGUAY_4141 (type I) BGP_3693 (type II) BGP4913 (type II)  **FLOR02625 (type II)** BA15_114 (type II)  *Ga0063879_04165* (type II)*  *Ga0063879_04167* (type II)* THII_3783 (type I) | | | | | * interrupted by transposon | | | |  |  |  |  |
| aconitate hydratase I and II | | | | acnA/ acnB | **THIOM_005461** | | | | | | | | 4.2.1.3 | 715 | | | | + | | | |  | | | | BEGALDRAFT_RS05000 (acnB) BEGALDRAFT_RS16335 (acnA) BOGUAY_3060 (acnB) BGP4006 (acnB) **FLOR_01010 (acnB) FLOR_02559 (acnA)**   *Ga0063879_06333* (acnB) THII_1862 (acnB) | | | | |  | | | |  |  |  |  |
| isocitrate dehydrogenase | | | | icd | **THIOM_005294** | | | | | | | | 1.1.1.42 | 227 | | | | + | | | |  | | | | BEGALDRAFT_RS09420 BOGUAY_2441 **FLOR_01072** *Ga0063879_05535*  THII_3572 | | | | |  | | | |  |  |  |  |
| 2-oxoglutarate dehydrogenase E1 | | | | sucA | **THIOM_001599 THIOM_003777 THIOM_004548** | | | | | | | | 1.2.4.2 | 360 178 213 | | | | + | | | |  | | | | BEGALDRAFT_RS15640 BGP_4352  **FLOR_01020**  *Ga0097846_111266-9** THII_3445 | | | | | *multiple gene disruption and Illumina metagenome | | | |  |  |  |  |
| dihydrolipoyl succinyl transferase E2 | | | | sucB | **THIOM_004415** | | | | | | | | 2.3.1.61 | 266 | | | | + | | | |  | | | | BEGALDRAFT_RS15650 BGP_4350  **FLOR_01021 FLOR_02029**  *Ga0097846_124812**  THII_3444 | | | | | *Illumina metagenome | | | |  |  |  |  |
| succinyl-CoA synthetase, beta subunit | | | | sucC | **THIOM_003650** | | | | | | | | 6.2.1.5 | 283 | | | | + | | | |  | | | | BEGALDRAFT_RS06010 BGP_0418 BOGUAY_1108 **FLOR_00929**   *Ga0063879_02707*  THII_3362 | | | | |  | | | |  |  |  |  |
| Succinyl-CoA synthetase, alpha subunit | | | | sucD | **THIOM_001993** | | | | | | | | 6.2.1.5 | 116 | | | | + | | | |  | | | | BEGALDRAFT_RS06015 BGP_0419 BOGUAY_1109  **FLOR_00930**   *Ga0063879_02708* THII_3361 | | | | |  | | | |  |  |  |  |
| succinate dehydrogenase/ fumarate reductase, flavoprotein subunit | | | | sdhA | **THIOM_000447 THIOM_001443 THIOM_003077** | | | | | | | | 1.3.5.1 | 353 249 69 | | | | + | | | |  | | | | BEGALDRAFT_RS11080 BOGUAY_0963 BGP_0843 BGP_0844 BGP_2544  **FLOR_03153**  *Ga0063879_01113 Ga0063879_02461 Ga0063879_03567* Ga0063879_05611*  THII_0431 | | | | | *below cut off 10e-5 | | | |  |  |  |  |
| succinate dehydrogenase/ fumarate reductase, iron-sulfur subunit | | | | sdhB | **THIOM_000277 THIOM_004151** | | | | | | | | 1.3.5.1 | 143 59 | | | | + | | | |  | | | | BEGALDRAFT_RS11085 BOGUAY_0707 BGP_1918 BGP_3026 BGP_5022  **FLOR_03154**   *Ga0063879_03568*   *Ga0063879_03582 Ga0063879_06069*  THII_0430 | | | | |  | | | |  |  |  |  |
| succinate dehydrogenase/ fumarate reductase, cytochrome b556 subunit | | | | sdhC | **THIOM_004152** | | | | | | | | 1.3.5.1 | 293 | | | | + | | | |  | | | | BEGALDRAFT_RS11065 BOGUAY_4609 BGP_2542 BGP_3027 BGP_3589 BGP_1067  **FLOR_03151**  *Ga0063879_02459 Ga0063879_03569 Ga0063879_05185*  THII_0433 | | | | |  | | | |  |  |  |  |
| succinate dehydrogenase/ fumarate reductase, membrane anchor | | | | sdhD |  | | | | | | | | 1.3.5.1 |  | | | |  | | | |  | | | | BEGALDRAFT_RS11070 BGP_2543 **FLOR_03152**  *Ga0063879_02460*  THII_0432 | | | | |  | | | |  |  |  |  |
| Fumarate hydratase | | | | fumAB | **THIOM_000923** | | | | | | | | 4.2.1.2 | 505 | | | |  | | | | + | | | | BEGALDRAFT_RS05310 BOGUAY_1148 BGP_4244  **FLOR_02976**   *Ga0063879_05135*  THII_3214 | | | | |  | | | |  |  |  |  |
| malate dehydrogenase | | | | malate | **THIOM_004470** | | | | | | | | 1.1.1.37 | 325 | | | |  | | | | + | | | | BEGALDRAFT_RS15640 BOGUAY_0329 **FLOR_03183**   *Ga0097846_111265** THII_3446 | | | | | * Illumina metagenome | | | |  |  |  |  |
| **reductive tricarbolxyic acid cycle** | | | | | | | | | | | | | | | | | | | | | | | | | | | | | | | | | | |  |  |  |  |
| ATP_citrate lyase | | | | aclA | **THIOM_004313** | | | | | | | | 2.3.8.8 | 422 | | | |  | | | | + | | | | BOGUAY_3507 BOGUAY_3508 *Ga0063879_05528 Ga0063879_05529* | | | | |  | | | |  |  |  |  |
| ATP_citrate lyase | | | | aclB |  | | | | | | | |  |  | | | |  | | | |  | | | |  | | | | |  | | | |  |  |  |  |
| citryl-CoA lyase, alpha subunit | | | | citF |  | | | | | | | |  |  | | | |  | | | |  | | | |  | | | | |  | | | |  |  |  |  |
| citryl-CoA lyase, beta subunit | | | | citE |  | | | | | | | |  |  | | | |  | | | |  | | | | BEGALDRAFT_RS08255  **FLOR_02468** | | | | |  | | | |  |  |  |  |
| fumarate reductase, flavocytochrome C | | | | frdA | **THIOM_000276 THIOM_001017 THIOM_005649** | | | | | | | | 1.33.9.1 | 219 140 434 | | | | + | | | |  | | | | BOGUAY_3541 BGP_2143 *Ga0097846_100921** | | | | | * Illumina metagenome | | | |  |  |  |  |
| fumarate reductase, iron-sulfur protein | | | | frdB |  | | | | | | | | 1.33.9.1 |  | | | |  | | | |  | | | |  | | | | |  | | | |  |  |  |  |
| 2-oxoglutarate:ferredoxin oxidoreductase, alpha subunit | | | | korA | **THIOM_000752** | | | | | | | | 1.2.7.3 | 377 | | | |  | | | | + | | | | BOGUAY_1065 BGP_0270  *Ga0063879_04151* | | | | |  | | | |  |  |  |  |
| 2-oxoglutarate :ferredoxin oxidoreductase, beta subunit | | | | korB | **THIOM_002126** | | | | | | | | 1.2.7.3 | 191 | | | |  | | | | + | | | | BOGUAY_1064  *Ga0063879_04150* | | | | |  | | | |  |  |  |  |
| 2-oxoglutarate :ferredoxin oxidoreductase, gamma subunit | | | | korG |  | | | | | | | |  |  | | | |  | | | |  | | | |  | | | | |  | | | |  |  |  |  |
| 2-oxoglutarate :ferredoxin oxidoreductase, delta subunit | | | | korD |  | | | | | | | |  |  | | | |  | | | |  | | | |  | | | | |  | | | |  |  |  |  |
| pyruvate:ferredoxin oxidoreductase, alpha subunit | | | | porA | **THIOM_000425 THIOM_001671 THIOM_004200 THIOM_004981** | | | | | | | | 1.2.7.1 | 505 753 388 309 | | | | + | | | | + | | | | BOGUAY_4121 BGP_3761  **FLOR_00935**  *Ga0063879_05883 Ga0097846_100432* Ga0097846_104692* Ga0097846_121953* Ga0097846_124202** | | | | | * Illumina metagenome | | | |  |  |  |  |
| pyruvate:ferredoxin oxidoreductase, beta subunit | | | | porB | **THIOM_004201** | | | | | | | | 1.2.7.1 | 345 | | | |  | | | | + | | | | BGP_5558 *Ga0097846_124203** | | | | | * Illumina metagenome | | | |  |  |  |  |
| pyruvate:ferredoxin oxidoreductase, gamma subunit | | | | porG | **THIOM_001670** | | | | | | | | 1.2.7.1 | 118 | | | | + | | | |  | | | | BOGUAY_4123 BGP_2254  **FLOR_00934**  *Ga0097846_100431* Ga0097846_104693* Ga0097846_124201** | | | | | * Illumina metagenome | | | |  |  |  |  |
| pyruvate:ferredoxin oxidoreductase, delta subunit | | | | porD | **THIOM_004199** | | | | | | | | 1.2.7.1 | 131 | | | | + | | | |  | | | | BOGUAY_4122  *Ga0097846_121952** | | | | | * Illumina metagenome | | | |  |  |  |  |
| pyruvate flavodoxin/ ferredoxin oxidoreductase | | | | porACDB ("nifJ) | **THIOM_000261 THIOM_002577 THIOM_002835 THIOM_005289** | | | | | | | | 1.2.7. | 1262 72 79 385 | | | | + | | | |  | | | | BOGUAY_0108 BOGUAY_2919  *Ga0063879_05883* | | | | |  | | | |  |  |  |  |
| acetyl-CoA syntethase | | | | acs | **THIOM_005698** | | | | | | | | 6.2.1.1 | 223 | | | | + | | | |  | | | | BEGALDRAFT_RS01260 BGP_6368  **FLOR_00817**  *Ga0063879_01856* | | | | |  | | | |  |  |  |  |
| pyruvate phosphate dikinase | | | | ppdK | **THIOM_002229 THIOM_002395 THIOM_002806** | | | | | | | | 2.7.9.1 | 202 523 225 | | | | + | | | |  | | | | BEGALDRAFT_RS07715 BGP _3309 BGP_3310  **FLOR_02175**  BOGUAY_5237 *Ga0063879_01165 Ga0063879_03104* | | | | |  | | | |  |  |  |  |
| phosphoenolpyruvate synthase | | | | ppsA | **THIOM_000011 THIOM_003239 THIOM_005419** | | | | | | | | 2.7.9.2 | 207 281 631 | | | | + | | | |  | | | | BEGALDRAFT_RS17560 **FLOR_02068**  *Ga0063879_04114* | | | | |  | | | |  |  |  |  |
| phosphoenolpyruvate carboxylase /ATP or GTP dependent cabroxykinase | | | | pckA/ pckG/ ppc | **THIOM_002640 (pckA ATP)** | | | | | | | | 4.1.1.31/  4.1.1.32/ 4.1.1.49 | 158 | | | | + | | | |  | | | | BEGALDRAFT_RS05450 (pckA ATP) BGP_5540 (pckA ATP)  **FLOR_01047 (pckA ATP)**  BGP_2644 (pckG GTP) BEGALDRAFT_RS13455 (ppc) **FLOR_00831 (ppc)**  *Ga0063879_00207* (pckA ATP) | | | | |  | | | |  |  |  |  |
| **Calvin-Benson-Bassham cycle** | | | | | | | | | | | | | | | | | | | | | | | | | | | | | | | | | | |  |  |  |  |
| ribulose bisphosphate carboxylase large chain, form I | | | | rbcL | **THIOM_002425 THIOM_001920 THIOM_001493** | | | | | | | | 4.1.1.39 | 53 319 136 | | | | + | | | |  | | | | BEGALDRAFT_RS02035 BGP_3377 | | | | |  | | | |  |  |  |  |
| ribulose bisphosphate carboxylase large chain, form II | | | | rbcM |  | | | | | | | | 4.1.1.39 |  | | | |  | | | |  | | | | BOGUAY_1665  **FLOR01412**  *Ga0063879_01561* | | | | |  | | | |  |  |  |  |
| ribulose bisphosphate carboxylase small subunit | | | | rbcS | **THIOM_002424** | | | | | | | | 4.1.1.39 | 39 | | | | + | | | |  | | | | BEGALDRAFT_RS02030 BGP_3376 | | | | |  | | | |  |  |  |  |
| phosphoglycerate kinase | | | | pgk | **THIOM_004143 THIOM_004791** | | | | | | | | 2.7.2.3 | 138 221 | | | | + | | | |  | | | | BEGALDRAFT_RS02990 BOGUAY_0998 BOGUAY_0999 BGP_4102  **FLOR_4102**  *Ga0063879_04696* | | | | |  | | | |  |  |  |  |
| fructose-1,6-bisphosphatase | | | | fbp |  | | | | | | | | 3.1.3.11 |  | | | |  | | | |  | | | | BEGALDRAFT_RS00605  **FLOR_00141** | | | | |  | | | |  |  |  |  |
| transketolase | | | | tkt | **THIOM_000659 THIOM_000660 THIOM_000719** | | | | | | | | 2.2.1.1 | 112 106 389 | | | | + | | | |  | | | | BEGALDRAFT_RS05920 BGP_0483 BGP_2087 BGP_2695 BOGUAY_2721  **FLOR_02879**  *Ga0063879_03595* | | | | |  | | | |  |  |  |  |
| pyrphosphate dependent 6-phosphofructokinase | | | | ppi-PFK | **THIOM_005101** | | | | | | | | 2.7.1.90 | 259 | | | | + | | | |  | | | | BEGALDRAFT_RS12370 BOGUAY_3135 BOGUAY_1318 BGP_4425  **FLOR_02042**   *Ga0063879_01082 Ga0063879_04320* | | | | |  | | | |  |  |  |  |
| ribose-5-phosphatisomerase | | | | cbbI |  | | | | | | | | 5.3.1.6 |  | | | |  | | | |  | | | | BEGALDRAFT_RS16745 BOGUAY_3213 BGP_5744  **FLOR00803**  *Ga0063879_07052* | | | | |  | | | |  |  |  |  |
| phosphoribulokinase | | | | prkA | **THIOM_001518** | | | | | | | | 2.7.1.19 | 250 | | | | + | | | |  | | | | BEGALDRAFT_RS00760 Boguay_2689 BGP_2255  **FLOR_02126**   *Ga0063879_06213* | | | | |  | | | |  |  |  |  |
| **C2 cycle (glycolate cycle)** | | | | | | | | | | | | | | | | | | | | | | | | | | | | | | | | | | |  |  |  |  |
| phosphoglycolate phosphatase | | | | gph | **THIOM_002146 THIOM_003811** | | | | | | | | 3.1.3.18 | 216 68 | | | | + | | | |  | | | | BEGALDRAFT_RS11520 BEGALDRAFT_RS04825 BEGALDRAFT_RS10980 BOGUAY_4193 BOGUAY_0641 BOGUAY_0964 BGP_2114 BGP_4813 BGP_5647 **FLOR_02636 FLOR_02818**   *Ga0063879_00091 Ga0063879_00190 Ga0063879_00335*  THII_RS18350 | | | | |  | | | |  |  |  |  |
| glycolate oxidase | | | | glcD | **THIOM_000485 THIOM_001098** | | | | | | | | 1.1.3.15 | 429 441 | | | | + | | | |  | | | | BEGALDRAFT_RS01860 BGP_2712  **FLOR_03339 FLOR_03342**  *Ga0063879_04338* | | | | |  | | | |  |  |  |  |
| glycolate oxidase, FAD-binding subunit | | | | glcE | **THIOM_001099** | | | | | | | | 1.1.3.15 | 347 | | | | + | | | |  | | | | BEGALDRAFT_RS01855 BGP_4161  **FLOR_02865**   *Ga0063879_04337* | | | | |  | | | |  |  |  |  |
| glycolate oxidase, iron-sulfur subunit | | | | glcF | **THIOM_000870 THIOM_001962 THIOM_004994** | | | | | | | | 1.1.3.15 | 212 289 537 | | | | + | | | |  | | | | BEGALDRAFT_RS05135 BGP_4162 **FLOR_01250**   *Ga0063879_04336* | | | | |  | | | |  |  |  |  |
| glutamate-glyoxylate aminotransferase | | | | GGAT | **THIOM_001040** | | | | | | | | 2.6.1. | 64 | | | |  | | | |  | | | | BEGALDRAFT_RS01160 BEGALDRAFT_RS06465 BOGUAY_1325 BGP_1896 BGP_1897 **FLOR_00764 FLOR_02517**   *Ga0063879_01791* | | | | |  | | | |  |  |  |  |
| glycine cleavage system, glycine dehydrogenase, P-protein | | | | gcvPA |  | | | | | | | | 1.4.4.2 |  | | | |  | | | |  | | | | BEGALDRAFT_RS01735 | | | | |  | | | |  |  |  |  |
| glycine cleavage system, glycine dehydrogenase, P-protein | | | | gcvPB | **THIOM_005742 (gcvB)** | | | | | | | | 1.4.4.2 | 456 | | | |  | | | | + | | | | BEGALDRAFT_RS13530 BOGUAY_1468 BOGUAY_3307 BGP_1733 BGP_1734  BGP_6215  **FLOR_02629 FLOR_03006 FLOR_02133**  *Ga0063879_04160 Ga0063879_06517* | | | | |  | | | |  |  |  |  |
| glycine cleavage system, aminomethyltransferase, T-protein | | | | gcvT | **THIOM_002333** | | | | | | | | 2.1.2.10 | 243 | | | | + | | | |  | | | | BEGALDRAFT_RS01725 BA19_184 BOGUAY_3309 BGP_1517  **FLOR_01593 FLOR_02629**   *Ga0063879_06519* | | | | |  | | | |  |  |  |  |
| glycine cleavage system, dihydrolipoyl dehydrogenase, L-protein | | | | gcvL | **THIOM_002712 THIOM_002797 THIOM_004445** | | | | | | | | 1.8.1.4 | 216 234 471 | | | |  | | | | + | | | | BEGALDRAFT_RS01735 BA17_328 BOGUAY_0563 BOGUAY_0328 BGP_0644  **FLOR_01022**  *Ga0063879_02623*  *Ga0063879_07439 Ga0063879_04344* | | | | |  | | | |  |  |  |  |
| glycine cleavage system, H-protein | | | | gcvH |  | | | | | | | | 1.4.4.2 |  | | | |  | | | |  | | | | BEGALDRAFT_RS01730 BOGUAY_3308 BGP_5185  **FLOR_01150**  *Ga0063879_06518* | | | | |  | | | |  |  |  |  |
| glycine cleavage transcriptional repressor | | | | gcvR |  | | | | | | | |  |  | | | |  | | | |  | | | | BEGALDRAFT_RS02315 **FLOR_01593** | | | | |  | | | |  |  |  |  |
| serine/ glycine hydroxymethyltransferase | | | | glyA | **THIOM_004617 THIOM_001326** | | | | | | | | 2.1.2.1 | 341 152 | | | | + | | | |  | | | | BEGALDRAFT_RS01435 BOGUAY_1680 BGP_4519 **FLOR_00302**  *Ga0063879_04044* | | | | |  | | | |  |  |  |  |
| serine-glyoxylate aminotransferase | | | | SGAT | **THIOM_005651** | | | | | | | | 2.6.1.51 | 107 | | | | + | | | |  | | | | BGP_3194 BGP_5274 *Ga0063879_03708* | | | | |  | | | |  |  |  |  |
| serine/ threonine protein kinase | | | | STPK | **THIOM_000912 THIOM_001549 THIOM_001868 THIOM_005022** | | | | | | | | 2.7.11.1 | 429 113 550 380 | | | | + | | | |  | | | | BEGALDRAFT_RS18320 BEGALDRAFT_RS18365 BGP_3584 BGP_4568 **FLOR_01423 FLOR_03350 FLOR_03427**  *Ga0063879_00360* | | | | | we detected much more genes encoding for STPK, which are not shown here | | | |  |  |  |  |
| phosphoserine phosphatase | | | | serB | **THIOM_003438** | | | | | | | | 3.1.3.3 | 194 | | | | + | | | |  | | | | BEGALDRAFT_RS06875 | | | | | bifunctional enzyme with EC 2.7.11.1 | | | |  |  |  |  |
| phosphoserine aminotransferase | | | | serC | **THIOM_000630** | | | | | | | | 2.6.1.52 | 450 | | | | + | | | |  | | | | BEGALDRAFT_RS06875 BOGUAY_0357 BGP_2880 BGP_2994 **FLOR_03048**  *Ga0063879_00888* | | | | |  | | | |  |  |  |  |
| D-3-phosphoglycerate dehydrogenase | | | | serA | **THIOM_000598 THIOM_004053 THIOM_004832** | | | | | | | | 1.1.1.95 | 255 127 304 | | | | + | | | |  | | | | BGP_2879 BGP_5532  *Ga0063879_00887* | | | | |  | | | |  |  |  |  |
| glycerate dehydrogenase | | | | dhg | **THIOM_004025** | | | | | | | | 1.1.1.29 | 254 | | | | + | | | |  | | | | *Ga0097846_123721** | | | | | same function as hydroxy- pyruvate reductase, * Illumina metagenome | | | |  |  |  |  |
| hydroxypyruvate reductase | | | | ttuD | **THIOM_005204** | | | | | | | | 1.1.1.81 | 212 | | | | + | | | |  | | | | BOGUAY_4043 BGP_3955  **FLOR_01443**   *Ga0063879_01815* | | | | |  | | | |  |  |  |  |
| glycerate kinase | | | | GLCK |  | | | | | | | |  |  | | | |  | | | |  | | | |  | | | | |  | | | |  |  |  |  |
| **Membrane-bound cytoplasmic nitrate reductase** | | | | | | | | | | | | | | | | | | | | | | | | | | | | | | | | | | | |  |  |  |
| nitrate reductase, alpha subunit | narG | | | | | **THIOM_002225 THIOM_004150** | | | | | 1.7.99.4 | | | | 261 31 | | | | + | | | |  | | | | BOGUAY_0051 BOGUAY_0489 BGP_0139 BGP_3372 BGP_5024 *Ga0063879_01731 Ga0063879_03576*  THII_1673 | | | | |  | | | |  |  |  |
| nitrate reductase, beta subunit | narH | | | | | **THIOM_004149 THIOM_005096** | | | | | 1.7.99.4 | | | | 497 394 | | | | + | | | | + | | | | BOGUAY_0490 BOGUAY_0049 BGP_4035 BGP_4784 *Ga0063879_01732 Ga0063879_03575*  THII_1674 | | | | |  | | | |  |  |  |
| nitrate reductase, delta subunit | narJ | | | | | **THIOM_004148** | | | | | 1.7.99.4 | | | | 173 | | | |  | | | | + | | | | BOGUAY_0491 BGP_4033  *Ga0063879_01733*  THII_1676 | | | | | molybdenum cofactor assembly chaperone | | | |  |  |  |
| nitrate reductase, gamma subunit | narI | | | | | **THIOM_004147** | | | | | 1.7.99.4 | | | | 75 | | | | + | | | |  | | | | BEGALDRAFT_RS06745 BOGUAY_0492 BOGUAY_1505 BGP_2700  *Ga0063879_01734 Ga0063879_03573*  THII_1675 | | | | |  | | | |  |  |  |
| nitrate reductase-like protein | narX | | | | | **THIOM_005227 THIOM_005605** | | | | | 1.7.99.4 | | | | 259 552 | | | | + | | | |  | | | | BOGUAY_0048 BOGUY_0146 | | | | |  | | | |  |  |  |
| nitrate/ nitrite transporter | narK | | | | | **THIOM_003288** | | | | |  | | | | 204 | | | | + | | | |  | | | | BEG_3802  *Ga0063879_02276* THII_3146 | | | | |  | | | |  |  |  |
| **Periplasmic nitrate reductase** | | | | | | | | | | | | | | | | | | | | | | | | | | | | | | | | | | | |  |  |  |
| nitrate reductase, periplasmatic, large subunit | napA | | | | | **THIOM_001051 THIOM_000365** | | | | | 1.7.99.4 | | | | 835 417 | | | | + | | | | + | | | | BEGALDRAFT_RS11680 BOGUAY_0671 BGP_1198 *Ga0063879_03474* | | | | |  | | | |  |  |  |
| nitrate reductase, small subunit | napB | | | | | **THIOM_003084** | | | | | 1.7.99.4 | | | | 38 | | | | + | | | |  | | | | BEGALDRAFT_RS11695 BOGUAY_0672 BGP_1425  *Ga0063879_03467* | | | | |  | | | |  |  |  |
| nitrate reductase cytochrome c-type protein | napC | | | | |  | | | | | 1.7.99.4 | | | |  | | | |  | | | |  | | | | BEGALDRAFT_RS11700 BOGUAY_3233 BGP_1197 FLOR_01446 *Ga0063879_03466* | | | | |  | | | |  |  |  |
| nitrate reductase, ferredoxin-type potein | napH | | | | | **THIOM_002746** | | | | | 1.7.99.4 | | | | 315 | | | | + | | | |  | | | | BEGALDRAFT_RS11690 *Ga0063879_03468** THII_2890 | | | | | * disrupted by Group II intron and accessory genes | | | |  |  |  |
| nitrate reductase, ferredoxin-type potein | napG | | | | | **THIOM_001050 THIOM_004442** | | | | | 1.7.99.4 | | | | 254 247 | | | |  | | | | + | | | | BEGALDRAFT_RS11685  *Ga0063879_03473*  THII_2889 | | | | |  | | | |  |  |  |
| nitrate reductase, periplasmatic | napD | | | | |  | | | | | 1.7.99.4 | | | |  | | | |  | | | |  | | | | BEGALDRAFT_RS11675 BGP_1199  *Ga0063879_03475* | | | | |  | | | |  |  |  |
| nitrate reductase, ferredoxin-type potein | napF | | | | |  | | | | | 1.7.99.4 | | | |  | | | |  | | | |  | | | | BEGALDRAFT_RS11670 BOGUAZ_5179 BGP_1200  *Ga0063879_03478* | | | | |  | | | |  |  |  |
| nitrate reductase, large subunit | nasA | | | | | **THIOM_001947** | | | | | 1.7.99.4 | | | | 285 | | | | + | | | |  | | | | BEGALDRAFT_RS06820 *Ga0063879_05305*  THII_1778 | | | | | assimilatory nitrate reduction to ammonia | | | |  |  |  |
| nitrite reductase, NAD(P)H large subunit | nirB | | | | | **THIOM_000288 THIOM_000287 THIOM_001106** | | | | | 1.7.1.4 | | | | 216 232 407 | | | | + | | | |  | | | | BEGALDRAFT_RS06830  *Ga0063879_01287*  THII_1782 | | | | | dissimilatory nitrate reduction to ammonia as well as assimilatory | | | |  |  |  |
| nitrite reductase, NAD(P)H small subunit | nirD | | | | |  | | | | | 1.7.1.4 | | | |  | | | |  | | | |  | | | | BEGALDRAFT_RS06825 *Ga0063879_01288*  THII_1781 | | | | | dissimilatory nitrate reduction to ammonia as well as assimilatory | | | |  |  |  |
| nitrite reductase, ferredoxin | nirA | | | | |  | | | | | 1.7.7.1 | | | |  | | | |  | | | |  | | | | FLOR_00816 | | | | | dissimilatory nitrate reduction to ammonia as well as assimilatory | | | |  |  |  |
| nitrite reductase cytochrome cd1 | nirS | | | | | **THIOM_002544** | | | | | 1.7.2.1 1.7.99.1 | | | | 591 | | | |  | | | | + | | | | BGP_1272 BOGUAY_2967 *Ga0063879_04847*  THII_2875 | | | | | involved in denitrification | | | |  |  |  |
| nitrite reductase, cytochrome c55X | nirC | | | | | **THIOM_002543** | | | | | 1.7.2.1 1.7.99.1 | | | | 107 | | | | + | | | |  | | | | BGP_1371  *Ga0063879_04851* THII_2876 | | | | |  | | | |  |  |  |
| nitrite reductase, cytochrome cd1 | nirF | | | | | **THIOM_002542 THIOM_003314** | | | | | 1.7.2.1 1.7.99.1 | | | | 305 39 | | | | + | | | |  | | | | BGP_1372 *Ga0063879_04852* THII_2877 | | | | |  | | | |  |  |  |
| transcriptional regulator | nirD | | | | |  | | | | | 1.7.2.1 1.7.99.1 | | | |  | | | |  | | | |  | | | | *Ga0063879_01288*  THII_2878 | | | | |  | | | |  |  |  |
| nitrite reductase heme biosynthesis L protein | nirL | | | | |  | | | | | 1.7.2.1 1.7.99.0 | | | |  | | | |  | | | |  | | | | THII_2879 | | | | |  | | | |  |  |  |
| nitrite reductase heme biosynthesis G protein | nirG | | | | | **THIOM_004400** | | | | | 1.7.2.1 1.7.99.1 | | | | 147 | | | |  | | | | + | | | | BGP_1275 THII_2880 | | | | |  | | | |  |  |  |
| nitrite reductase heme biosynthesis H protein | nirH | | | | |  | | | | | 1.7.2.1 1.7.99.1 | | | |  | | | |  | | | |  | | | | BGP_4270 THII_2881 | | | | |  | | | |  |  |  |
| nitrite reductase heme biosynthesis J protein | nirJ | | | | | **THIOM_001963** | | | | | 1.7.2.1 1.7.99.1 | | | | 261 | | | |  | | | | + | | | | BGP_3921 BGP_4010 *Ga0063879_04857* THII_2882 | | | | |  | | | |  |  |  |
| nitrite reductase heme biosynthesis E protein | nirE | | | | |  | | | | | 1.7.2.1 1.7.99.1 | | | |  | | | |  | | | |  | | | |  | | | | |  | | | |  |  |  |
| nitrite reductase heme biosynthesis N protein | nirN | | | | | **THIOM_005225** | | | | | 1.7.2.1 1.7.99.1 | | | | 94 | | | | + | | | |  | | | |  | | | | |  | | | |  |  |  |
| nitric oxide reductase | norB | | | | | **THIOM_004355 THIOM_005044** | | | | | 1.7.99.7 | | | | 111 338 | | | |  | | | | + | | | | BOGUAY_0863 BGP_3622 BGP_5178 *Ga0063879_02916*  THII_0335 | | | | |  | | | |  |  |  |
| nitric oxide reductase | norC | | | | | **THIOM_001269** | | | | | 1.7.99.7 | | | | 69 | | | | + | | | |  | | | | BOGUAY_0144 BOGUAY_4015 BGP_5602 *Ga0063879_02915* THII_0334 | | | | |  | | | |  |  |  |
| nitric oxide activation protein | norQ | | | | | **THIOM_004352 THIOM_005043** | | | | | 1.7.99.7 | | | | 267 246 | | | | + | | | | + | | | | BEGALDRAFT_RS13260 BGP_2171 BGP_2329 FLOR_01410 FLOR_02605  *Ga0063879_04308*  THII_0336 | | | | |  | | | |  |  |  |
| nitric oxide activation protein | norD | | | | | **THIOM_002148 THIOM_005335** | | | | | 1.7.99.7 | | | | 644 537 | | | |  | | | | + | | | | BGP_5686 FLOR_00559 FLOR_00821 *Ga0063879_04846*  THII_0339 | | | | |  | | | |  |  |  |
| nitric oxide activation protein | norE | | | | | **THIOM_004354** | | | | | 1.7.99.7 | | | | 192 | | | |  | | | | + | | | |  | | | | |  | | | |  |  |  |
| nitrous oxide reductase | nosZ | | | | | **THIOM_003099** | | | | | 1.7.99.6 | | | | 218 | | | | + | | | |  | | | | *Ga0063879_04858*  THII_2884 | | | | |  | | | |  |  |  |
| nitrous oxide reductase accesor protein | nosD | | | | | **THIOM_004441** | | | | | 1.7.99.6 | | | | 406 | | | |  | | | | + | | | | *Ga0063879_04859* THII_2888 | | | | |  | | | |  |  |  |
| nitric oxide dioxygenase | hmp | | | | | **THIOM_003927 THIOM_004279** | | | | | 1.14.12.17 | | | | 144 149 | | | | + | | | |  | | | | BEGALDRAFT_RS13870 FLOR_02537  *Ga0097846_124443* | | | | |  | | | |  |  |  |
| glutamine synthetase | glnA | | | | | **THIOM_000169** | | | | | 6.3.1.2 | | | | 234 | | | | + | | | |  | | | | BEGALDRAFT_RS01445 BEGALDRAFT_RS14825 BGP_4113 BGP_4114 BOGUAY_0161 FLOR_03203 *Ga0063879_04401 Ga0063879_05330 Ga0063879_06490* THII_0257 | | | | |  | | | |  |  |  |
| ammonium transporter | amt | | | | | **THIOM_004982** | | | | |  | | | | 136 | | | |  | | | |  | | | | BEGALDRAFT_RS13090 BEGALDRAFT_RS02975 BOGUAY_3555 BGP1735 FLOR_02001 FLOR_02712  *Ga0063879_04108 Ga0063879_04110* THII_2433 | | | | |  | | | |  |  |  |
| **Sulfide oxidation** | | | | | | | | | | | | | | | | | | | | | | | | | | | | | | | | | | | |  |  |  |
| sulfide:quinone oxidoreductase | sqr | | | | | **THIOM_001728** | | | | | | 1.8.5.4 | | | 373 | | | | |  | | | + | | | | | BEGALDRAFT_RS13295 BEGALDRAFT_RS00840 BGP_0667 BOGUAY_0181 BOGUAY_2390  **FLOR_01938**   *Ga0063879_06096*  THII_0779 | | | | |  | | |  |  |  |
| flavocytochrome c sulfide dehydrogenase, cytochrome c subunit | fccA | | | | | **THIOM_000624** | | | | | | 1.8.2.3 | | | 218 | | | | | + | | | + | | | | | BEGALDRAFT_RS02555 BGP_4977 BOGUAY_2852 BOGUAY_3988  **FLOR_01512**  *Ga0063879_04660 Ga0063879_00802 Ga0063879_02950 Ga0063879_00367* THII_1692 | | | | |  | | |  |  |  |
| flavocytochrome c sulfide dehydrogenase, flavoprotein subunit | fccB | | | | | **THIOM_000858 THIOM_004002 THIOM_004340 THIOM_004562** | | | | | | 1.8.2.3 | | | 254 425 210 217 | | | | | + | | | + | | | | | BEGALDRAFT_RS02560 BGB_0124 BGP_4976 BOGUAY_2853 BOGUAY_3987  **FLOR_01513**   *Ga0063879_00803 Ga0063879_04659* THII_1691 | | | | |  | | |  |  |  |
| **Sulfur oxidation via reverse dissimilatory sulfite reduction** | | | | | | | | | | | | | | | | | | | | | | | | | | | | | | | | | | | |  |  |  |
| dissimilatory sulfite reductase, alpha subunit | dsrA | | | | |  | | | | | | 1.8.99.1 | | |  | | | | |  | | |  | | | | | BGP_6219 BGP_6220 BGP_6501 BOGUAY_1511  **FLOR_02859 FLOR_01613**   *Ga0063879_00262**   *Ga0063879_00263** THII_0611 | | | | | *disrupted by Group I intron | | |  |  |  |
| dissimilatory sulfite reductase, beta subunit | dsrB | | | | | **THIOM_001520** | | | | | | 1.8.99.1 | | | 209 | | | | | + | | |  | | | | | BGP_4858 BOGUAY_1510 **FLOR_02585**  *Ga0063879_00264*  THII_0612 | | | | |  | | |  |  |  |
| dissimilatory sulfite reductase, gamma subunit | dsrC/ dsrC-like | | | | | **THIOM_001370 THIOM_003868 THIOM_004186 THIOM_002705** | | | | | | 1.8.99.1 | | | 110 117 114 131 | | | | | + | | |  | | | | | BEGALDRAFT_RS06750 BGP_1169 BOGUAY_1506  **FLOR_02854**   *Ga0063879_05685*  THII_1848 | | | | | dsr-like protein no ORFs of dsrC gene | | |  |  |  |
| sulfite reductase subunit E | dsrE | | | | | **THIOM_003401 THIOM_003697** | | | | | |  | | | 36 122 | | | | | + | | |  | | | | | BEGALDRAFT_RS06765 BGP_6597 BOGUAY_1509 **FLOR_02857**  *Ga0063879_00265* THII_0613 | | | | |  | | |  |  |  |
| sulfite reductase subunit F | dsrF | | | | |  | | | | | |  | | |  | | | | |  | | |  | | | | | BEGALDRAFT_RS06760 BGP_1172 BOGUAY_1508 **FLOR_02856**   *Ga0063879_00266* THII_0614 | | | | |  | | |  |  |  |
| sulfite reductase, subunit H | dsrH | | | | |  | | | | | |  | | |  | | | | |  | | |  | | | | | BEGALDRAFT_RS06755 BGP_1170 BOGUAY_1507  **FLOR_02855**  *Ga0063879_05684*  THII_0615 | | | | |  | | |  |  |  |
| membrane-bound b-type cytochrome, subunit M | dsrM | | | | | **THIOM_002150** | | | | | |  | | | 213 | | | | | + | | |  | | | | | BEGALDRAFT_RS06745 BGP_0409 BOGUAY_1505  **FLOR_02853**  *Ga0063879_05686*  THII_0617 | | | | |  | | |  |  |  |
| iron sulfur protein subunit K | dsrK | | | | | **THIOM_002151 THIOM_000341** | | | | | |  | | | 84 44 | | | | | + | | |  | | | | | BGP_4599 BOGUAY_1504  **FLOR_02852**  *Ga0063879_05687* THII_0618 | | | | |  | | |  |  |  |
| NADPH:acceptor oxidoreductase, subunit L | dsrL | | | | | **THIOM_000343** | | | | | |  | | | 238 | | | | | + | | |  | | | | | BGP_4600 BOGUAY3227 **FLOR_02849**  *Ga0063879_00259*  THII_0619 | | | | |  | | |  |  |  |
| triheme c-type cytochrome, subunit J | dsrJ | | | | |  | | | | | |  | | |  | | | | |  | | |  | | | | | BGP_4601 BOGUAY_1503 **FLOR_02848** *Ga0063879_05688*  THII_0620 | | | | |  | | |  |  |  |
| periplasmic iron-sulfur protein, subunit O | dsrO | | | | |  | | | | | |  | | |  | | | | |  | | |  | | | | | BGP_4603 BGP_4604 BOGUAY_1501 **FLOR_02847** *Ga0063879_05690*  THII_0621 | | | | |  | | |  |  |  |
| integral membrane protein, subunit P | dsrP | | | | |  | | | | | |  | | |  | | | | |  | | |  | | | | | BGP_4605 BOGUAY_1500  **FLOR_02846**  *Ga0063879_05691*  THII_0622 | | | | |  | | |  |  |  |
| siro(haem)amidase, subunit N | dsrN | | | | |  | | | | | |  | | |  | | | | |  | | |  | | | | | BGP_5248  **FLOR_00351** *Ga0063879_05694* | | | | |  | | |  |  |  |
| sulfite reductase, subunit S | dsrS | | | | | **THIOM_002193** | | | | | |  | | | 138 | | | | | + | | |  | | | | | BGP_1012  **FLOR_02633** *Ga0063879_01120*  THII_1535 | | | | |  | | |  |  |  |
| sulfite reductase, subunit R | dsrR | | | | |  | | | | | |  | | |  | | | | |  | | |  | | | | | BGP_1732 **FLOR_00352**   *Ga0063879_01854*  THII_3808 | | | | |  | | |  |  |  |
| **Sulfite oxidation** | | | | | | | | | | | | | | | | | | | | | | | | | | | | | | | | | | | |  |  |  |
| APS reductase, alpha subunit | aprA | | | | | **THIOM_003261** | | | | | | 1.8.99.2 | | | 104 | | | | | + | | |  | | | | | BGP_5623 BGP_5624 BOGUAY_2553  *Ga0063879_05084* THII_3106 | | | | |  | | |  |  |  |
| APS reductase, beta subunit | aprB | | | | |  | | | | | | 1.8.99.2 | | |  | | | | |  | | |  | | | | | BGP_5858 BOGUAY_2554 *Ga0063879_05084* THII_3105 | | | | |  | | |  |  |  |
| sulfate adenylyltransferase | sat | | | | | **THIOM_002956** | | | | | | 2.7.7.4 | | | 35 | | | | | + | | |  | | | | | BGP_6163 BOGUAY_2370  **FLOR_01554**   *Ga0063879_04214* THII_1057 | | | | |  | | |  |  |  |
| alkaline serine protease | aprM | | | | | **THIOM_003459** | | | | | | 3.4.21.- | | | 195 | | | | | + | | |  | | | | |  | | | | |  | | |  |  |  |
| **SOX system** | | | | | | | | | | | | | | | | | | | | | | | | | | | | | | | | | | | |  |  |  |
| SOX enzyme complex, subunit B | soxB | | | | | **THIOM_000746** | | | | | |  | | | 186 | | | | |  | | |  | | | | | BGP_2304 BOGUAY_1092 **FLOR_02744**   *Ga0063879_02540* THII_1577 | | | | | thiosulfate oxidation | | |  |  |  |
| SOX enzyme complex,  subunit Y | soxY | | | | | **THIOM_003633** | | | | | |  | | | 98 | | | | |  | | |  | | | | | BEGALDRAFT_RS11400 BGP_4779 BOGUAY_0115 **FLOR_02368**   *Ga0063879_01141 Ga0063879_02538* THII_1579 | | | | | thiosulfate oxidation | | |  |  |  |
| SOX enzyme complex,  subunit AX | soxAX | | | | |  | | | | | |  | | |  | | | | |  | | |  | | | | | BEGALDRAFT_RS15060 BGP_5667 **FLOR_03004**   *Ga0063879_02537* THII_1580 | | | | | thiosulfate oxidation | | |  |  |  |
| SOX enzyme complex,  subunit Z | soxZ | | | | |  | | | | | |  | | |  | | | | |  | | |  | | | | | BEGALDRAFT_RS12570 BEGALDRAFT_RS11405 BEGALDRAFT_RS02680 BGP_4779 BGP_4778 BOGUAY_0116  **FLOR_02369**  *Ga0063879_02539 Ga0063879_01142* THII_1578 | | | | | thiosulfate oxidation | | |  |  |  |
| **Thiosulfate disproportionation** | | | | | | | | | | | | | | | | | | | | | | | | | | | | | | | | | | | |  |  |  |
| thiosulfate sulfurtransferase, rhodanese | tst | | | | | | | **THIOM_002093** | | | | 2.8.1.1 | | | 186 | | | | | + | | |  | | | | | BEGALDRAFT_RS08715 BGP_3637 BOGUAY_2819 BOGUAY_1655  *Ga0063879_03870 Ga0063879_06081* THII_1388 | | | | |  | | |  |  |  |
| **Sulfate uptake system and assimilatory sulfate reduction** | | | | | | | | | | | | | | | | | | | | | | | | | | | | | | | | | | | |  |  |  |
| sulfate permease, high affinity transporter | | SuIP | | | | | | **THIOM_002967 THIOM_003723** | | | |  | | | 80 146 | | | | | + | | |  | | | | | BOGUAY_3672 BGP_2594 BGP_3542 **FLOR_00582**  *Ga0063879_06509* THII_0448 | | | | |  | | |  |  |  |
| sulfate transporter, periplasmic binding protein | | sbp | | | | | |  | | | |  | | |  | | | | |  | | |  | | | | | BEGALDRAFT_RS16640  **FLOR_01671** | | | | |  | | |  |  |  |
| sulfate ABC transporter, ATPase subunit | | cysA | | | | | |  | | | |  | | |  | | | | |  | | |  | | | | | BEGALDRAFT_RS16655  **FLOR_00092** | | | | |  | | |  |  |  |
| sulfate ABC transporter, permease protein | | cysW | | | | | |  | | | |  | | |  | | | | |  | | |  | | | | | BEGALDRAFT_RS16650 | | | | |  | | |  |  |  |
| sulfate ABC transporter, permease protein | | cysT | | | | | |  | | | |  | | |  | | | | |  | | |  | | | | | BEGALDRAFT_RS16645 | | | | |  | | |  |  |  |
| adenylylsulfate kinase | | cysC | | | | | | **THIOM_001126** | | | | 2.7.1.25 | | | + | | | | |  | | |  | | | | | BEGALDRAFT_RS03835 BOGUAY_3365 **FLOR_01349**  *Ga0097846_100992*  THII_0538 | | | | |  | | |  |  |  |
| sulfate adenylyltransferase, large subunit | | cysN | | | | | |  | | | | 2.7.7.4 | | |  | | | | |  | | |  | | | | | BEGALDRAFT_RS05105 | | | | |  | | |  |  |  |
| sulfate adenylyltransferase, small subunit | | cysD | | | | | |  | | | | 2.7.7.4 | | |  | | | | |  | | |  | | | | | BEGALDRAFT_RS05100 | | | | |  | | |  |  |  |
| putative 3´phospho-adenylylsulfate reductase | | cysH | | | | | |  | | | | 1.8.4.8 | | |  | | | | |  | | |  | | | | | BEGALDRAFT_RS01495 **FLOR_03186** | | | | |  | | |  |  |  |
| sulfite reductase, NADPH flavoprotein subunit | | cysJ | | | | | |  | | | | 1.8.1.2 | | |  | | | | |  | | |  | | | | | BEGALDRAFT_RS06495 | | | | |  | | |  |  |  |
| sulfite reductase, NADPH hemeprotein subunit | | cysI | | | | | |  | | | | 1.8.1.2 | | |  | | | | |  | | |  | | | | | BEGALDRAFT_RS06500 | | | | |  | | |  |  |  |
| sulfite reductase, ferredoxin | | sir | | | | | |  | | | | 1.8.7.1 | | |  | | | | |  | | |  | | | | | **FLOR_03184** | | | | |  | | |  |  |  |
| **Heterodisulfide reductase** | | | | | | | | | | | | | | | | | | | | | | | | | | | | | | | | | | | | |  |  |
| heterodisulfide reductase, subunit A | | hdrA | | | | | | | **THIOM_005648 THIOM_000826** | | | | 1.8.98.1 | | | 135 131 | | | | + | | | |  | | | | | BOGUAY_3539 BOGUAY_3540 BOGUAY_2741 BGP_0252 BGP_0253 THII_RS11245  *Ga0063879_05765 Ga0063879_05624* THII_2277 THII_2278 | | | |  | | | |  |  |
| heterodisulfide reductase, subunit B | | hdrB | | | | | | |  | | | | 1.8.98.1 | | |  | | | |  | | | |  | | | | | BGP_2617  *Ga0063879_05764* THII_2280 THII_2508 | | | |  | | | |  |  |
| heterodisulfide reductase, subunit C | | hdrC | | | | | | | **THIOM_005425** | | | | 1.8.98.1 | | | 213 | | | | + | | | |  | | | | | BGP_2616  *Ga0063879_05763*  THII_2281 | | | |  | | | |  |  |
| heterodisulfide oxidoreductase, iron-sulfur cluster-binding subunit D | | hdrD | | | | | | | **THIOM_003319 THIOM_004757** | | | | 1.8.98.1 | | | 309 302 | | | |  | | | | + | | | | | BEGALDRAFT_RS05255 BOGUAY_3538 *Ga0063879_05622* | | | |  | | | |  |  |
| heterodisulfide oxidoreductase, iron-sulfur cluster-binding subunit E | | hdrE | | | | | | | **THIOM_003320** | | | | 1.8.98.1 | | | 368 | | | |  | | | | + | | | | | BOGUAY_3536 *Ga0063879_05621* | | | |  | | | |  |  |
| heterodisulfide reductase, cytochrome reductase subunit | | hdrF | | | | | | | **THIOM_003321** | | | | 1.8.98.1 | | | 220 | | | | + | | | |  | | | | | BOGUAY_3535 *Ga0063879_05620* | | | |  | | | |  |  |
| **Methyl viologen-reducing hydrogenase** | | | | | | | | | | | | | | | | | | | | | | | | | | | | | | | | | | | | |  |  |
| methyl viologen-reducing hydrogenase, delt subunit | | | mvhD | | | | | | **THIOM_005424** | | | | 1.12.99. | | | 112 | | | |  | | | | + | | | | | BOGUAY_3538  *Ga0063879_05623* | | | |  | | | |  |  |
| methyl viologen-reducing hydrogenase-associated ferredoxin | | | mvhF | | | | | | **THIOM_004285** | | | | 1.12.99. | | | 245 | | | | + | | | |  | | | | | *Ga0097846_120543** | | | | * Illumina metagenome | | | |  |  |
| nickel-dependent methyl viologen-reducing hydrogenase, large subunit | | | mvhA | | | | | | **THIOM_004286 THIOM_001444** | | | | 1.12.99. | | | 471 106 | | | |  | | | | + | | | | | BGP_0253  *Ga0097846_120544** | | | | * Illumina metagenome | | | |  |  |
| nickel-dependent methyl viologen-reducing hydrogenase, small subunit | | | mvhG | | | | | | **THIOM_004287** | | | | 1.12.99. | | | 313 | | | |  | | | | + | | | | | *Ga0097846_120545** | | | | functional similar to F420 non reducing- hydrogenase, * Illumina metagenome | | | |  |  |
| methyl viologen-reducing hydrogenase maturation protease | | | mvhP | | | | | | **THIOM_004289** | | | | 1.12.99. | | | 131 | | | |  | | | |  | | | | | *Ga0097846_120547** | | | | * Illumina metagenome | | | |  |  |
| **Na^+^-translocating membrane complex** | | | | | | | | | | | | | | | | | | | | | | | | | | | | | | | | | | | | |  |  |
| electron transport complex, subunit A | | | rnfA | | | | | | **THIOM_000874** | | | |  | | | 191 | | | |  | | | | + | | | | | BEGALDRAFT_RS01690 BEGALDRAFT_RS09750 BOGUAY_2644 BOGUAY_0446 BGP_4894 BGP_4895 **FLOR_00005 FLOR_00042**  *Ga0063879_03590*   *Ga0063879_04683*  THII_2165 THII_2570 | | | |  | | | |  |  |
| electron transport complex, subunit B, ferredoxin subunit | | | rnfB | | | | | | **THIOM_001510 THIOM_000450** | | | |  | | | 298 70 | | | |  | | | | + | | | | | BEGALDRAFT_RS01685 BEGALDRAFT_RS09755 BOGUAY_1961 BOGUAY_0445 BGP_5165  **FLOR_00004 FLOR_01801**   *Ga0063879_03589*  *Ga0063879_05886* THII_2164 THII_2569 | | | |  | | | |  |  |
| electron transport complex, subunit C, flavin subunit | | | rnfC | | | | | | **THIOM_004702** | | | |  | | | 484 | | | | + | | | | + | | | | | BEGALDRAFT_RS01680 BEGALDRAFT_RS06705 BEGALDRAFT_RS09760 BOGUAY_0327 BOGUAY_0444 BGP_1887  **FLOR_00003 FLOR_03168** *Ga0097846_112055**  *Ga0063879_03588*  THII_2163 THII_2568 | | | | * Illumina metagenome | | | |  |  |
| electron transport complex, subunit D | | | rnfD | | | | | | **THIOM_000524 THIOM_000098 THIOM_005455** | | | |  | | | 300 239 344 | | | | + | | | |  | | | | | BEGALDRAFT_RS01675 BEGALDRAFT_RS09765 BOGUAY_4422 BOGUAY_2718 BGP_2194 BGP_2824 **FLOR_00109 FLOR_02202** *Ga0063879_01995 Ga0063879_04152* THII_2162 THII_2567 | | | |  | | | |  |  |
| electron transport complex, subunit G | | | rnfG | | | | | | **THIOM_002541** | | | |  | | | 207 | | | |  | | | | + | | | | | BEGALDRAFT_RS01670 BEGALDRAFT_RS09770 BOGUAY_1747 BOGUAY_2719 BGP_5931 BGP_6563  **FLOR_00108 FLOR_02203** *Ga0063879_04153* THII_2161 THII_2566 | | | |  | | | |  |  |
| electron transport complex, subunit E | | | rnfE | | | | | | **THIOM_003670** | | | |  | | | 168 | | | | + | | | |  | | | | | BEGALDRAFT_RS01665 BEGALDRAFT_RS08995 BOGUAY_2233 BOGUAY_2643 **FLOR_00471 FLOR_02204** *Ga0063879_02581 Ga0063879_04682* THII_2523 THII_2565 | | | |  | | | |  |  |
| hypothetical protein | | | rnfH | | | | | |  | | | |  | | |  | | | |  | | | |  | | | | | BEGALDRAFT_RS01660 BOGUAY_1193 | | | |  | | | |  |  |
| **Uptake hydrogenase** | | | | | | | | | | | | | | | | | | | | | | | | | | | | | | | | | | | | |  |  |
| respiratory membrane-bound hydrogen uptake [Ni,Fe] hydrogenase, small subunit | | | hupS | | | | | | **THIOM_001669** | | | | 1.12.99.6 | | | 57 | | | | + | | | |  | | | | | BEGALDRAFT_RS02110 BOGUAY_0684  **FLOR_00588 FLOR_02642**  *Ga0063879_05380* THII_2459 THII_2462 | | | |  | | | |  |  |
| respiratory membrane-bound hydrogen uptake [Ni,Fe] hydrogenase, large subunit | | | hupL | | | | | | **THIOM_000798** | | | | 1.12.99.6 | | | 256 | | | | + | | | | + | | | | | BEGALDRAFT_RS02115 FLOR_00363 **FLOR_02641**   *Ga0063879_05378*  THII_3315 | | | |  | | | |  |  |
| [Ni,Fe] hydrogenase 1b-type cytochrome | | | hupC | | | | | |  | | | | 1.12.99.6 | | |  | | | |  | | | |  | | | | | BEGALDRAFT_RS02120 BOGUAY_1888 BOGUAY_2385 BOGUAY_2760 **FLOR_02639** | | | |  | | | |  |  |
| [Ni,Fe] hydrogenase maturation protein | | | hupD | | | | | |  | | | | 3.4.24.- | | |  | | | |  | | | |  | | | | | **FLOR_03012** | | | |  | | | |  |  |
| [Ni,Fe] hydrogenase expression protein | | | hupH | | | | | |  | | | |  | | |  | | | |  | | | |  | | | | | BEGALDRAFT_RS07090 **FLOR_00978** | | | |  | | | |  |  |
| **Other hydrogenases and maturation proteins** | | | | | | | | | | | | | | | | | | | | | | | | | | | | | | | | | | | | |  |  |
| [Ni,Fe] hydrogenase, large subunit | | | hynL | | | | | | **THIOM_004035** | | | |  | | | 592 | | | |  | | | |  | | | | | BEGALDRAFT_RS05265 BOGUAY_4411 **FLOR_00363**  *Ga0063879_01537* | | | |  | | | |  |  |
| [Ni,Fe] hydrogenase, small subunit | | | hynS | | | | | |  | | | |  | | |  | | | |  | | | |  | | | | | BEGALDRAFT_RS05250 BOGUAY_1709  **FLOR_00588**  *Ga0063879_01538 Ga0063879_01536* | | | |  | | | |  |  |
| [Ni,Fe] hydrogenase, small, subunit | | | hycG | | | | | |  | | | |  | | |  | | | |  | | | |  | | | | | BEGALDRAFT_13385 THII0841 | | | |  | | | |  |  |
| [Ni,Fe] hydrogenase, small, subunit | | | hycE | | | | | |  | | | |  | | |  | | | |  | | | |  | | | | | BEGALDRAFT_2426 THII_0915 | | | |  | | | |  |  |
| [Ni,Fe] hydrogenase, large subunit | | | hyhA | | | | | | **THIOM_000518** | | | | 1.12.1.2 | | | 247 | | | | + | | | |  | | | | | BEGALDRAFT_RS15815  **FLOR_00048** THII_2459 THII_2462 | | | |  | | | |  |  |
| [Ni,Fe] hydrogenase, small subunit | | | hyhB | | | | | |  | | | |  | | |  | | | |  | | | |  | | | | | BEGALDRAFT_RS15805  *Ga0063879_07217 Ga0063879_05378* | | | |  | | | |  |  |
| [Ni,Fe] hydrogenase, maturation protein | | | hyhC | | | | | |  | | | |  | | |  | | | |  | | | |  | | | | | BEGALDRAFT_RS15810 | | | |  | | | |  |  |
| [Ni,Fe] hydrogenase, delta subunit | | | hyhD | | | | | |  | | | |  | | |  | | | |  | | | |  | | | | | BEGALDRAFT_RS15820 **FLOR_00047** | | | |  | | | |  |  |
| [Ni,Fe] hydrogenase, gamma subunit | | | hyhG | | | | | |  | | | |  | | |  | | | |  | | | |  | | | | | BEGALDRAFT_RS15800 | | | |  | | | |  |  |
| [Ni, Fe] hydrogenase, alpha subunit, F420-reducing | | | frhA | | | | | |  | | | | 1.12.98.1 | | |  | | | |  | | | |  | | | | |  | | | |  | | | |  |  |
| [Ni, Fe] hydrogenase, beta subunit, F420-reducing | | | frhB | | | | | | **THIOM_000269** | | | | 1.12.98.1 | | | 192 | | | |  | | | |  | | | | |  | | | |  | | | |  |  |
| [Ni, Fe] hydrogenase, beta subunit, F420-reducing | | | frhG | | | | | |  | | | | 1.12.98.1 | | |  | | | |  | | | |  | | | | | THII_1522 | | | |  | | | |  |  |
| [Ni, Fe] hydrogenase, large subunit | | | hyaB | | | | | | **THIOM_004755** | | | | 1.12.5.1 | | | 219 | | | |  | | | |  | | | | |  | | | |  | | | |  |  |
| [Ni, Fe] hydrogenase, small subunit | | | hyaA | | | | | | **THIOM_005381** | | | | 1.12.5.1 | | | 153 | | | |  | | | |  | | | | |  | | | |  | | | |  |  |
| [Ni, Fe] cytochrome c3 hydrogenase, small subunit | | | hydA | | | | | |  | | | | 1.12.2.1 | | |  | | | |  | | | |  | | | | |  | | | |  | | | |  |  |
| [Ni, Fe] cytochrome c3 hydrogenase, large subunit | | | hydB | | | | | | **THIOM_002731** | | | | 1.12.2.1 | | | 222 | | | | + | | | |  | | | | |  | | | |  | | | |  |  |
| [Ni, Fe] cytochrome c3 hydrogenase, anchor | | | hydC | | | | | |  | | | | 1.12.2.1 | | |  | | | |  | | | |  | | | | |  | | | |  | | | |  |  |
| [Ni, Fe] cytochrome c3 hydrogenase, protease | | | hydD | | | | | |  | | | |  | | |  | | | |  | | | |  | | | | |  | | | |  | | | |  |  |
| [Ni, Fe] cytochrome c3 hydrogenase, maturation | | | hydE | | | | | |  | | | |  | | |  | | | |  | | | |  | | | | |  | | | |  | | | |  |  |
| **hydrogenase maturation proteins** | | | | | | | | | | | | | | | | | | | | | | | | | | | | | | | | | | | | |  |  |
| hydrogenase maturation protein, Ni insertion protein | | | hypA/  hybF | | | | | |  | | | |  | | |  | | | |  | | | |  | | | | | BOGUAY_1790 FLOR_00762 THII_3544 | | | |  | | | |  |  |
| hydrogenase maturation protein, GTPase, Ni insertion | | | hypB | | | | | |  | | | |  | | |  | | | |  | | | |  | | | | | BOGUAY_1512 FLOR_00958 FLOR_03103 THII_2973 | | | |  | | | |  |  |
| hydrogenase maturation protein, chaperone, Fe insertion | | | hypC/  hypG | | | | | |  | | | |  | | |  | | | |  | | | |  | | | | | BOGUAY_1791 FLOR_00763 THII_3543 | | | |  | | | |  |  |
| hydrogenase maturation protein, Fe-S protein, Fe insertion | | | hypD | | | | | |  | | | |  | | |  | | | |  | | | |  | | | | | BOGUAY_3052 BOGUAY_1513 FLOR_03072 THII_3592 THII_3675 | | | |  | | | |  |  |
| hydrogenase maturation protein, CO/CN synthesis, ATPase | | | hypE | | | | | |  | | | |  | | |  | | | |  | | | |  | | | | | BOGUAY_3356 FLOR_01477 FLOR_03103 THII_2694 | | | |  | | | |  |  |
| hydrogenase maturation protein, CO/CN synthesis, CP phosphatase | | | hypF | | | | | | **THIOM_004915** | | | |  | | | 74 | | | |  | | | |  | | | | | FLOR_02743 THII_3213 | | | |  | | | |  |  |
| Endopeptidase | | | hyaD, hybD, hycI | | | | | |  | | | |  | | |  | | | |  | | | |  | | | | |  | | | |  | | | |  |  |
| CP synthetase | | | carA/carB | | | | | |  | | | |  | | |  | | | |  | | | |  | | | | |  | | | |  | | | |  |  |
| **complex I** | | | | | | | | | | | | | | | | | | | | | | | | | | | | | | | | | | | | | |  |
| NADH dehydrogenase, subunit A | | nouA | | | |  | | | | 1.6.5.3 | | | | | | |  | | | |  | | | |  | | | | | BEGALDRAFT_RS09260 BGP_0197 BOGUAY_1243 **FLOR_00620**  *Ga0063879_03331* THII_RS02595 | | | |  | | | |  |
| NADH dehydrogenase, subunit B | | nouB | | | |  | | | | 1.6.5.3 | | | | | | |  | | | |  | | | |  | | | | | BEGALDRAFT_RS09265 BGP_0198 BGP_0509 BOGUAY_1242 BOGUAY_3117  **FLOR_00621** *Ga0063879_03330*  THII_RS02600 | | | |  | | | |  |
| NADH dehydrogenase, subunit C | | nouC | | | |  | | | | 1.6.5.3 | | | | | | |  | | | |  | | | |  | | | | | BEGALDRAFT_RS09270 BGP_0508 BOGUAY_1236  **FLOR_00622**  *Ga0063879_03329*  THII_RS02605 | | | |  | | | |  |
| NADH dehydrogenase, subunit D, iron-sulfur protein | | nouD | | | | **THIOM_005614** | | | | 1.6.5.3 | | | | | | | 232 | | | | + | | | |  | | | | | BEGALDRAFT_RS09275 BGP_0004 BGP_4191 BOGUAY_1234  **FLOR_01604 FLOR_02881**   *Ga0063879_03328* THII_RS02610 | | | |  | | | |  |
| NADH dehydrogenase, subunit E, FMN | | nouE | | | | **THIOM_005300** | | | | 1.6.5.3 | | | | | | | 165 | | | |  | | | | + | | | | | BEGALDRAFT_RS09280 BGP_0171 BOGUAY_1225 *Ga0063879_03326*  THII_RS02625 | | | |  | | | |  |
| NADH dehydrogenase, subunit F, FMN | | nouF | | | | **THIOM_001428 THIOM_005301** | | | | 1.6.5.3 | | | | | | | 276 121 117 | | | | + | | | |  | | | | | BEGALDRAFT_RS09285 BGP_5079 BGP_0263 BOGUAY_1100 BOGUAY_2594 **FLOR_01601 FLOR_01761**   **FLOR_00941**  *Ga0063879_03323 Ga0063879_05610* THII_RS02645 | | | |  | | | |  |
| NADH dehydrogenase, subunit G, FMN | | nouG | | | | **THIOM_001903 THIOM_004085** | | | | 1.6.5.3 | | | | | | | 309 395 | | | | + | | | |  | | | | | BEGALDRAFT_RS09290 BGP_2702 BOGUAY_3220 *Ga0063879_03320*  THII_RS14615 | | | |  | | | |  |
| NADH dehydrogenase, subunit H, membrane complex | | nouH | | | | **THIOM_000300 THIOM_000970** | | | | 1.6.5.3 | | | | | | | 304 333 | | | |  | | | | + | | | | | BEGALDRAFT_RS09295 BGP_1810 BGP_1811 BGP_1812 BGP_5679 BOGUAY_3219 BOGUAY_1731  **FLOR_02882**  *Ga0063879_03319 Ga0063879_03867*  THII_RS14610 | | | |  | | | |  |
| NADH dehydrogenase, subunit H, iron-sulfur protein | | nouI | | | | **THIOM_000969** | | | | 1.6.5.3 | | | | | | | 80 | | | | + | | | |  | | | | | BEGALDRAFT_RS09300 BGP_1809 BOGUAY_3218  **FLOR_02883** *Ga0063879_03318*  THII_RS14605 | | | |  | | | |  |
| NADH dehydrogenase, subunit J, membrane complex | | nouJ | | | | **THIOM_000720** | | | | 1.6.5.3 | | | | | | | 119 | | | | + | | | |  | | | | | BEGALDRAFT_RS09310 BGP_0811 BOGUAY_3216  **FLOR_02885** *Ga0063879_03316*  THII_RS14590 | | | |  | | | |  |
| NADH dehydrogenase, subunit K, membrane complex | | nouK | | | | **THIOM_002334 THIOM_000721** | | | | 1.6.5.3 | | | | | | | 117 144 | | | | + | | | |  | | | | | BEGALDRAFT_RS09315 BGP_0509 BGP_0810 BOGUAY_3215  **FLOR_02886**  *Ga0063879_03315*  THII_RS14585 | | | |  | | | |  |
| NADH dehydrogenase, subunit L, membrane complex | | nouL | | | | **THIOM_001827 THIOM_003965** | | | | 1.6.5.3 | | | | | | | 223 376 | | | | + | | | |  | | | | | BEGALDRAFT_RS09320 BGP_5518 BGP_5519 BOGUAY_4484 **FLOR_02887**  *Ga0063879_03314* THII_RS14580 | | | |  | | | |  |
| NADH dehydrogenase, subunit M, membrane complex | | nouM | | | | **THIOM_003964** | | | | 1.6.5.3 | | | | | | | 505 | | | | + | | | |  | | | | | BEGALDRAFT_RS09325 BGP_4202 BOGUAY_1496 BOGUAY_2740  **FLOR_02888** *Ga0063879_02302 Ga0063879_03313*  THII_RS14575 | | | |  | | | |  |
| NADH dehydrogenase, subunit N, membrane complex | | nouN | | | | **THIOM_003471 THIOM_004849** | | | | 1.6.5.3 | | | | | | | 50 328 | | | | + | | | |  | | | | | BEGALDRAFT_RS09330 BGP_3173 BGP_3174 BGP_5502 BGP_6044 BOGUAY_3866 BOGUAY_2982 BOGUAY_0688 FLOR_02893 *Ga0063879_05163* THII_RS14570 | | | |  | | | |  |
| NAD-dependent epimerase/ dehydratase | |  | | | | **THIOM_000403 THIOM_000510 THIOM_002209 THIOM_003008 THIOM_004190 THIOM_004559 THIOM_005608 THIOM_005687 THIOM_005688** | | | | 1.6.5.3 | | | | | | | 323 239 310 99 96 157 329 211 287 | | | | + | | | |  | | | | | BEGALDRAFT_RS00635 BGP_0215 BGP_1167 BGP_1168 BGP_1982 BGP_3469 BOGUAY_2206 BOGUAY_4092 **FLOR_01345 FLOR_03013**  *Ga0063879_00671*  *Ga0063879_01054*  THII_RS18155 | | | |  | | | |  |
| ubiquinol-cytochrome c reductase, iron-sulfur subunit | | petA | | | | **THIOM_005159** | | | | 1.10.2.2 | | | | | | | 121 | | | |  | | | |  | | | | | BEGALDRAFT_RS00135 BGP_0838 BOGUAY_0396  **FLOR_00183**   *Ga0063879_02033*  THII_RS04230 | | | |  | | | |  |
| ubiquinol-cytochrome c reductase, cytochrome b subunit | | petB | | | | **THIOM_005158** | | | | 1.10.2.2 | | | | | | | 408 | | | |  | | | | + | | | | | BEGALDRAFT_RS00140 BGP_0839 BGP_5122 BOGUAY_0395 **FLOR_00184** *Ga0063879_02032*  THII_RS04235 | | | |  | | | |  |
| ubiquinol-cytochrome c reductase, cytochrome c1 subunit | | petC | | | | **THIOM_004397 THIOM_005157** | | | | 1.10.2.2 | | | | | | | 100 142 | | | | + | | | |  | | | | | BEGALDRAFT_RS00145 BGP_6663 BOGUAY_0394  **FLOR_00185**   *Ga0063879_02031*  THII_RS04240 | | | |  | | | |  |
| cyotchrome c oxidase cbb3-type, subunit I | | CcoN | | | | **THIOM_002847 THIOM_005732** | | | | 1.9.3.1 | | | | | | | 132 113 | | | | + | | | |  | | | | | BEGALDRAFT_RS01065 BGP_2112 BOGUAY_3546  **FLOR_00924**  *Ga0063879_03369*  THII_RS01720 | | | |  | | | |  |
| cyotchrome c oxidase cbb3-type, subunit II | | CcoO | | | | **THIOM_002848** | | | | 1.9.3.1 | | | | | | | 205 | | | |  | | | | + | | | | | BEGALDRAFT_RS01070 BGP_3209 BOGUAY_3547 **FLOR_00573 FLOR_00923**  *Ga0063879_03370* THII_RS01715 | | | |  | | | |  |
| cyotchrome c oxidase cbb3-type, subunit IV | | CcoQ | | | | **THIOM_002849** | | | | 1.9.3.1 | | | | | | | 59 | | | |  | | | | + | | | | | BEGALDRAFT_RS01080 BGP_3208 BOGUAY_3548 **FLOR_00572**   *Ga0063879_03372* THII_RS01710 | | | |  | | | |  |
| cyotchrome c oxidase cbb3-type, subunit III | | CcoP | | | | **THIOM_002850** | | | | 1.9.3.1 | | | | | | | 301 | | | |  | | | | + | | | | | BEGALDRAFT_RS01075 BGP_3207 BOGUAY_3549  **FLOR_00571**   *Ga0063879_03371*  THII_RS01705 | | | |  | | | |  |
| cyotchrome c oxidase accessory protein | | CcoG | | | | **THIOM_002851** | | | | 1.9.3.1 | | | | | | | 229 | | | | + | | | |  | | | | |  | | | |  | | | |  |
| cytochrome d ubiquinol oxidase, subunit I | | cydA | | | | **THIOM_002362** | | | | 1.10.3.- | | | | | | | 190 | | | |  | | | |  | | | | | BEGALDRAFT_RS16775 BOGUAY_1879 BOGUAY_0143 *Ga0063879_02070* | | | |  | | | |  |
| cytochrome d ubiquinol oxidase, subunit II | | cydB | | | |  | | | | 1.10.3.- | | | | | | |  | | | |  | | | |  | | | | | BEGALDRAFT_RS16770 BOGUAY_1880  *Ga0063879_02069* | | | |  | | | |  |
| protoheme IX farnesyltransferase | | cyoE | | | | **THIOM_004359** | | | | 2.5.1. | | | | | | | 324 | | | | + | | | |  | | | | | BGP_2365 *Ga0097846_120564** | | | | * Illumina metagenome | | | |  |
| cytochrome c oxidase, aa3 type, subunit III | | coxC | | | | **THIOM_001795** | | | | 1.9.3.1 | | | | | | | 92 | | | | + | | | |  | | | | | BGP_2863 | | | |  | | | |  |
| cytochrome c oxidase, aa3 type, subunit II | | coxB | | | | **THIOM_001934** | | | | 1.9.3.1 | | | | | | | 260 | | | | + | | | |  | | | | | BGP_2865 | | | |  | | | |  |
| cytochrome c oxidase, aa3 type, subunit I | | coxA | | | | **THIOM_004508** | | | | 1.9.3.1 | | | | | | | 263 | | | | + | | | |  | | | | | BGP_2866 | | | |  | | | |  |
| cytochrome c oxidase synthesis factor | | SCO | | | |  | | | |  | | | | | | |  | | | |  | | | |  | | | | | BGP_2864 | | | |  | | | |  |
| V-type ATP (sodium) synthase subunit A | | atpA/ ntpA | | | | **THIOM_004022** | | | | 3.6.3.14/ 3.6.1.15 | | | | | | | 593 | | | |  | | | | + | | | | | BGP_4884 BOGUAY_2821  **FLOR_01562** *Ga0063879_02076*  THII_RS05980 | | | |  | | | |  |
| V-type ATP (sodium) synthase subunit B | | atpB/ ntpB | | | |  | | | | 3.6.3.14/ 3.6.1.15 | | | | | | |  | | | |  | | | |  | | | | | BGP_3881 BOGUAY_2662  **FLOR_01509** *Ga0097846_111343**  THII_RS02335 | | | | * Illumina metagenome | | | |  |
| V-type ATP (sodium) synthase subunit C | | atpC/ ntpC | | | | **THIOM_004016** | | | | 3.6.3.14/ 3.6.1.15 | | | | | | | 328 | | | | + | | | |  | | | | | BGP_0940 BOGUAY_2136 BOGUAY_2457  **FLOR_00138**  *Ga0063879_03207* | | | |  | | | |  |
| V-type (sodium) ATP synthase subunit D | | atpD/ ntpD | | | | **THIOM_000063** | | | | 3.6.3.14/ 3.6.1.15 | | | | | | | 210 | | | |  | | | | + | | | | | BGP_3601 BOGUAY_1958 BOGUAY_1959 BOGUAY_2137 **FLOR_02619** *Ga0063879_02106* | | | |  | | | |  |
| V-type (sodium) ATP synthase subunit E | | atpE/ ntpE | | | | **THIOM_004023** | | | | 3.6.3.14/ 3.6.1.15 | | | | | | | 140 | | | | + | | | |  | | | | | BOGUAY_2822 **FLOR_01561** *Ga0063879_02077* THII_RS05985 | | | |  | | | |  |
| V-type (sodium) ATP synthase subunit E | | atpF/ ntpF | | | | **THIOM_000861** | | | | 3.6.3.14/ 3.6.1.15 | | | | | | | 106 | | | | + | | | |  | | | | | *Ga0063879_00610*  THII_RS05990 | | | |  | | | |  |
| V-type (sodium) ATP synthase subunit H | | atpH/ ntpH | | | | **THIOM_004017** | | | | 3.6.3.14/ 3.6.1.15 | | | | | | | 124 | | | | + | | | |  | | | | |  | | | |  | | | |  |
| V-type (sodium) ATP synthase subunit I | | atpI/ ntpI | | | |  | | | | 3.6.3.14/ 3.6.1.15 | | | | | | |  | | | |  | | | |  | | | | | BGP_1588 BOGUAY_0078 BOGUAY_3163 **FLOR_00139**  *Ga0063879_02400 Ga0063879_04887*  THII_RS05600 | | | |  | | | |  |
| V-type (sodium) ATP synthase subunit K | | atpK/ ntpK | | | | **THIOM_000862** | | | | 3.6.3.14/ 3.6.1.15 | | | | | | | 92 | | | | + | | | |  | | | | | BGP_2819 BOGUAY_2663 **FLOR_00638** *Ga0063879_00611* THII_RS05995 | | | |  | | | |  |
| F-type ATP synthase, subunit epsilon | | atpC | | | | **THIOM_003949** | | | | 3.6.3.14 | | | | | | | 138 | | | | + | | | |  | | | | | BEGALDRAFT_RS15480 BGP_2403  **FLOR_00765 FLOR_00766**  *Ga0063879_02412* THII_RS14500 | | | |  | | | |  |
| F-type ATP synthase, subunit beta | | atpD | | | |  | | | |  | | | | | | |  | | | |  | | | |  | | | | | BEGALDRAFT_RS15485 BGP_2491  **FLOR_00767**   *Ga0063879_02411*  THII_RS14505 | | | |  | | | |  |
| F-type ATP synthase, subunit gamma | | atpG | | | | **THIOM_002006** | | | | 3.6.3.14 | | | | | | | 287 | | | |  | | | | + | | | | | BEGALDRAFT_RS15490 BGP_2492 BGP_1963 BOGUAY_1279  **FLOR_00768**  *Ga0063879_02409*  THII_RS14510 | | | |  | | | |  |
| F-type ATP synthase, subunit alpha | | atpA | | | |  | | | | 3.6.3.14 | | | | | | |  | | | |  | | | |  | | | | | BEGALDRAFT_RS15495 BGP_0784 BOGUAY_3168  **FLOR_01115**   *Ga0063879_02405* THII_RS14525 | | | |  | | | |  |
| F-type ATP synthase, subunit delta | | atpH | | | |  | | | | 3.6.3.14 | | | | | | |  | | | |  | | | |  | | | | | BEGALDRAFT_RS15500 BGP_0785 BOGUAY_3167 **FLOR_01114** *Ga0063879_02404* THII_RS14530 | | | |  | | | |  |
| F-type ATP synthase, subunit B | | atpF | | | |  | | | | 3.6.3.14 | | | | | | |  | | | |  | | | |  | | | | | BEGALDRAFT_RS15505 BGP_0786 BOGUAY_3166 **FLOR_01113**  *Ga0063879_02403* THII_RS14535 | | | |  | | | |  |
| F-type ATP synthase, chain C | | atpE | | | |  | | | | 3.6.3.14 | | | | | | |  | | | |  | | | |  | | | | | BEGALDRAFT_RS15510 BGP_0787 BOGUAY_3165 FLOR_01112  *Ga0063879_02402*  THII_RS14540 | | | |  | | | |  |
| F-type ATP synthase,chain A | | atpB | | | |  | | | | 3.6.3.14 | | | | | | |  | | | |  | | | |  | | | | | BEGALDRAFT_RS15515 BGP_0788 BGP_0789 BOGUAY_3164 **FLOR_01111**  **FLOR_02054** *Ga0063879_02401* THII_RS14545 | | | |  | | | |  |
| F-type ATP synthase protein I | | atpI/ ntpI | | | |  | | | | 3.6.3.14 | | | | | | |  | | | |  | | | |  | | | | | BEGALDRAFT_RS15520 BGP_0790 BOGUAY_3163 *Ga0063879_02400* | | | |  | | | |  |
| polyphosphate kinase | | ppk1 | | | | **THIOM_004356** | | | | 2.7.4.1 | | | | | | | 654 | | | |  | | | |  | | | | | BEGALDRAFT_RS00195 BGP_5434 BOGUAY_0085 **FLOR_02687**   *Ga0097846_120561** THII_RS14675 | | | | * Illumina metagenome | | | |  |
| membrane-bound proton translocating pyrophosphatase/ inorganic pyrophosphatase | | hppA/ ppa/ LHPP | | | |  | | | | 3.6.1.1 | | | | | | |  | | | |  | | | |  | | | | | BEGALDRAFT_RS00795 (hppA) BEGALDRAFT_RS03865 (ppA) BEGALDRAFT_RS08215 (LHPP) BGP_2409 (hppA) BOGUAY_4300 (hppA) **FLOR_00254 (ppa) FLOR_00748 (hppA) FLOR_01358 (hppA) FLOR_02192 (hppA)**   *Ga0063879_00607*  THII_RS03745 (hppA) | | | |  | | | |  |
| cytochrome c5 | |  | | | | **THIOM_001753 THIOM_000664** | | | |  | | | | | | | 61 401 | | | |  | | | |  | | | | | BEGALDRAFT_RS07495 BOGUAY_3646 BOGUAY_3647  *Ga0063879_01043*  THII_RS20085 | | | |  | | | |  |
| phosphate ABC transporter, inner membrane subunit | | pstA | | | | | **THIOM_000071** | | | 3.6.3.27 | | | | | | | 236 | | | | + | | | |  | | | | | BEGALDRAFT_RS04320 BA16_208 BGP_3522 BOGUAY_3725 **FLOR_00686** *Ga0063879_00994* Ga0063879_00995** THII_RS16480 | | | | * gene disrution by stop codon | | | | |
| phosphate ABC transporter, ATP binding subunit | | pstB | | | | | **THIOM_004380** | | | 3.6.3.27 | | | | | | | 285 | | | |  | | | | + | | | | | BEGALDRAFT_RS-04325 BGP_2125 BOGUAY_3724 **FLOR_00687** *Ga0063879_06301* Ga0063879_06302* Ga0097846_124704*** THII_RS16475 | | | | * gene disrution by stop codon and repetitive elment (MITE) at tail of 06302, ** Illumina metagenome | | | | |
| phosphate ABC transporter, inner membrane component | | pstC | | | | | **THIOM_002928** | | | 3.6.3.27 | | | | | | | 506 | | | | + | | | |  | | | | | BEGALDRAFT_RS04315 BGP_3523 BOGUAY_3726 **FLOR_00685** THII_RS16485 | | | |  | | | | |
| phosphate ABC transporter, periplasmatic phosphate-binding protein | | pstS | | | | |  | | | 3.6.3.27 | | | | | | |  | | | |  | | | |  | | | | | BEGALDRAFT_RS06280 BGP_3525 BOGUAY_ 3729 BOGUAY_ 3728 **FLOR_02873 FLOR_01348** *Ga0063879_03844* THII_RS08835 | | | |  | | | | |
| phosphate ABC transporter, phosphat binding protein | | pstD | | | | |  | | | 3.6.3.27 | | | | | | |  | | | |  | | | |  | | | | | BEGALDRAFT_RS10110 | | | |  | | | | |
| phosphate regulon sensor protein | | phoR | | | | | **THIOM_005784** | | |  | | | | | | | 421 | | | | + | | | |  | | | | | BEGALDRAFT_RS03400 BOGUAY_4336 *Ga0063879_02710* Ga0063879_02711* Ga0063879_02712** | | | | * gene disrution by stop codon | | | | |
| phsophate transport system regulator protein | | phoU | | | | | **THIOM_004381** | | |  | | | | | | | 99 | | | | + | | | |  | | | | | BEGALDRAFT_RS04330 BGP_1356 BOGUAY_3723 **FLOR_02932 FLOR_00688** *Ga0063879_06302** | | | | * below cutoff | | | | |
| phosphate stravation-inducible protein | | phoH | | | | |  | | |  | | | | | | |  | | | |  | | | |  | | | | | BEGALDRAFT_0363 BOGUAY_2188 **FLOR_03136** *Ga0063879_03420* | | | |  | | | | |
| phosphate regulon transcriptional regulatory | | phoB | | | | |  | | |  | | | | | | |  | | | |  | | | |  | | | | | BEGALDRAFT_2315 BOGUAY_4337 | | | |  | | | | |
| phosphate-selective porin O and P | | phoE | | | | | **THIOM_000583 THIOM_005092** | | |  | | | | | | | 387 391 | | | |  | | | | + | | | | | BGP_4216 BGP_0268 BGP_0269 BOGUAY_2057 | | | |  | | | | |
| polyphosphate:AMP phosphotransferase | | pap | | | | | **THIOM_001741** | | | 2.7.4.- | | | | | | |  | | | |  | | | |  | | | | | BEGALDRAFT_RS09950 BOGUAY_0604 **FLOR_02303** *Ga0063879_00673* | | | |  | | | | |
| acetyl-CoA acetyltransferase | | phaA/ phbA | | | | | **THIOM_002381** | | | 2.3.1.9 | | | | | | | 370 | | | |  | | | | + | | | | | BEGALDRAFT_RS04815 BEGALDRAFT_RS14770 **FLOR_00713** *Ga0097846_122421** THII_RS04170 | | | | * Illumina metagenome | | | | |
| acetoacetyl-CoA reductase | | phaB/ phbB | | | | |  | | | 1.1.1.36 | | | | | | |  | | | |  | | | |  | | | | | BEGALDRAFT_RS14760 BGP_0317 BOGUAY_0874 **FLOR_00822** THII_RS04160 | | | |  | | | | |
| poly-beta hydroxybutyrate polymerase/ poly(R)-hydroxyalkanoate acid synthase, class I | | phaC | | | | |  | | | 2.3.1- | | | | | | |  | | | |  | | | |  | | | | | BEGALDRAFT_RS05880 BEGALDRAFT_RS02230 BEGALDRAFT_RS03900 BEGALDRAFT_RS17460 **FLOR_01645 FLOR_03182 FLOR_03197** THII_RS06035 | | | |  | | | | |
| poly(R)-hydroxyalaknoic acid synthase, class III | | phaE | | | | |  | | |  | | | | | | |  | | | |  | | | |  | | | | | BEGALDRAFT_RS18140 **FLOR_01646** THII_RS06370 | | | |  | | | | |
| poly(3-hydroxybutyrate) depolymerase | | phaZ | | | | |  | | | 3.1.1.75 | | | | | | |  | | | |  | | | |  | | | | | BEGALDRAFT_RS09460 BEGALDRAFT_RS09465 **FLOR_03285** | | | |  | | | | |
| putative regulator of polymer accumulation | | phaR | | | | |  | | |  | | | | | | |  | | | |  | | | |  | | | | | BEGALDRAFT_RS14765 BGP_4849 **FLOR_00714** | | | |  | | | | |

locus tags in italic of the *Ca.* T nelsonii Bud S10 genome are IMG/ER database entries

locus tags in bold of the *Beggiatoa* sp. 35Flor genome are internal JCoast entries, the genome will be published elsewhere

all other locus tags are NCBI entries

# Supplementary Figures

**Supplementary Figure S1**: Genomic organization of heterodisulfide reductases (*hdr*) operon.

**Supplementary Figure S2**: Genomic organization of the methyl viologen-reducing hydrogenase (*mvh*) operon.

**
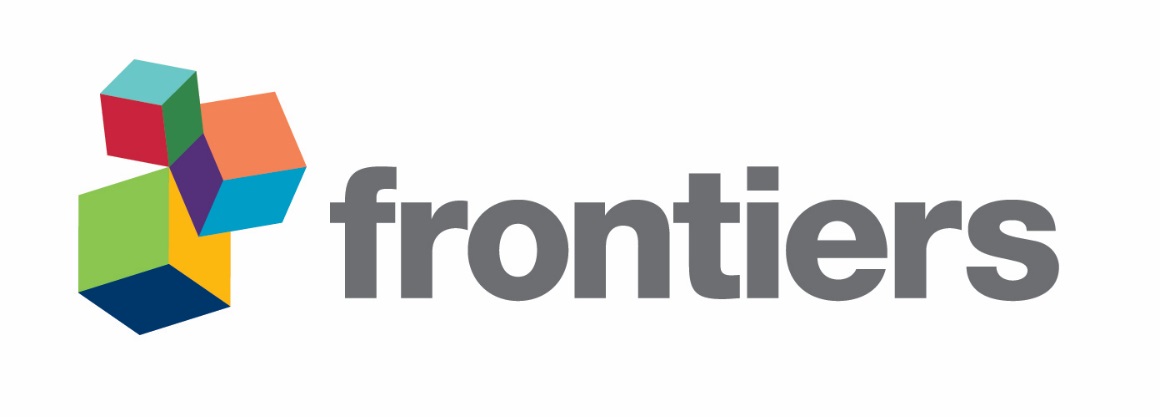
**
