# Supplementary figures and images for: Single-cell Sequencing of Thiomargarita Reveals Genomic Flexibility for Adaptation to Dynamic Redox Conditions
Source: Front Microbiol. 2016 Jun 21;7:964. doi: 10.3389/fmicb.2016.00964 (PMC4914600; doi:10.3389/fmicb.2016.00964)

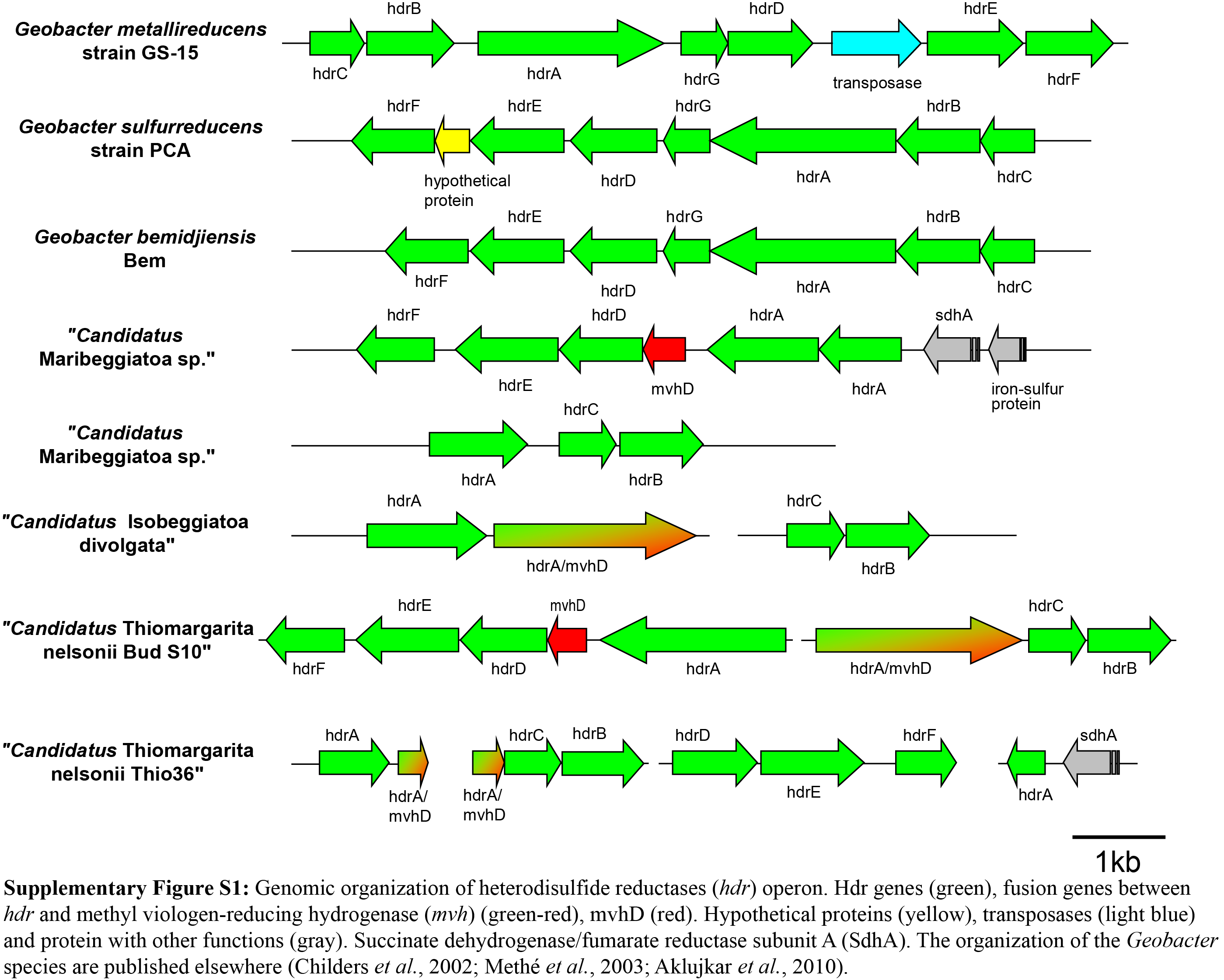

Supplement: Supplementary file 2 [file Image_1.TIF]

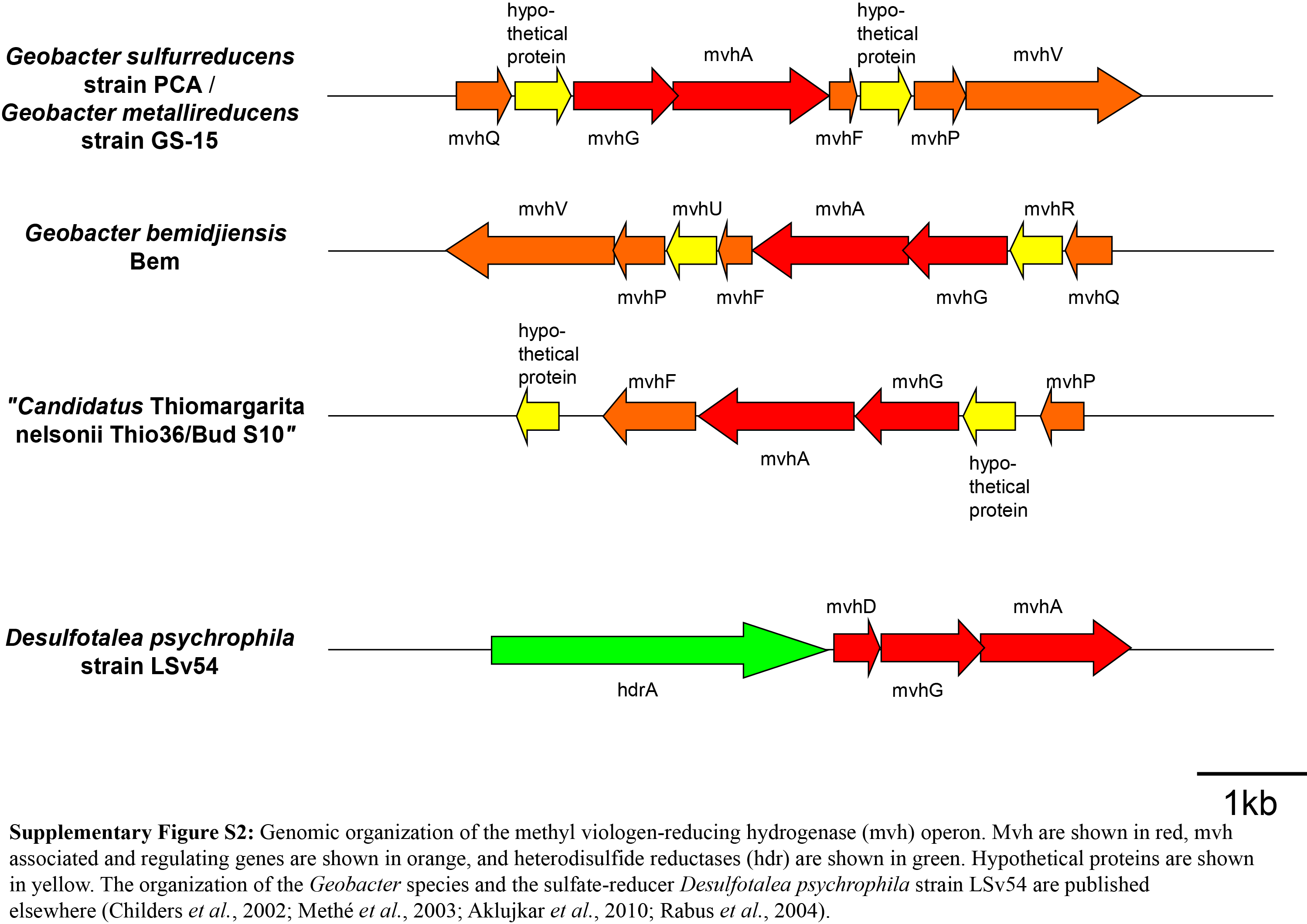

Supplement: Supplementary file 3 [file Image_2.TIF]
